# From Delta to Omicron—Genetic Epidemiology of SARS-CoV-2 (hCoV-19) in Southern Poland

**DOI:** 10.3390/pathogens14070708

**Published:** 2025-07-17

**Authors:** Maria Miklasińska-Majdanik, Emilia Morawiec, Jolanta Bratosiewicz-Wąsik, Karol Serwin, Adam Pudełko, Michał Czerwiński, Anna Bednarska-Czerwińska, Miłosz Parczewski, Tomasz J. Wąsik

**Affiliations:** 1 Department of Microbiology, Faculty of Pharmaceutical Sciences in Sosnowiec, Medical University of Silesia in Katowice, 40-055 Katowice, Poland; mmiklasinska@sum.edu.pl (M.M.-M.); jbrat@sum.edu.pl (J.B.-W.); 2 Department of Microbiology, Faculty of Medicine in Zabrze, Academy of Silesia in Katowice, 40-555 Katowice, Poland; e.morawiec@gyncentrum.pl; 3 Gyncentrum, Laboratory of Molecular Biology and Virology, 40-851 Katowice, Poland; a.pudelko@gyncentrum.pl (A.P.); m.czerwinski@gyncentrum.pl (M.C.); czerwinskaa002@gmail.com (A.B.-C.); 4 Department of Infectious, Tropical Diseases and Acquired Immunodeficiency, Pomeranian Medical University in Szczecin, 70-204 Szczecin, Poland; karol.serwin@pum.edu.pl (K.S.); mparczewski@yahoo.co.uk (M.P.); 5 Department of Immunology, Faculty of Medicine in Zabrze, Academy of Silesia in Katowice, 40-055 Katowice, Poland; 6 American Medical Clinic, 40-851 Katowice, Poland; 7 Department of Gynecology and Obstetrics, Faculty of Medicine in Zabrze, Academy of Silesia in Katowice, 40-055 Katowice, Poland; 8 Department of Medical Microbiology, Faculty of Medical Sciences in Katowice, Medical University of Silesia in Katowice, 40-055 Katowice, Poland

**Keywords:** SARS-CoV-2 variants, molecular epidemiology, whole-genome sequencing

## Abstract

Since severe acute respiratory syndrome coronavirus 2 (SARS-CoV-2) was first reported in Wuhan, China, in December 2019, it has evolved, leading to variants that differ in their transmissibility, severity of disease, and susceptibility to therapy. Our goal was to describe the dynamics of the emergence of SARS-CoV-2 variants among the population of the southern part of Poland (Silesia) in the period from September 2021 to August 2022. Our results showed that, like in the rest of Poland or in neighboring countries (Czech Republic, Slovakia), Delta was replaced by the Omicron BA.1 variant, isolated for the first time in December 2021, and subsequently Omicron BA.2 and its derivative subvariants acquiring further mutations. Finally, in August 2022, only the BA.5.2.26 subvariant was present in Silesia. However, we noted differences in the dynamics of emergence and spread of some Omicron subvariants compared to the rest of Poland and the neighboring countries, which may be due to differences in population density or import of the virus from other regions.

## 1. Introduction

The continuous monitoring of the spread of the SARS-CoV-2 virus, which causes severe acute respiratory syndrome, started after the first case was reported in late 2019 in Wuhan, China. By analyzing the complete genetic profile of the virus, researchers were able to track the outbreak in real time and gain valuable insights into its evolution. The COVID-19 pandemic has led to a significant increase in sequencing efforts across Europe, resulting in a larger number of publicly available SARS-CoV-2 genetic sequences [1,2,3,4,5].

As of February 2024, over 16,536,347 complete genomes of SARS-CoV-2 have been recorded in the Global Initiative on Sharing All Influenza Data (GISAID) database [5]. This comprehensive molecular monitoring of the virus necessitates a simple method for categorizing variant genetic variations. Extensive molecular data allows for the real-time tracking of the pandemic’s evolution through the examination of outbreaks and surveillance of the emergence of new circulating strains [6,7]. The B.1.617.2 + AY (Delta) variant of the SARS-CoV-2 virus initially appeared during India’s second wave, then rapidly spread and became the prevailing variant worldwide. However, it still continues to undergo evolutionary changes. On 26 November 2021, the World Health Organization designated a new variant, B.1.1.529, as a cause for concern and named it Omicron. This decision was based on the discovery of several mutations in Omicron, which may potentially impact its behavior [8].

The emergence of new SARS-CoV-2 variants in the human population is driven in part by mutations in the spike (S) protein, particularly in the receptor-binding domain (RBD), some of which facilitate tighter binding to angiotensin-converting enzyme (ACE2). Various structure-based bioinformatics tools, like Gibbs free energy (ΔG) values estimation, DynaMut [9], MAESTROweb [10], and SDM [11] have been used to study the effects of individual mutations on the stability of SARS-CoV-2 proteins. As an example, studies of one of the earliest genomic evolutions identified as the D614G substitution, which is present in all SARS-CoV-2 B.1 lines, showed that it is a stabilizing mutation [12] and is associated with enhanced transmissibility of SARS-CoV-2 strains [13,14]. Other analyses, including eight significant mutations in Omicron, revealed that D614G, Q493K, and S477N mutations showed stabilizing effects, while E484A, N501Y, K417N, Y505H, and G496S substitutions were destabilizing. What is more, the D614G, E484A, N501Y, K417N, Y505H, and G496S mutations increased the molecular flexibility of S-glycoprotein to interact with the ACE2 receptor, increasing the variant’s infectivity [15]. Interestingly, calculation of the binding free energy of RBD-ACE2 complexes formed by different variants of RBD showed the most stable dynamic of the Omicron RBD-ACE2 complex, suggesting that substitution mutations in the Omicron RBD may increase the stability of the RBD-ACE2 complex, and the Delta RBD-ACE2 complex was slightly less stable than Omicron [16].

Our previous research showed a genetic analysis of the epidemiology of SARS-CoV-2 (hCoV-19) in Southern Poland, focusing on the period from February 2021 to August 2021. At that time, the Alpha variant dominated in Silesia, later replaced by the Delta variant [17]. The aim of the presented study was to investigate the molecular epidemiology of SARS-CoV-2 lineages in Southern Poland from September 2021 to 31 August 2022. Through comprehensive analysis of 942 viral genomes, including whole-genome sequencing, variant identification, and phylogenetic analysis, we observed the evolutionary trends of these variants. Additionally, we compared our data from Southern Poland with sequences from Poland, as well as neighboring countries such as the Czech Republic and Slovakia.

## 2. Materials and Methods

### 2.1. Study Group

From September 2021 to August 2022, a total of 942 SARS-CoV-2 positive samples confirmed by RT-PCR were analyzed using various diagnostic kits, including MediPAN 2G + FAST COVID Kit (Medicofarma, Radom, Poland), 2019-nCoV Triplex RT-qPCR Detection Kit (Vazyme, Biotech, Nanjing, China), MutaPLEX Coronavirus Real-Time RT-PCR Kit (Immunodiagnostik AG, Bensheim, Germany), and GeneXpert assays (Xpert Xpress SARS-CoV-2 and Xpert Xpress SARS-CoV-2/FLU/RSV, Cepheid, Sunnyvale, CA, USA). All procedures were performed in accordance with the manufacturers’ protocols. The majority of the samples originated from the Silesian region in Southern Poland and were collected from a range of sources: primary and secondary hospital wards, hospital-based SARS-CoV-2 diagnostic sites, and mobile SARS-CoV-2 testing units. In total, specimens and associated data were provided by 31 different facilities. All data were anonymized before being included in the analysis. Only samples with an RT-PCR cycle threshold (CT) value below 32 were selected for sequencing. Viral lineages were determined using the Pangolin v.4.3.1 tool and further confirmed with the Nextclade platform.

### 2.2. SARS-CoV-2 Whole-Genome Sequencing (WGS)

SARS-CoV-2 RNA was extracted using the Maxwell 48 RSC system and the corresponding Viral TNA kit (both from Promega Corporation, Madison, WI, USA). The quality and concentration of the isolated RNA were assessed with a Quantus fluorometer (Promega Corporation, Madison, WI, USA) and a Fragment Analyzer 5200 system (Agilent Technologies, Santa Clara, CA, USA). Reverse transcription and PCR amplification steps were carried out using the CFX96 Real-Time System and C1000 Touch Thermal Cycler platforms (Bio-Rad Laboratories, Hercules, CA, USA). For sequencing, two different library preparation approaches were employed: the Illumina RNA Prep with Enrichment method using the Respiratory Virus Oligo Panel (RVOP) for 856 samples, and the NEBNext ARTIC SARS-CoV-2 FS Library Prep Kit (New England Biolabs, Ipswich, MA, USA) for 48 samples. The final sequencing was conducted on Illumina’s miSeq and NextSeq 1000 platforms, both designed for high-throughput next-generation sequencing (NGS). RNA isolation, reverse transcription, and library preparation were performed according to the manufacturers’ protocols, and strict quality control was applied at each step to ensure the integrity and concentration of RNA material used in sequencing. Only samples with sufficient RNA quality (assessed by integrity profiles and concentrations) proceeded to library preparation. Following sequencing on Illumina NGS platforms (Illumina, Inc., San Diego, CA, USA), the resulting data were processed using DRAGEN analysis pipelines. Specifically, the DRAGEN COVID Lineage tool (version 3.5.3, configured with a maximum ambiguous rate of 0.5 for Pangolin lineage assignment) and the DRAGEN RNA Pathogen Detection pipeline (version 3.5.16, using the SARS-CoV-2 reference genome NC_045512.2 and the human genome reference hg38) were employed. The Illumina Respiratory Virus Panel was also used, incorporating internal human control sequences. Each sample was evaluated based on several quality parameters, including genome coverage, number of mapped and raw reads, read duplication rate, sequence length, and percentage of undetermined bases (%N). Pangolin and Nextclade tools were used for SARS-CoV-2 lineage assignment and mutation verification. Lineages were confirmed by assessing mutation patterns and phylogenetic context. Consensus sequences not meeting the required thresholds (e.g., coverage <90%, excessively ambiguous bases) were excluded from the final reporting. Raw sequencing data, along with accompanying metadata—such as sample collection date, geographic origin within Poland, patient sex and age, and clinical status (when available)—were consistently submitted to the GISAID database (https://gisaid.org/, access from 1 October 2021 to 31 September 2022) following analysis. All sequencing and analysis steps were performed in accordance with protocols recommended for SARS-CoV-2 genomic surveillance, ensuring the reproducibility and reliability of the results.

### 2.3. Phylogenetic Analyses

To complete phylogenetic analyses, we originally compiled a dataset of 135,879 SARS-CoV-2 genomes (covering the period from 1 September 2021 to 31 August 2022) based on data available as of 25 July 2023, from GISAID (https://gisaid.org/) (accessed on 25 July 2023)). For this investigation, we included sequences specifically from the Silesia region (n = 942), along with data from three countries: (i) Poland (excluding Silesian sequences, n = 62,520), (ii) Czech Republic (n = 39,579), and (iii) Slovakia (n = 32,838). Given the unknown proportion of Delta and Omicron cases in those countries, we subsampled these genomes based on the proportion of overall COVID-19 cases reported per epidemiological week in each country, using reports from the “Center for Systems Science and Engineering (CSSE)” Johns Hopkins University (http://github.com/CSSEGISandData/COVID-19 (accessed on 10 September 2022). The subsampling process utilized the ‘subsampler’ pipeline (http://github.com/andersonbrito/subsampler (accessed on 10 September 2022), selecting genomes to simulate a scenario where 0.1% of cases per ‘epiweek’ per country were sequenced. The final dataset comprised 8,576 virus genomes from three countries: (i) Poland (excluding Silesian sequences, n = 3096), (ii) Czech Republic (n = 2707), and (iii) Slovakia (n = 1831), including the unbiased sequences from the Silesia region. All genomes had coverage above 70% and represent the COVID-19 burden as indicated by each country’s epidemiological time series data. The complete list of genomes is available in Supplementary Table S1.

Using the augur pipeline, we conducted a rigorous multiple sequence alignment (MSA) using MAFFT [4]. We carefully masked the MSA’s 5′ and 3′ ends alongside other problematic sites [18] using a script delivered with the pipeline. Then, we performed a quick maximum likelihood analysis using IQ-Tree [19] under a GTR nucleotide substitution model. Divergence times and ancestral states were inferred using TreeTime 0.8.0 [20]. This preliminary analysis was about determining the placement of the virus genomes and identifying any major molecular clock outliers deviating from more than four interquartile ranges in the root-to-tip regression line. We inferred the final time-scaled tree to identify large clades containing only genomes of Silesian and Polish, Silesian and Czech, and Silesian and Slovakian origin. These clades are significant in our collective understanding of the spread and evolution of the SARS-CoV-2 variants in the investigated regions.

### 2.4. Epidemiological Data Analysis

Epidemiological data and statistics for the Czech Republic and Slovakia were downloaded from the public repository Our World in Data. Data for Poland and the Silesian Voivodeship were downloaded from government reports posted at https://www.gov.pl/web/koronawirus (accessed on 10 February 2023). These data sets provided the necessary case counts and temporal information used to calibrate the subsampling process and contextualize the spread of SARS-CoV-2 variants in the studied regions. All data were cross-checked for consistency and aligned to epidemiological weeks to ensure accurate comparisons across countries.

## 3. Results

### 3.1. The Prevalence of SARS-CoV-2 Variants (hCoV-19) in Southern Poland

**Figure 1:**
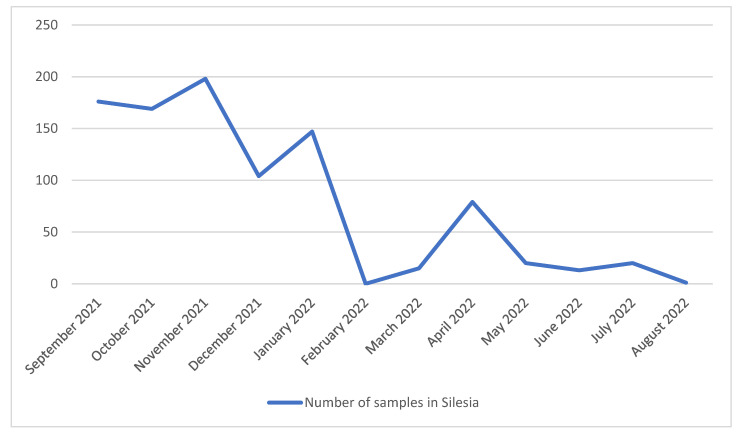
Changes in the number of SARS-CoV-2 samples isolated from September 2021 to August 2022 in Silesia.

**Figure 2:**
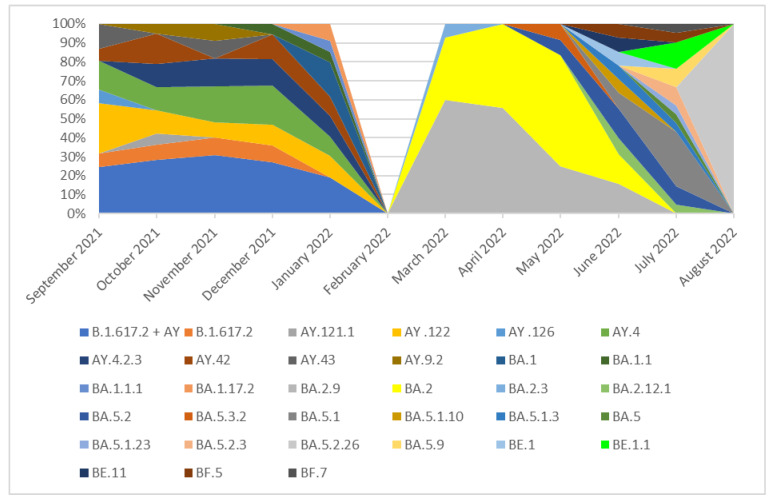
The percentage distribution of SARS-CoV-2 variants isolated in Southern Poland from 1 September 2021 to 31 August 2022 based on whole-genome sequencing.

From the 942 sequenced samples collected from September 2021 to 31 August 2022 in Southern Poland (Silesia), 939 were analyzed and classified into variants while three were rejected due to the large number of missing nucleotides in sequences (>1500). It should be noted that the number of analyzed samples has decreased significantly since February 2022, and in February 2022 no sample was collected. Far fewer samples have been isolated since March 2022. This was directly related to the changes in COVID-19 testing policy introduced in Poland on 28 January, where antigen tests for SARS-CoV-2 were allowed to be used in laboratories/mobile collection points [21] and the need for isolation, quarantine, and mask-wearing policy in Poland was lifted [22]. Changes in the number of samples isolated in individual months in Silesia are presented in Figure 1. In the period from September 2021 to January 2022, the Delta (B.1.617.2) variant was most frequent as a group. The Delta lineages which occurred in these months with a frequency of >5% were B.1.617.2, AY.122, AY.4, AY.4.2.3, and AY.42. None of these lineages was dominant, and all of them were recorded with similar frequency (from 8% to 26%). In addition, several cases of lineages AY.121.1, AY.126, AY.43, and AY.9.2 were recorded during this period. The remaining Delta lineages with a frequency of less than 5%, which are not individually listed, have been grouped together under the label B.1.617.2 + AY in the chart for simplicity (Figure 2).

In March 2022, the B.1.617.2 + AY variant was replaced by the B.1.1.529 + BA variant group, which completely dominated lineages circulating in Silesia in the following months. Since all Omicron sublineages included in this group had individual frequencies above 5%, they are presented separately in the figure rather than grouped under a single label. In March and April 2022, the dominant lineage was BA.2.9 (60% and 52%, respectively). In May, this lineage was superseded by BA.2 (35%). In June 2022, there was no clearly dominant variant, and lineages BA.2 (15%), BA.2.9 (15%), BA.2.12.1 (8%), BA.5.1 (8%), BA.5.2 (15%), BA.5.1.10 (7%), BA.5.1.3 (7%), BE.1 (7%), BE.11 (7%), and BF.5 (7%) were isolated. In July, the BA.5.2 lineage was noted in 30% of cases. Other variants included BA.2.12.1 (5%), BA.5.1 (10%), BA.5.1.3 (5%), BA.5 (5%), BA.5.1.23 (5%), BA.5.2.3 (10%), BA.5.9 (10%), BE.1.1 (15%), BF.5 (5%), and BF.7 (5%). One sample was isolated in August 2022 and identified as BA.5.2.26 (100%).

The analysis of the collected data suggests that the diversity of variants circulating in the studied population was very large. The percentage distribution of the main SARS-CoV-2 variants (frequency > 5%) isolated in Silesia from September 2021 to August 2022 are presented in Figure 2.

### 3.2. The Prevalence of SARS-CoV-2 Main Variants (hCoV-19) in Poland, the Czech Republic, and Slovakia

**Figure 3:**
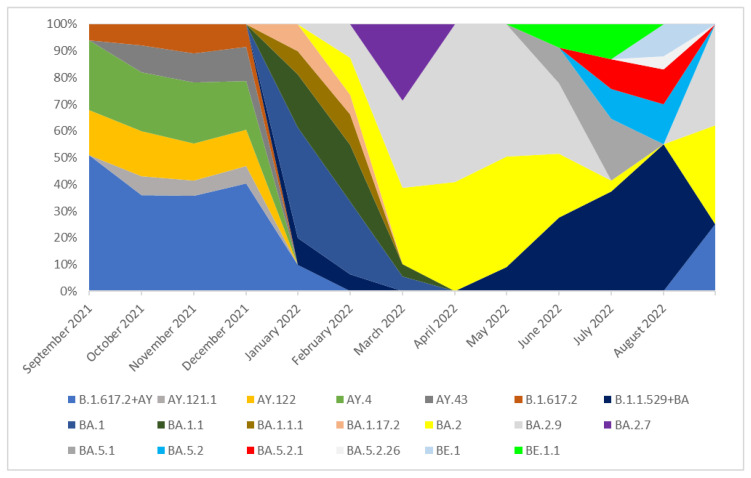
The percentage distribution of SARS-CoV-2 variants isolated in Poland from 1 September 2021 to 31 August 2022 based on whole-genome sequencing.

**Figure 4:**
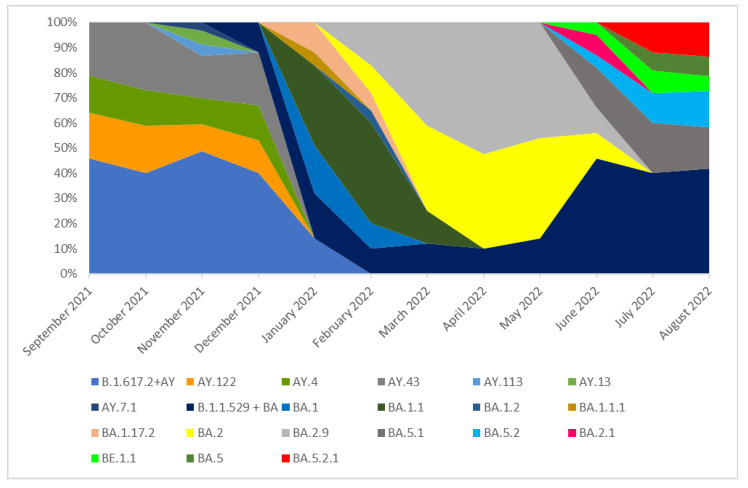
The percentage distribution of SARS-CoV-2 variants isolated in the Czech Republic from 1 September 2021 to 31 August 2022 based on whole-genome sequencing.

**Figure 5:**
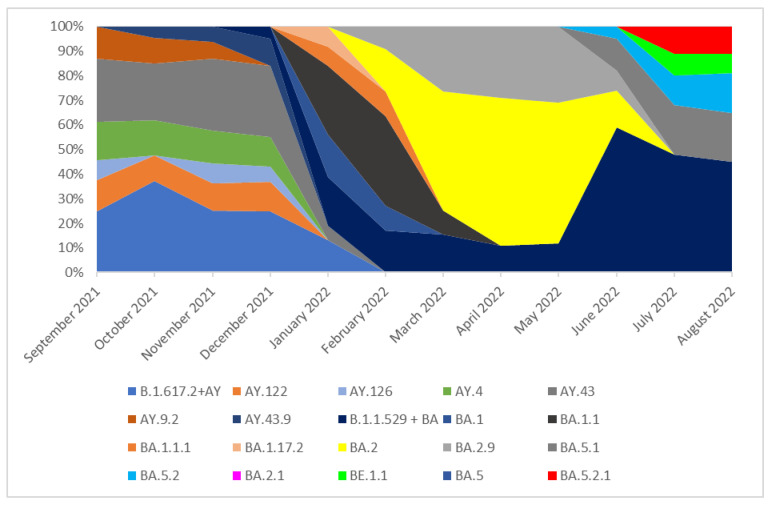
The percentage distribution of SARS-CoV-2 variants isolated in Slovakia from 1 September 2021 to 31 August 2022 based on whole-genome sequencing.

The analysis of whole-genome sequences from Poland, Slovakia and the Czech Republic was carried out based on the data available in GISAID. The diversity of variants in the studied period was very large in all countries. None of the variants clearly dominated over the others. The percentage distribution of the main SARS-CoV-2 variants (frequency >5%) isolated in Poland, the Czech Republic and Slovakia from September 2021 to August 2022 are presented in Figure 3, Figure 4 and Figure 5.

An analysis of 65,509 sequences reported in GISAID between September 2021 and 31 August 2022 in Poland was performed. In the period from September 2021 to December 2021, the Delta (B.1.617.2 + AY) variant was dominant and lineages which occurred with these months in a frequency of >5% were B.1.617.2, AY.121.1, AY.122, AY.4, and AY.43. None of these lines was dominant, and all of them were recorded with similar frequency (from 6 to 26%). The remaining lineages of the Delta variant, occurring with a frequency of <5%, are marked as B.1.617.2 + AY in the chart for clarity (Figure 3). In January 2022, the B.1.617.2 + AY variant was replaced by the B.1.1.529 + BA which completely dominated lineages circulating in Poland in the next analyzed months. Since all Omicron sublineages included in this group had individual frequencies above 5%, they are presented separately in the figure rather than grouped under a single label. In January and February 2022, the dominant variant was the BA.1 line and next the BA.1.1 variant. From March to June 2022, the dominant lineages were BA.2 and BA.2.9. The variant BA.2.7 (36%) was also observed with high frequency in March. In July and August 2022, neither of these lineages was dominant, and all of them were recorded with similar frequency.

In the Czech Republic, 39,579 samples were collected. In the period from September 2021 to January 2022, the Delta (B.1.617.2 + AY) variant was dominant and lineages which occurred in these months in a frequency of >5% were AY.122, AY.4, and AY.43. None of these lineages was dominant, and all of them were recorded with similar frequency (from 13 to 27%). In addition, several cases of lineages AY.113, AY.13, and AY.7.1 were recorded during this period. Delta lineages with a frequency below 5% that are not shown separately have been combined under the label B.1.617.2 + AY in the chart for clarity (Figure 4). In January 2022, the B.1.617.2 + AY variant was replaced by the B.1.1.529 + BA which completely dominated lineages circulating in the Czech Republic in the next analyzed months. In February 2022, the dominant lineage was BA.1.1 (40%). In March this lineage was superseded by BA.2 (35%). From March to May 2022, the dominant variants were BA.2 and BA.2.9. From June to August 2022, neither of these lineages was dominant, and all of them were noted with similar frequency. Omicron lineages occurring at a frequency below 5% and not listed individually have been combined under the label B.1.1.529 + BA in the chart for clarity (Figure 4).

In Slovakia, 32,838 samples were collected. In the period from September 2021 to December 2021, the Delta (B.1.617.2 + AY) variant was most frequent and lineages which occurred in these months with a frequency of >5% were AY.122, AY.126, AY.4, AY.43, AY.9.2, and AY.43.9. None of these lineages was dominant, and all of them were recorded with similar frequency (from 5 to 9%). The remaining Delta lineages with a frequency of less than 5%, which are not individually listed, have been grouped together under the label B.1.617.2 + AY in the chart for simplicity (Figure 5). In January 2022, the B.1.617.2 + AY variant was replaced by B.1.1.529 + BA which completely dominated lineages circulating in Slovakia in the next analyzed months. In February 2022, the dominant lineage was BA.1.1 (36%). In March, this lineage was superseded by BA.2 (50%). From March to May 2022, the dominant variant was BA.2. The BA.2.9 variant also occurred with high frequency. From June to August 2022, neither of these lineages was dominant, and all of them were recorded with similar frequency. The remaining Omicron lineages with a frequency of less than 5%, which are not individually listed, have been grouped together under the label BA.1.1.529 + AY in the chart for simplicity (Figure 5).

### 3.3. The Phylogenetic Connections of SARS-CoV-2 Clades

**Figure 6:**
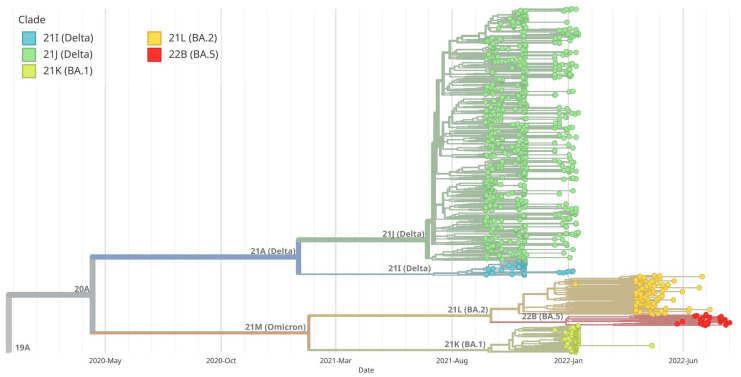
Maximum likelihood (ML) phylogenetic analysis of SARS-CoV-2 complete genome sequences in Southern Poland. The branches corresponding to the five primary clades are highlighted in blue, dark green, light green, orange and red. Virus variants were classified using the Nextstrain lineage systems. The variant 21J (Delta) has definitely dominated in Poland since August 2021, while the 21M (Omicron) variant began to displace 21J in January 2022. Initially, it was the BA.1 variant gradually replaced by a BA.2 variant and then a BA.5 variant.

**Figure 7:**
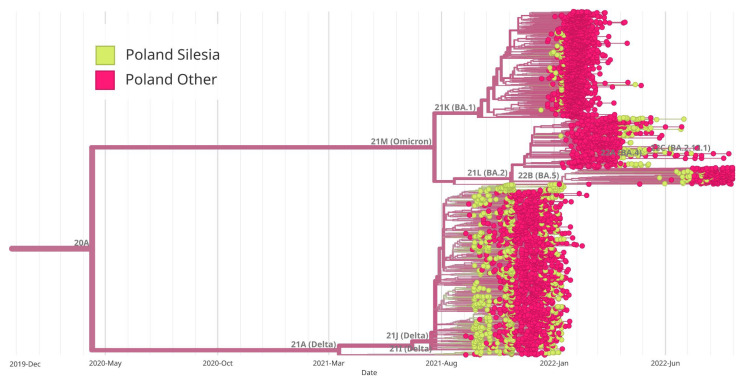
Maximum likelihood (ML) phylogenetic analysis of SARS-CoV-2 complete genome sequences in Silesia within the broader context of Poland. Variants corresponding to Silesia are marked in green, while those corresponding to the rest of the country are marked in red. Virus variants were classified using the Nextstrain lineage systems. Variant 21A (Delta) appeared earlier in Silesia than in the rest of the country, while the BA.1 variant (Omicron) began to appear at the same time both in Silesia and throughout Poland. On the other hand, the BA.2 variant appeared earlier in other regions of Poland, and later in Silesia. The BA.5 variant dominated Silesia earlier than the rest of Poland.

**Figure 8:**
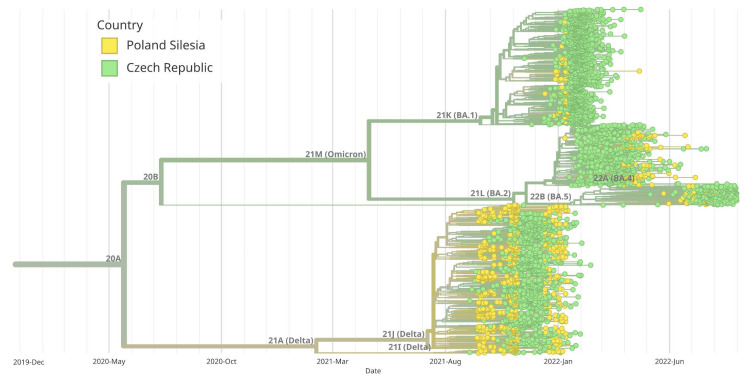
Maximum likelihood (ML) phylogenetic analysis of SARS-CoV-2 complete genome sequences in Silesia compared to the Czech Republic. Variants corresponding to Silesia are marked in yellow, while those corresponding to the Czech Republic are marked in green. Virus variants were classified using the Nextstrain lineage system. Variant 21A (Delta) appeared earlier in Silesia than in the Czech Republic. BA.1 and BA.5 variants began to appear at the same time both in Silesia and the Czech Republic, while the BA.2 variant (Omicron) dominated the Czech Republic earlier than Silesia.

**Figure 9:**
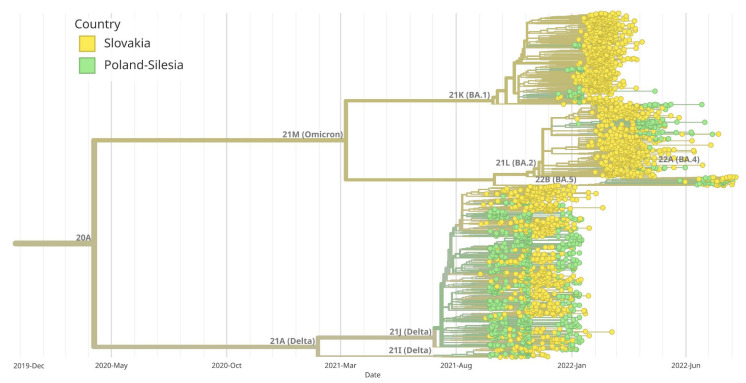
Maximum likelihood (ML) phylogenetic analysis of SARS-CoV-2 complete genome sequences in Silesia compared to Slovakia. Variants corresponding to Silesia are marked in green, while those corresponding to Slovakia are marked in yellow. Virus variants were classified using the Nextstrain lineage system. Variant 21A (Delta) appeared at the same time in Silesia and Slovakia. The BA.1 and BA.5 variants began to appear earlier in Silesia than in Slovakia, while the BA.2 variant dominated Slovakia earlier than Silesia.

The analysis of the genetic history of the samples tested in Southern Poland showed that there were two main groups of the SARS-CoV-2 virus, namely B.1.617.2 + AY and B.1.1.529 + BA. The BA.1 group was derived from B.1.1.529 + BA, similar to BA.2. Figure 6 provides a visual representation of the evolutionary relationships among different clades of SARS-CoV-2 observed in Southern Poland. In addition, we created a diagram showing the evolutionary relationships of Silesia’s clades within the broader context of Poland (Figure 7). Furthermore, a thorough examination comparing Silesia with the Czech Republic and Slovakia was conducted (Figure 8 and Figure 9). The analysis of the trees presented in Figure 7, Figure 8 and Figure 9 allows us to conclude that in each case, the dominance of the B.1.617.2 + AY variant was observed, which was gradually replaced by the B.1.1.529 + BA variant.

### 3.4. COVID-19 Cases in Silesia Compared to the Rest of Poland and Neighboring Countries

According to Our World in Data, during the period covered by the present study Poland had 1.2 times more COVID-19 cases than Czech Republic and approximately 2.3 times more than Slovakia. However, after adjustment for the country’s population, the number of COVID-19 cases per million inhabitants in Poland was 2.8 times fewer than in Czech Republic and Slovakia. In contrast, we did not observe any differences in the number of COVID-19 cases per 1 million inhabitants between the Silesian Voivodeship and the country-wide area.

## 4. Discussion

The Silesian region is inhabited by more than 4.3 million people, which is about 12% of the Polish population. The population density in the Silesian Voivodeship is nearly three times higher compared to the average population density in Poland (355 vs. 121 persons per 1 km^2^). Moreover, the Silesian Voivodeship borders both the Czech Republic and Slovakia and before the pandemic began, thousands of people benefited from the cross-border labor market—many residents of the Silesian region worked in Czech and Slovak companies. After the Polish government introduced mandatory border controls and a two-week quarantine for Poles returning to the country from abroad in March 2020, full border traffic within the internal borders of the European Union was restored on 13 June 2020. Thus, during the period covered by our research, cross-border traffic was no longer restricted, allowing the free circulation of virus variants between neighboring countries.

**Figure 10:**
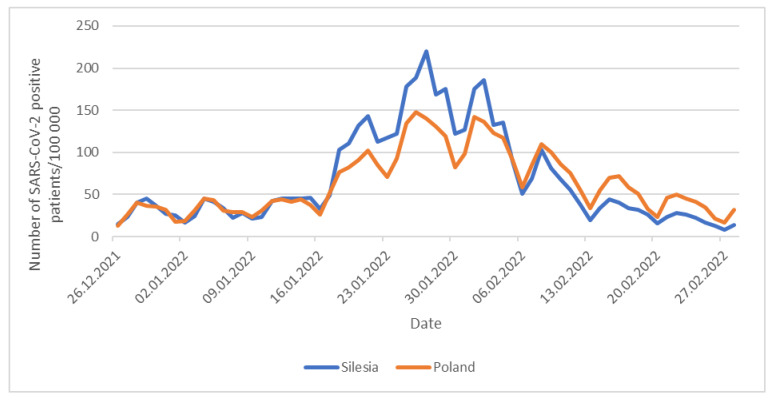
Daily number of SARS-CoV-2 positive cases per 100,000 inhabitants.

In the period from September 2021 to January 2022, the Delta variant (B.1.617.2 + AY) dominated in Southern Poland. It was consistently replaced by the Omicron B.1.1.529 + BA variant. Contrasting Silesia with Poland, the Omicron variant appeared in Southern Poland earlier. It was first recorded in December 2021 (BA.1.1), while in the rest of the country it appeared in January 2022 and caused the fifth wave which peaked at the end of January 2022 [23]. The earlier appearance of the Omicron variant in the Silesian province may be responsible for different dynamics of the development of the fifth wave of the pandemic in this region compared to the rest of the country (Figure 10). Namely, during the initial phase of the fifth wave of the pandemic, in the second half of January 2022, the incidence rate of positive cases (per 100,000 inhabitants) was more dynamic in Silesia compared to the rest of Poland. Then, in February, a faster decrease in the number of new infections per 100,000 inhabitants was observed in our region compared to Poland as a whole. This early and rapid spread of the Omicron variant of SARS-CoV-2, combined with significant population density, may explain why the presence of the Delta variant of the virus was no longer observed in Silesia in March.

In March and April, the dominant variant both in Silesia and the rest of the country was BA.2.9. The BA.2 variant occurred with a similar frequency. Interestingly, in the given period, the BA.2.7 variant also appeared with high frequency in Poland, but it was not noted in Silesia. Since June 2022, a large variety of variants without any specific dominance has been observed in Southern Poland. A similar situation occurred throughout Poland.

**Figure 11:**
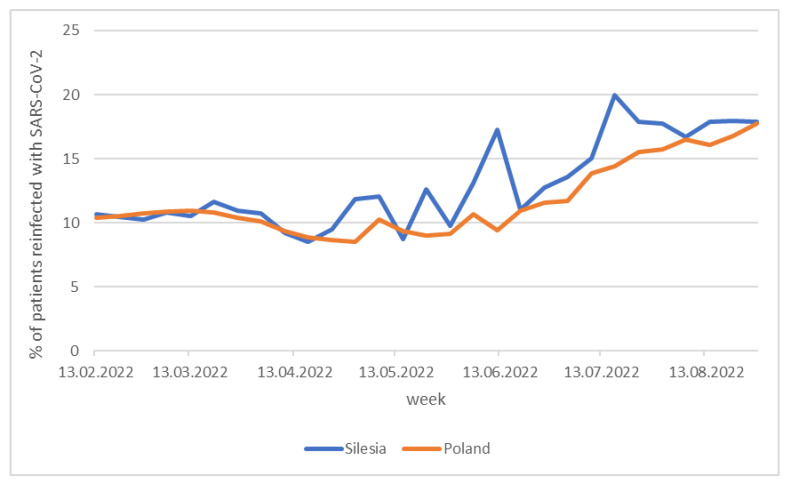
Weekly percentage of SARS-CoV-2 reinfected patients.

According to the data available on the government website, during the period from early May to mid-July, considerably more cases of reinfection were observed in the region covered by our research compared to the rest of the country (Figure 11), which is especially noteworthy. In Silesia, BA.5 subvariants (BA.5.2 and BA.5.32) appeared as early as May, while in the rest of the country the presence of BA.5.1, BA.5.2, and BA.5.2.1 subvariants was recorded two months later, in July. The BA.5 clades stem from BA.2 by acquisition of Δ69-70 deletion in the N-terminal domain, L452R, F486V, and reversed R493Q mutations in the receptor-binding region, S704L mutation located outside the receptor-binding region of the spike protein, as well as D3N mutation in the M protein.

Previously conducted studies have shown that BA.5 competed with BA.2 because of its increased resistance to neutralization [24,25,26] and its higher infectivity resulting from higher replication fitness, transmissibility, and pathogenicity [27,28,29]. The functional examination of the impact of mutations characterizing BA.5 S proteins showed that mutations of Δ69-70, L452R, and F486V contribute to the higher infectiousness and fusogenicity of the BA.5 S protein. Moreover, L452R and F486V substitutions were responsible for reduced sensitivity to neutralizing antibodies [30].

Other studies have shown that antibodies isolated from BA.1-infected or -vaccinated individuals showed reduced efficacy against L452 mutations, causing the most severe escape of variants BA4/BA.5 harboring L452R mutation [26], which may explain the ability of the BA.5 variants to evade the neutralizing effect of antibodies arising from previous infections. The importance of the L452R mutation for impairing antibody binding, allowing the B5 variant to escape the immune system, was also demonstrated by Tuekprakhon et al. [31]. Moreover, the S371F mutation (occurring in Omicron but not the Delta variant) was involved in the emergence of conformational changes of the region recognized by antibodies isolated from vaccinated individuals who had recovered from SARS [26].

What is more, in June and July in the Silesia region we observed the occurrence of BA.2.12.1, another derivative of the BA.2 variant arising by acquisition of L452Q and S704L mutations in addition to the known mutations in BA.2. The Omicron BA 2.12.1 subvariant appeared in December 2021 in the United States and spread very fast in many parts of the US, but also in other countries [32,33]. Many studies showed that BA.2.12.1, like the BA.4 and BA.5 subvariants, substantially escaped neutralizing antibodies induced by both vaccination and infection. Compared to the BA.2 Omicron variant, the BA.2.12.1 subvariant shows stronger immune escape and faster transmissibility. In contrast to BA.5, the BA.2.12.1 Omicron subvariant showed only modest resistance to sera from vaccinated and boosted individuals compared to BA.2 [25]. Reduced sensitivity of BA.2.12.1 compared to BA.2 S protein to neutralization by sera of vaccinated donors was found; however, this effect was donor-dependent. On the contrary, S704L mutation increased susceptibility to neutralization [30]. Of note, the BA.2.12.1 variant has not been detected either in the rest of Poland or in neighboring countries (Czech Republic, Slovakia); therefore, it was probably imported to Silesia from other regions of Europe or the world. In Silesia, BA.2.12.1 was completely replaced by BA.5.2.26 in August, which reflects a transmission advantage of BA.5 subvariants. The dominance of previously circulating variants of the virus by more efficient variants has also been observed in other regions, e.g., South Korea, where BA.1 was the dominant variant for 10 weeks starting from the 1st week of 2022, BA.2 was the dominant variant in the 12th and 13th weeks of 2022, and they were subsequently replaced by BA.5 which became the dominant variant in June 2022 [34].

In the Czech Republic, the Omicron variant was first noted in December 2021. An earlier dominance of the Omicron variants was also observed, which already replaced the Delta variants in January 2022, when this variant was just appearing in Silesia. Similarly to Silesia and the rest of Poland, the dominance of the BA.2.9 variant has also been observed since March 2022. Since June, the diversity of variants in the Czech Republic has been very large.

Similarly in Slovakia, we observed the earlier displacement of the Delta variant by Omicron, which dominated already in January 2022. What is more, comparing Slovakia with Silesia, a clear difference can be noticed, consisting in the dominance of the BA.2 variant from March to May 2022 instead of the BA.2.9 variant.

When comparing the molecular diversity of SARS-CoV-2 in Silesia with the above regions, the difference in the occurrence of variants of the BF (BF.5 and BF.7) line locally in Silesia should also be noted. What is more, we observed greater diversity among BE line variants (BE.1, BE.1.1 and BE.11).

Analyzing the dynamics of the appearance of individual variants globally, it can be concluded that the Omicron variant appeared earlier in the world than in the regions discussed in this work. It was first sequenced in November 2021 in South Africa, while in Silesia it appeared in December 2021, as well as in the Czech Republic and Slovakia. In other regions of our country, it was detected in January 2022 [35]. An interesting aspect seems to be the earlier appearance of the Omicron variant in Silesia than in the rest of Poland. It seems likely that the variants moved from our southern neighbors into the country. It should be noted, however, that not all samples were reported in GISAID and it cannot be ruled out that Omicron had occurred in other regions of Poland earlier.

Skuza et al. conducted an active monitoring program among military personnel to identify Variants of Concern (VOC) of the SARS-CoV-2 virus, with a particular focus on overseas military operations. Screening of 1699 soldiers using RT-qPCR tests was conducted between November 2021 and May 2022. Out of these, 84 samples tested positive for SARS-CoV-2 and met the criteria for whole-genome sequencing analysis for variant identification [36]. Based on analysis of samples from 79 soldiers tested in Poland between November 2021 and March 2022, it can be inferred that the obtained results match the molecular analysis of SARS-CoV-2 in Silesia. Until December 2021, the Delta variant dominated, and then it was replaced by the Omicron lineage from January to March 2022. Similarly to Silesia, the variants BA.1, BA.1.1, BA.1.1.1, and BA.2.9 were dominant in this period. The BA.2.3 variant which was observed in our region in March did not appear in the study by Skuza et al. Additionally, the population studied by Skuza et al. was characterized by greater molecular diversity. What is more, in March, the Delta variant still appeared there, though it had already been completely replaced by Omicron in Silesia during this period [36]. This difference may be due to the fact that Omicron was detected in Silesia as early as December 2021, while in the cohort studied by Skuza this variant was observed only in January 2022. Similar clades were identified in 89 soldiers deployed from Romania in February and March 2022. However, the BA.1.1.13 variant occurred more frequently than among Polish soldiers and samples isolated in Silesia. Similarly, among soldiers returning from France between February and June 2022, the BA.2.9 variant predominated. Additionally, Skuza et al. found a high frequency of the BA.2.56 line, which did not occur in Silesia. Skuza et al.’s findings indicate that all genetic variants of SARS-CoV-2 found in Polish Armed Forces members had already been circulating in Poland prior to their return from their missions. This suggests that there was minimal transmission of these variants within the national population, or any transmissions that did occur did not significantly impact the SARS-CoV-2 variants present in Poland.

SARS-CoV-2 is constantly evolving, and acquired mutations contribute to the emergence of different variants. The risk associated with the emergence of a new variant of SARS-CoV-2 is determined by its ability to transmit, the severity of the induced disease, the evasion of immunity, as well as diagnostic and therapeutic escape compared to the other initial strains. Consecutive variants of the virus have arisen from the accumulation of mutation combinations, among which mutations in the spike protein are crucial for the life cycle of the virus [37]. The rapid spread of the virus, particularly the Delta and Omicron variants, among the human population is driven, among others, by mutations within the receptor-binding domain (RBD) of the S protein, which results in stronger binding to the receptor and subsequent increased hydrolysis of the furin and 3CLpro cleavage site that augment virus entry to the permissive cells. However, although more than 100 mutations have been described in the gene encoding the S protein, only some of them are associated with an increase in the pathogenicity of the virus. Particular attention is focused on mutations affecting the residues that form the stable interface with the ACE2 receptor; however, mutations lying outside the interaction region may also be of significance, triggering a change in the protein’s conformation [38].

Computational approaches have been applied to unravel the driving forces, at the atomic level, of the dominant mutations for SARS-CoV-2 variants that stabilize the protein S RBD-ACE2 complex. Based on the ΔG_bind_ calculation results and assuming a positive correlation between RBD-ACE2 affinity and virion transmissivity/infection, Ridgway et al. [38] extrapolated higher transmission rates (relative to the wild-type) for variants possessing one or more of the following substitutions: N501F, N501H, N501M, N501Y (Alpha, Omicron), E484Q, E484R (Delta, Kappa) or K417R, all of which exhibited gains in ACE2 binding affinity, i.e., negative ΔG_bind_ values. Moreover, the point mutation L452R (present in BA.5 Omicron) and its combination with T478K (present in Delta and Omicron) have a positive impact on the RBD-ACE2 binding affinity by increasing the flexibility of the RBD loop that interacts with ACE2. Arginine at positions 452, 484 and 501 of RBD, where mutations occur, stabilizes both the conformation of protein S, which facilitates molecular recognition with ACE2, and the RBD-ACE2 complex [38,39]. Moreover, molecular dynamics simulations and docking studies based on the structure of interacting viral and host proteins are enabling the development of new drugs with potential for COVID-19 treatment [40].

## 5. Conclusions

In this study, we present the alterations in circulating SARS-CoV-2 variants from September 2021 to 31 August 2022 in Southern Poland with reference to the remaining part of the country as well as neighboring countries. We observed the occurrence of new variants of the virus, among which those possessing mutations conferring increased replication efficiency and immune escape spread rapidly and quickly replaced the earlier variants. Tracking the emergence and spread of new SARS-CoV-2 variants is essential for providing information to make decisions on vaccine development and therapy, as well as predicting the course of infection with subsequent variants of the virus. What is more, the understanding of mutations and their impact on the stability of protein S structure may contribute to the development of prevention and treatment strategies for COVID-19.

## Data Availability

All of the SARS-CoV-2 nucleotide sequences obtained in the study have been deposited in GISAID and are available in Table S1.

## Supplementary Materials

The following supporting information can be downloaded at: https://www.mdpi.com/article/10.3390/pathogens14070708/s1, Table S1: The complete list of genomes used in phylogenetic analyses and deposited in GISAID.

## Abbreviations

The following abbreviations are used in this manuscript: SARS-CoV-2—Severe acute respiratory syndrome coronavirus 2; GISAID—Global Initiative on Sharing All Influenza Data; WGS—Whole-Genome Sequencing.
