# Supplementary material for: From Delta to Omicron—Genetic Epidemiology of SARS-CoV-2 (hCoV-19) in Southern Poland
Source: Pathogens. 2025 Jul 17;14(7):708. doi: 10.3390/pathogens14070708 (PMC12298073; doi:10.3390/pathogens14070708)
Supplement: Supplementary file 1 [file pathogens-14-00708-s001.zip › pathogens-3715870-supplementary.pdf]

Table S1. The complete list of genomes used in phylogenetic analyses.

EPI_ISL_6328149	EPI_ISL_6326539	EPI_ISL_6326519	EPI_ISL_9650873	EPI_ISL_9650871
EPI_ISL_9650869	EPI_ISL_9650865	EPI_ISL_9650860	EPI_ISL_9650840	EPI_ISL_9650826
EPI_ISL_9650823	EPI_ISL_9650818	EPI_ISL_9627252	EPI_ISL_9627247	EPI_ISL_9627242
EPI_ISL_9627233	EPI_ISL_9627229	EPI_ISL_9627212	EPI_ISL_9627207	EPI_ISL_9627206
EPI_ISL_9627175	EPI_ISL_9627174	EPI_ISL_9615908	EPI_ISL_9615903	EPI_ISL_9615900
EPI_ISL_9615897	EPI_ISL_9615895	EPI_ISL_9613402	EPI_ISL_9613386	EPI_ISL_9588646
EPI_ISL_9588644	EPI_ISL_9588642	EPI_ISL_9588631	EPI_ISL_9588612	EPI_ISL_9588597
EPI_ISL_9588591	EPI_ISL_9588585	EPI_ISL_9588583	EPI_ISL_9588582	EPI_ISL_9588577
EPI_ISL_9588576	EPI_ISL_9588574	EPI_ISL_9588569	EPI_ISL_8498146	EPI_ISL_8498131
EPI_ISL_8498110	EPI_ISL_8498014	EPI_ISL_9514801	EPI_ISL_9514795	EPI_ISL_9487521
EPI_ISL_9468292	EPI_ISL_9467328	EPI_ISL_9467300	EPI_ISL_9460233	EPI_ISL_9460211
EPI_ISL_9460205	EPI_ISL_9460202	EPI_ISL_9460201	EPI_ISL_9460164	EPI_ISL_6192173
EPI_ISL_9369363	EPI_ISL_9369361	EPI_ISL_9356022	EPI_ISL_9355968	EPI_ISL_9343171
EPI_ISL_9343154	EPI_ISL_9343054	EPI_ISL_9343053	EPI_ISL_9343033	EPI_ISL_9325690
EPI_ISL_9325680	EPI_ISL_9325676	EPI_ISL_9325657	EPI_ISL_9325631	EPI_ISL_9325621
EPI_ISL_9325604	EPI_ISL_9325599	EPI_ISL_9325596	EPI_ISL_9325583	EPI_ISL_9325575
EPI_ISL_9325200	EPI_ISL_9325177	EPI_ISL_9325168	EPI_ISL_9321878	EPI_ISL_8408387
EPI_ISL_8402469	EPI_ISL_8402455	EPI_ISL_8402450	EPI_ISL_8402432	EPI_ISL_8402400
EPI_ISL_8402344	EPI_ISL_8402304	EPI_ISL_8402270	EPI_ISL_8402257	EPI_ISL_8402249
EPI_ISL_9259373	EPI_ISL_8381314	EPI_ISL_9179641	EPI_ISL_9179602	EPI_ISL_8345109
EPI_ISL_8345105	EPI_ISL_8345098	EPI_ISL_8345081	EPI_ISL_8345072	EPI_ISL_8345070
EPI_ISL_8345066	EPI_ISL_9156764	EPI_ISL_9156748	EPI_ISL_9156745	EPI_ISL_9144765
EPI_ISL_9144755	EPI_ISL_9144731	EPI_ISL_9144715	EPI_ISL_9144706	EPI_ISL_9144661
EPI_ISL_9144328	EPI_ISL_9126378	EPI_ISL_9126360	EPI_ISL_9126339	EPI_ISL_6115415
EPI_ISL_8318030	EPI_ISL_8318026	EPI_ISL_8318008	EPI_ISL_8317995	EPI_ISL_8317967
EPI_ISL_8317960	EPI_ISL_8317946	EPI_ISL_8317939	EPI_ISL_8317915	EPI_ISL_8317892
EPI_ISL_8317873	EPI_ISL_8317570	EPI_ISL_8317558	EPI_ISL_8317556	EPI_ISL_8317550
EPI_ISL_8317547	EPI_ISL_8317538	EPI_ISL_8317531	EPI_ISL_8317530	EPI_ISL_8317519
EPI_ISL_8317518	EPI_ISL_8317502	EPI_ISL_8317488	EPI_ISL_8317430	EPI_ISL_8317399
EPI_ISL_8317374	EPI_ISL_9074355	EPI_ISL_9074313	EPI_ISL_9467306	EPI_ISL_9067514
EPI_ISL_9067505	EPI_ISL_8317920	EPI_ISL_8317928	EPI_ISL_8317289	EPI_ISL_8317344
EPI_ISL_8317351	EPI_ISL_8317357	EPI_ISL_8317350	EPI_ISL_8317339	EPI_ISL_8317307
EPI_ISL_8317306	EPI_ISL_8317314	EPI_ISL_8317422	EPI_ISL_8317431	EPI_ISL_8317520
EPI_ISL_9018667	EPI_ISL_9018655	EPI_ISL_9018617	EPI_ISL_9018589	EPI_ISL_9018582
EPI_ISL_9018546	EPI_ISL_9018531	EPI_ISL_9018478	EPI_ISL_9018476	EPI_ISL_9018473
EPI_ISL_9018470	EPI_ISL_9018454	EPI_ISL_9014348	EPI_ISL_9014285	EPI_ISL_9014278
EPI_ISL_8294474	EPI_ISL_8294456	EPI_ISL_8294453	EPI_ISL_8294433	EPI_ISL_8994037
EPI_ISL_8994036	EPI_ISL_8994033	EPI_ISL_8989294	EPI_ISL_8989240	EPI_ISL_8989228
EPI_ISL_6051148	EPI_ISL_9343110	EPI_ISL_8896577	EPI_ISL_8896565	EPI_ISL_6035784
EPI_ISL_8885040	EPI_ISL_8875838	EPI_ISL_8875814	EPI_ISL_8252966	EPI_ISL_8252121
EPI_ISL_8252090	EPI_ISL_8252062	EPI_ISL_8840454	EPI_ISL_8840442	EPI_ISL_8840422
EPI_ISL_8810749	EPI_ISL_8808547	EPI_ISL_8808522	EPI_ISL_8808484	EPI_ISL_8808479
EPI_ISL_6326333	EPI_ISL_8801906	EPI_ISL_8801762	EPI_ISL_8801739	EPI_ISL_8801731
EPI_ISL_8801729	EPI_ISL_8801723	EPI_ISL_8801714	EPI_ISL_8801711	EPI_ISL_8801680
EPI_ISL_8801668	EPI_ISL_8801666	EPI_ISL_8801623	EPI_ISL_8801592	EPI_ISL_8801550
EPI_ISL_8801520	EPI_ISL_8801515	EPI_ISL_8801510	EPI_ISL_8800951	EPI_ISL_8800948
EPI_ISL_8800913	EPI_ISL_8800872	EPI_ISL_8800798	EPI_ISL_8800723	EPI_ISL_8207486
EPI_ISL_6010339	EPI_ISL_8776549	EPI_ISL_8766374	EPI_ISL_8766366	EPI_ISL_8766354
EPI_ISL_8754112	EPI_ISL_8754084	EPI_ISL_8754076	EPI_ISL_8754060	EPI_ISL_8754057
EPI_ISL_8174074	EPI_ISL_8174062	EPI_ISL_8725387	EPI_ISL_8725338	EPI_ISL_8725326
EPI_ISL_8725277	EPI_ISL_8725264	EPI_ISL_8725251	EPI_ISL_8725217	EPI_ISL_8725204
EPI_ISL_8725189	EPI_ISL_8725153	EPI_ISL_8725152	EPI_ISL_8725146	EPI_ISL_8725019
EPI_ISL_8712886	EPI_ISL_8712884	EPI_ISL_8712881	EPI_ISL_8712828	EPI_ISL_8712827

EPI_ISL_8712822	EPI_ISL_8712816	EPI_ISL_8147512	EPI_ISL_8147482	EPI_ISL_8143534
EPI_ISL_8143530	EPI_ISL_8143528	EPI_ISL_8143524	EPI_ISL_8143511	EPI_ISL_8142465
EPI_ISL_8142463	EPI_ISL_8657575	EPI_ISL_8657566	EPI_ISL_8657551	EPI_ISL_8657548
EPI_ISL_8657547	EPI_ISL_8657537	EPI_ISL_8657529	EPI_ISL_8657526	EPI_ISL_8657512
EPI_ISL_8657483	EPI_ISL_8657425	EPI_ISL_8657420	EPI_ISL_8614415	EPI_ISL_5942404
EPI_ISL_5937563	EPI_ISL_8541477	EPI_ISL_8541461	EPI_ISL_8541458	EPI_ISL_8541407
EPI_ISL_8541324	EPI_ISL_8541298	EPI_ISL_8541232	EPI_ISL_8076533	EPI_ISL_8076520
EPI_ISL_8076490	EPI_ISL_8076475	EPI_ISL_8076453	EPI_ISL_8062130	EPI_ISL_8062101
EPI_ISL_8062067	EPI_ISL_8062041	EPI_ISL_8058817	EPI_ISL_8058803	EPI_ISL_8058791
EPI_ISL_8049295	EPI_ISL_8049274	EPI_ISL_5918594	EPI_ISL_8006705	EPI_ISL_8006701
EPI_ISL_7952020	EPI_ISL_7952000	EPI_ISL_7951981	EPI_ISL_7951978	EPI_ISL_7951921
EPI_ISL_7951916	EPI_ISL_7951907	EPI_ISL_7951905	EPI_ISL_7951898	EPI_ISL_7951874
EPI_ISL_7951868	EPI_ISL_7951858	EPI_ISL_7951854	EPI_ISL_7936423	EPI_ISL_7936397
EPI_ISL_7922831	EPI_ISL_7922822	EPI_ISL_7922814	EPI_ISL_7922797	EPI_ISL_7922508
EPI_ISL_7922491	EPI_ISL_7922466	EPI_ISL_7922432	EPI_ISL_7922417	EPI_ISL_7922413
EPI_ISL_7922412	EPI_ISL_7922409	EPI_ISL_7922374	EPI_ISL_7922366	EPI_ISL_7922365
EPI_ISL_7922333	EPI_ISL_7922303	EPI_ISL_7873012	EPI_ISL_7872994	EPI_ISL_7872989
EPI_ISL_7872980	EPI_ISL_7872962	EPI_ISL_7872948	EPI_ISL_7872930	EPI_ISL_7872918
EPI_ISL_7872889	EPI_ISL_7872872	EPI_ISL_7870458	EPI_ISL_7870445	EPI_ISL_7870440
EPI_ISL_7870434	EPI_ISL_7852382	EPI_ISL_7852380	EPI_ISL_7852378	EPI_ISL_7852373
EPI_ISL_7852363	EPI_ISL_7814208	EPI_ISL_7814203	EPI_ISL_7814195	EPI_ISL_7814177
EPI_ISL_7814169	EPI_ISL_7814160	EPI_ISL_7814134	EPI_ISL_7782272	EPI_ISL_7782263
EPI_ISL_7782261	EPI_ISL_7782234	EPI_ISL_7777613	EPI_ISL_9468334	EPI_ISL_7728494
EPI_ISL_7727935	EPI_ISL_7727138	EPI_ISL_7727113	EPI_ISL_7715519	EPI_ISL_7715482
EPI_ISL_7697661	EPI_ISL_7697654	EPI_ISL_7697638	EPI_ISL_7697619	EPI_ISL_7697607
EPI_ISL_7697602	EPI_ISL_7694550	EPI_ISL_7694520	EPI_ISL_9650835	EPI_ISL_7675424
EPI_ISL_7675423	EPI_ISL_7675410	EPI_ISL_7675406	EPI_ISL_7675405	EPI_ISL_8207501
EPI_ISL_7615066	EPI_ISL_7614977	EPI_ISL_7614969	EPI_ISL_7614956	EPI_ISL_7480245
EPI_ISL_7480161	EPI_ISL_7480049	EPI_ISL_7479949	EPI_ISL_7465914	EPI_ISL_7465867
EPI_ISL_7465705	EPI_ISL_8384069	EPI_ISL_7429775	EPI_ISL_7429552	EPI_ISL_7429389
EPI_ISL_7429214	EPI_ISL_7429153	EPI_ISL_7428742	EPI_ISL_7428459	EPI_ISL_7428420
EPI_ISL_7360662	EPI_ISL_7360655	EPI_ISL_7360095	EPI_ISL_7285870	EPI_ISL_7285868
EPI_ISL_7285865	EPI_ISL_7285819	EPI_ISL_7285770	EPI_ISL_7220155	EPI_ISL_7220124
EPI_ISL_7220002	EPI_ISL_7219997	EPI_ISL_7219929	EPI_ISL_7219916	EPI_ISL_7219899
EPI_ISL_7219886	EPI_ISL_7219781	EPI_ISL_7219754	EPI_ISL_7219748	EPI_ISL_7219597
EPI_ISL_7219578	EPI_ISL_7218536	EPI_ISL_7212281	EPI_ISL_7212259	EPI_ISL_7212249
EPI_ISL_7212237	EPI_ISL_7212230	EPI_ISL_8402289	EPI_ISL_8402251	EPI_ISL_8402350
EPI_ISL_8402427	EPI_ISL_9144342	EPI_ISL_7127673	EPI_ISL_7127190	EPI_ISL_7126632
EPI_ISL_8402298	EPI_ISL_6965494	EPI_ISL_8402323	EPI_ISL_8402300	EPI_ISL_8402306
EPI_ISL_6826314	EPI_ISL_9650844	EPI_ISL_8402451	EPI_ISL_8402467	EPI_ISL_8402408
EPI_ISL_8402406	EPI_ISL_5781679	EPI_ISL_9325645	EPI_ISL_6886543	EPI_ISL_6878153
EPI_ISL_6878132	EPI_ISL_6832209	EPI_ISL_6826362	EPI_ISL_6826304	EPI_ISL_6815985
EPI_ISL_6701499	EPI_ISL_6701483	EPI_ISL_6701473	EPI_ISL_6640429	EPI_ISL_6640388
EPI_ISL_6640360	EPI_ISL_6603885	EPI_ISL_6603793	EPI_ISL_8062040	EPI_ISL_6507704
EPI_ISL_6491334	EPI_ISL_6491317	EPI_ISL_6471969	EPI_ISL_6471894	EPI_ISL_6471879
EPI_ISL_6471862	EPI_ISL_6471842	EPI_ISL_6437648	EPI_ISL_6437608	EPI_ISL_6204361
EPI_ISL_8317453	EPI_ISL_9325554	EPI_ISL_5564666	EPI_ISL_5541230	EPI_ISL_5541179
EPI_ISL_8384062	EPI_ISL_9321863	EPI_ISL_8384063	EPI_ISL_5316743	EPI_ISL_8384064
EPI_ISL_9343165	EPI_ISL_5194586	EPI_ISL_7102356	EPI_ISL_5159415	EPI_ISL_7101664
EPI_ISL_6326252	EPI_ISL_6326009	EPI_ISL_5065256	EPI_ISL_9325674	EPI_ISL_7126842
EPI_ISL_9018481	EPI_ISL_4512565	EPI_ISL_9613383	EPI_ISL_6437669	EPI_ISL_4274929
EPI_ISL_5563743	EPI_ISL_6471877	EPI_ISL_6471955	EPI_ISL_5564752	EPI_ISL_6491322
EPI_ISL_6491325	EPI_ISL_6491328	EPI_ISL_6514493	EPI_ISL_8481015	EPI_ISL_8481018
EPI_ISL_8480980	EPI_ISL_8480944	EPI_ISL_8480948	EPI_ISL_7127645	EPI_ISL_8498097
EPI_ISL_8498056	EPI_ISL_8498037	EPI_ISL_8498160	EPI_ISL_8498142	EPI_ISL_8498136

EPI_ISL_7219501	EPI_ISL_7219803	EPI_ISL_7219986	EPI_ISL_8541478	EPI_ISL_7219449
EPI_ISL_7285726	EPI_ISL_7285755	EPI_ISL_7285891	EPI_ISL_7285856	EPI_ISL_7285872
EPI_ISL_6640394	EPI_ISL_6640392	EPI_ISL_6640363	EPI_ISL_8657445	EPI_ISL_7359986
EPI_ISL_7360126	EPI_ISL_6640426	EPI_ISL_7364703	EPI_ISL_8657515	EPI_ISL_7951974
EPI_ISL_8712841	EPI_ISL_8712865	EPI_ISL_8712888	EPI_ISL_7480334	EPI_ISL_7480063
EPI_ISL_7480093	EPI_ISL_8725344	EPI_ISL_6701497	EPI_ISL_8725388	EPI_ISL_6640290
EPI_ISL_8725124	EPI_ISL_8725197	EPI_ISL_7429263	EPI_ISL_7429025	EPI_ISL_7429521
EPI_ISL_7428988	EPI_ISL_5835617	EPI_ISL_8766403	EPI_ISL_8801794	EPI_ISL_8801475
EPI_ISL_8801487	EPI_ISL_8801632	EPI_ISL_8801648	EPI_ISL_8801641	EPI_ISL_8801548
EPI_ISL_8800819	EPI_ISL_8800826	EPI_ISL_8800845	EPI_ISL_8800960	EPI_ISL_8801925
EPI_ISL_8801929	EPI_ISL_8800716	EPI_ISL_8800735	EPI_ISL_8808595	EPI_ISL_5834886
EPI_ISL_8810751	EPI_ISL_8810733	EPI_ISL_7465848	EPI_ISL_8318003	EPI_ISL_5852408
EPI_ISL_7615108	EPI_ISL_7614914	EPI_ISL_7615070	EPI_ISL_7615003	EPI_ISL_7615006
EPI_ISL_8402404	EPI_ISL_8062105	EPI_ISL_7480397	EPI_ISL_8840434	EPI_ISL_7480032
EPI_ISL_7697719	EPI_ISL_7873007	EPI_ISL_7715512	EPI_ISL_7731617	EPI_ISL_7479909
EPI_ISL_7727041	EPI_ISL_9325610	EPI_ISL_7782279	EPI_ISL_7777664	EPI_ISL_7777670
EPI_ISL_5884423	EPI_ISL_5884456	EPI_ISL_7814162	EPI_ISL_8208112	EPI_ISL_8317950
EPI_ISL_7951913	EPI_ISL_8840432	EPI_ISL_7936419	EPI_ISL_8480955	EPI_ISL_7212319
EPI_ISL_8058821	EPI_ISL_8317959	EPI_ISL_8207459	EPI_ISL_8402459	EPI_ISL_8896560
EPI_ISL_7615012	EPI_ISL_7615056	EPI_ISL_7951861	EPI_ISL_7952022	EPI_ISL_9067438
EPI_ISL_8006663	EPI_ISL_9325672	EPI_ISL_7872951	EPI_ISL_8076456	EPI_ISL_7615011
EPI_ISL_7614954	EPI_ISL_9074326	EPI_ISL_8402422	EPI_ISL_5065223	EPI_ISL_8318032
EPI_ISL_8294483	EPI_ISL_9083175	EPI_ISL_8317901	EPI_ISL_7870462	EPI_ISL_7872875
EPI_ISL_8317584	EPI_ISL_8207463	EPI_ISL_6471873	EPI_ISL_8776543	EPI_ISL_8712821
EPI_ISL_8317864	EPI_ISL_7951964	EPI_ISL_8657485	EPI_ISL_9144767	EPI_ISL_9144719
EPI_ISL_9144742	EPI_ISL_7872876	EPI_ISL_6014316	EPI_ISL_7675451	EPI_ISL_8801917
EPI_ISL_9156744	EPI_ISL_7727015	EPI_ISL_7729851	EPI_ISL_8317386	EPI_ISL_7852377
EPI_ISL_8402371	EPI_ISL_7922377	EPI_ISL_8884986	EPI_ISL_8294486	EPI_ISL_8801650
EPI_ISL_7952024	EPI_ISL_7101865	EPI_ISL_8317551	EPI_ISL_7697721	EPI_ISL_7697704
EPI_ISL_7697666	EPI_ISL_7697577	EPI_ISL_7697714	EPI_ISL_7697690	EPI_ISL_8498116
EPI_ISL_7951890	EPI_ISL_7922345	EPI_ISL_8006699	EPI_ISL_6051255	EPI_ISL_6051307
EPI_ISL_9083187	EPI_ISL_8801787	EPI_ISL_8317387	EPI_ISL_8317925	EPI_ISL_8480929
EPI_ISL_8402430	EPI_ISL_8252054	EPI_ISL_8147479	EPI_ISL_8725379	EPI_ISL_8143487
EPI_ISL_8317988	EPI_ISL_8294488	EPI_ISL_7715501	EPI_ISL_7715494	EPI_ISL_7715508
EPI_ISL_8800788	EPI_ISL_7872988	EPI_ISL_8062046	EPI_ISL_8174051	EPI_ISL_7727109
EPI_ISL_7727071	EPI_ISL_8252049	EPI_ISL_7729738	EPI_ISL_7729657	EPI_ISL_6121498
EPI_ISL_7936390	EPI_ISL_8207490	EPI_ISL_7731476	EPI_ISL_7731449	EPI_ISL_7731593
EPI_ISL_8317390	EPI_ISL_7873031	EPI_ISL_8174037	EPI_ISL_8294437	EPI_ISL_7922291
EPI_ISL_7936391	EPI_ISL_8143491	EPI_ISL_8317891	EPI_ISL_8657437	EPI_ISL_8884991
EPI_ISL_7870464	EPI_ISL_7782226	EPI_ISL_7782264	EPI_ISL_7782296	EPI_ISL_9325592
EPI_ISL_7782246	EPI_ISL_7777653	EPI_ISL_7777611	EPI_ISL_8498030	EPI_ISL_6192256
EPI_ISL_6192583	EPI_ISL_9249737	EPI_ISL_8174042	EPI_ISL_8317964	EPI_ISL_8061993
EPI_ISL_8251793	EPI_ISL_8480993	EPI_ISL_9259377	EPI_ISL_9259369	EPI_ISL_5942455
EPI_ISL_7782287	EPI_ISL_7782254	EPI_ISL_7219867	EPI_ISL_7814182	EPI_ISL_7814166
EPI_ISL_7814174	EPI_ISL_7814141	EPI_ISL_7814150	EPI_ISL_7951947	EPI_ISL_7814140
EPI_ISL_8801594	EPI_ISL_8801694	EPI_ISL_8317994	EPI_ISL_8657494	EPI_ISL_8345094
EPI_ISL_6325925	EPI_ISL_7951895	EPI_ISL_6326001	EPI_ISL_6326407	EPI_ISL_6325928
EPI_ISL_6326533	EPI_ISL_6326556	EPI_ISL_6328310	EPI_ISL_6328361	EPI_ISL_6328148
EPI_ISL_6328303	EPI_ISL_8143495	EPI_ISL_7429046	EPI_ISL_8840466	EPI_ISL_8294496
EPI_ISL_8294479	EPI_ISL_8208096	EPI_ISL_8207196	EPI_ISL_9462996	EPI_ISL_8541256
EPI_ISL_7968997	EPI_ISL_9144697	EPI_ISL_7922398	EPI_ISL_8252050	EPI_ISL_8006707
EPI_ISL_8317487	EPI_ISL_8317568	EPI_ISL_9343185	EPI_ISL_9325576	EPI_ISL_9325587
EPI_ISL_8541364	EPI_ISL_9018658	EPI_ISL_9325123	EPI_ISL_9325125	EPI_ISL_9325136
EPI_ISL_9321861	EPI_ISL_9321881	EPI_ISL_9325593	EPI_ISL_9325659	EPI_ISL_9325617
EPI_ISL_9325603	EPI_ISL_9325601	EPI_ISL_9325614	EPI_ISL_8840467	EPI_ISL_9325581

EPI_ISL_9343149	EPI_ISL_9325662	EPI_ISL_9325667	EPI_ISL_9325679	EPI_ISL_9325648
EPI_ISL_9325649	EPI_ISL_9325651	EPI_ISL_9325652	EPI_ISL_9325629	EPI_ISL_9325683
EPI_ISL_9325687	EPI_ISL_9325688	EPI_ISL_9325694	EPI_ISL_9343013	EPI_ISL_9344298
EPI_ISL_9344263	EPI_ISL_9344229	EPI_ISL_9343217	EPI_ISL_9343207	EPI_ISL_9343198
EPI_ISL_9343176	EPI_ISL_9343168	EPI_ISL_9343150	EPI_ISL_9343155	EPI_ISL_9343137
EPI_ISL_9343138	EPI_ISL_9343117	EPI_ISL_9343118	EPI_ISL_9343126	EPI_ISL_9343127
EPI_ISL_9343124	EPI_ISL_9343106	EPI_ISL_9343116	EPI_ISL_9343104	EPI_ISL_9343080
EPI_ISL_9343087	EPI_ISL_9343079	EPI_ISL_9343069	EPI_ISL_9343051	EPI_ISL_9343057
EPI_ISL_9343029	EPI_ISL_9343037	EPI_ISL_9343024	EPI_ISL_9343027	EPI_ISL_9342997
EPI_ISL_9355967	EPI_ISL_9355964	EPI_ISL_8498198	EPI_ISL_9356031	EPI_ISL_9356030
EPI_ISL_9356032	EPI_ISL_9356016	EPI_ISL_9356018	EPI_ISL_8657461	EPI_ISL_9156796
EPI_ISL_9369367	EPI_ISL_9343097	EPI_ISL_8801698	EPI_ISL_9014336	EPI_ISL_8754050
EPI_ISL_8725169	EPI_ISL_8541369	EPI_ISL_8317869	EPI_ISL_7872870	EPI_ISL_7872970
EPI_ISL_9460228	EPI_ISL_9460220	EPI_ISL_9460216	EPI_ISL_8541371	EPI_ISL_9467319
EPI_ISL_9462997	EPI_ISL_9144744	EPI_ISL_8840472	EPI_ISL_9514803	EPI_ISL_9521137
EPI_ISL_9521128	EPI_ISL_9521126	EPI_ISL_9521146	EPI_ISL_9521142	EPI_ISL_9521163
EPI_ISL_8207473	EPI_ISL_8896573	EPI_ISL_9144675	EPI_ISL_7360646	EPI_ISL_9613395
EPI_ISL_9613391	EPI_ISL_9613390	EPI_ISL_9613388	EPI_ISL_9613381	EPI_ISL_9615905
EPI_ISL_9627209	EPI_ISL_9627197	EPI_ISL_9627258	EPI_ISL_9627256	EPI_ISL_9627236
EPI_ISL_9650816	EPI_ISL_9650815	EPI_ISL_9650822	EPI_ISL_8058876	EPI_ISL_9467276
EPI_ISL_8317576	EPI_ISL_7465737	EPI_ISL_7465599	EPI_ISL_7465636	EPI_ISL_7465640
EPI_ISL_7465812	EPI_ISL_7465925	EPI_ISL_7465512	EPI_ISL_7465763	EPI_ISL_7465797
EPI_ISL_8317871	EPI_ISL_8498041	EPI_ISL_7614980	EPI_ISL_7614948	EPI_ISL_7614972
EPI_ISL_9144690	EPI_ISL_9083191	EPI_ISL_9463015	EPI_ISL_6774870	EPI_ISL_6774852
EPI_ISL_8896568	EPI_ISL_6812374	EPI_ISL_7715515	EPI_ISL_8108828	EPI_ISL_8108830
EPI_ISL_7782294	EPI_ISL_6827393	EPI_ISL_6827415	EPI_ISL_6827478	EPI_ISL_6326004
EPI_ISL_6827459	EPI_ISL_6826313	EPI_ISL_6826335	EPI_ISL_6826374	EPI_ISL_7359848
EPI_ISL_6832211	EPI_ISL_6832207	EPI_ISL_6832228	EPI_ISL_8142405	EPI_ISL_8142412
EPI_ISL_8147513	EPI_ISL_8801678	EPI_ISL_8062115	EPI_ISL_6846376	EPI_ISL_8989277
EPI_ISL_8498078	EPI_ISL_8174047	EPI_ISL_8174068	EPI_ISL_4882613	EPI_ISL_6852182
EPI_ISL_9018594	EPI_ISL_9344294	EPI_ISL_4892794	EPI_ISL_4892798	EPI_ISL_4892768
EPI_ISL_8207506	EPI_ISL_8207212	EPI_ISL_6862002	EPI_ISL_8207460	EPI_ISL_8208099
EPI_ISL_8208094	EPI_ISL_8208123	EPI_ISL_8207189	EPI_ISL_6878157	EPI_ISL_6878148
EPI_ISL_6878124	EPI_ISL_6878172	EPI_ISL_6878164	EPI_ISL_6878166	EPI_ISL_6883982
EPI_ISL_6886514	EPI_ISL_6886506	EPI_ISL_9126379	EPI_ISL_8252048	EPI_ISL_8252093
EPI_ISL_8252965	EPI_ISL_8251769	EPI_ISL_8251781	EPI_ISL_8251744	EPI_ISL_8251801
EPI_ISL_6326008	EPI_ISL_8252985	EPI_ISL_7922379	EPI_ISL_9144679	EPI_ISL_8402333
EPI_ISL_8810735	EPI_ISL_9014280	EPI_ISL_8317882	EPI_ISL_8142466	EPI_ISL_8801599
EPI_ISL_8345083	EPI_ISL_6965456	EPI_ISL_6965473	EPI_ISL_7127633	EPI_ISL_8317963
EPI_ISL_8840438	EPI_ISL_8657444	EPI_ISL_8657573	EPI_ISL_8657572	EPI_ISL_8657549
EPI_ISL_8657556	EPI_ISL_7922465	EPI_ISL_7922361	EPI_ISL_7922348	EPI_ISL_8498070
EPI_ISL_7872971	EPI_ISL_7922472	EPI_ISL_8801713	EPI_ISL_8480954	EPI_ISL_9663707
EPI_ISL_9663686	EPI_ISL_9663678	EPI_ISL_9663672	EPI_ISL_9663667	EPI_ISL_9663655
EPI_ISL_9663639	EPI_ISL_9663618	EPI_ISL_9663352	EPI_ISL_9663328	EPI_ISL_9663310
EPI_ISL_9662937	EPI_ISL_9662923	EPI_ISL_9662919	EPI_ISL_9658984	EPI_ISL_9658961
EPI_ISL_9658933	EPI_ISL_9658932	EPI_ISL_9658930	EPI_ISL_9658927	EPI_ISL_9658926
EPI_ISL_9658922	EPI_ISL_9658919	EPI_ISL_9658917	EPI_ISL_9658913	EPI_ISL_9658912
EPI_ISL_9658907	EPI_ISL_9658897	EPI_ISL_9658893	EPI_ISL_9658892	EPI_ISL_9658880
EPI_ISL_9658870	EPI_ISL_9658858	EPI_ISL_9658838	EPI_ISL_9658227	EPI_ISL_9650891
EPI_ISL_9650888	EPI_ISL_9650886	EPI_ISL_9650884	EPI_ISL_9650882	EPI_ISL_9650880
EPI_ISL_9650876	EPI_ISL_10449818	EPI_ISL_9845129	EPI_ISL_9845070	EPI_ISL_9845074
EPI_ISL_10501700	EPI_ISL_9844703	EPI_ISL_9855467	EPI_ISL_9855456	EPI_ISL_9855449
EPI_ISL_9878159	EPI_ISL_9878156	EPI_ISL_9878104	EPI_ISL_9878111	EPI_ISL_9878125
EPI_ISL_10316803	EPI_ISL_10316804	EPI_ISL_9900023	EPI_ISL_9900016	EPI_ISL_9900018
EPI_ISL_9900063	EPI_ISL_9900062	EPI_ISL_9900056	EPI_ISL_9906321	EPI_ISL_9906323

EPI_ISL_10125308	EPI_ISL_10501708	EPI_ISL_9753108	EPI_ISL_10590609	EPI_ISL_9753110
EPI_ISL_9955193	EPI_ISL_9955213	EPI_ISL_9955232	EPI_ISL_9955299	EPI_ISL_9955280
EPI_ISL_9753111	EPI_ISL_9793611	EPI_ISL_9954853	EPI_ISL_9963903	EPI_ISL_9963916
EPI_ISL_9968299	EPI_ISL_9968316	EPI_ISL_9968346	EPI_ISL_9968350	EPI_ISL_9968404
EPI_ISL_9963893	EPI_ISL_9963882	EPI_ISL_9963921	EPI_ISL_9981530	EPI_ISL_10316813
EPI_ISL_10132613	EPI_ISL_9845115	EPI_ISL_9845123	EPI_ISL_10069217	EPI_ISL_10069306
EPI_ISL_10069331	EPI_ISL_10069332	EPI_ISL_9662933	EPI_ISL_9751134	EPI_ISL_10205624
EPI_ISL_10205659	EPI_ISL_10205674	EPI_ISL_10441842	EPI_ISL_10250627	EPI_ISL_10250630
EPI_ISL_10250600	EPI_ISL_10250616	EPI_ISL_10250618	EPI_ISL_9662943	EPI_ISL_10205644
EPI_ISL_10590644	EPI_ISL_9663650	EPI_ISL_10316595	EPI_ISL_10132850	EPI_ISL_9844650
EPI_ISL_10316798	EPI_ISL_10316799	EPI_ISL_10316840	EPI_ISL_10316843	EPI_ISL_10316814
EPI_ISL_10316815	EPI_ISL_10316832	EPI_ISL_10316808	EPI_ISL_10590651	EPI_ISL_10132651
EPI_ISL_9663353	EPI_ISL_10348754	EPI_ISL_10341605	EPI_ISL_10341610	EPI_ISL_10249356
EPI_ISL_10249359	EPI_ISL_10132661	EPI_ISL_10205661	EPI_ISL_9845225	EPI_ISL_10069264
EPI_ISL_10316565	EPI_ISL_10069345	EPI_ISL_10069344	EPI_ISL_10069336	EPI_ISL_10069334
EPI_ISL_10069330	EPI_ISL_10069327	EPI_ISL_10069323	EPI_ISL_10069318	EPI_ISL_10069313
EPI_ISL_10069298	EPI_ISL_10069295	EPI_ISL_10069292	EPI_ISL_10069288	EPI_ISL_10069279
EPI_ISL_10069278	EPI_ISL_10069275	EPI_ISL_10069267	EPI_ISL_10069263	EPI_ISL_10069247
EPI_ISL_10069245	EPI_ISL_10069236	EPI_ISL_10069227	EPI_ISL_10069222	EPI_ISL_10069221
EPI_ISL_10069220	EPI_ISL_10069216	EPI_ISL_10069215	EPI_ISL_10069212	EPI_ISL_10069210
EPI_ISL_10069198	EPI_ISL_10069072	EPI_ISL_10069068	EPI_ISL_10069044	EPI_ISL_10069043
EPI_ISL_10069272	EPI_ISL_10316572	EPI_ISL_10590572	EPI_ISL_9878169	EPI_ISL_9878165
EPI_ISL_9878150	EPI_ISL_9878138	EPI_ISL_9878132	EPI_ISL_9878131	EPI_ISL_9878130
EPI_ISL_9878129	EPI_ISL_9878121	EPI_ISL_9878116	EPI_ISL_9878113	EPI_ISL_9878110
EPI_ISL_9878107	EPI_ISL_9878106	EPI_ISL_9878105	EPI_ISL_9878103	EPI_ISL_9878102
EPI_ISL_9878096	EPI_ISL_9878092	EPI_ISL_9878090	EPI_ISL_9878089	EPI_ISL_9878088
EPI_ISL_9878086	EPI_ISL_9845302	EPI_ISL_9845300	EPI_ISL_9845291	EPI_ISL_9845289
EPI_ISL_9845288	EPI_ISL_9845287	EPI_ISL_9845286	EPI_ISL_9845284	EPI_ISL_9845271
EPI_ISL_9845266	EPI_ISL_9845263	EPI_ISL_9845259	EPI_ISL_9845252	EPI_ISL_9845237
EPI_ISL_9845231	EPI_ISL_9845227	EPI_ISL_9845226	EPI_ISL_9845211	EPI_ISL_9845206
EPI_ISL_9845203	EPI_ISL_9845177	EPI_ISL_9845173	EPI_ISL_9845170	EPI_ISL_9845168
EPI_ISL_9845151	EPI_ISL_9845149	EPI_ISL_9845148	EPI_ISL_9845137	EPI_ISL_9845125
EPI_ISL_9845121	EPI_ISL_9845120	EPI_ISL_9845100	EPI_ISL_9845051	EPI_ISL_9845032
EPI_ISL_9845029	EPI_ISL_9845023	EPI_ISL_9845015	EPI_ISL_9845014	EPI_ISL_9845007
EPI_ISL_9845004	EPI_ISL_9844998	EPI_ISL_9844995	EPI_ISL_9844731	EPI_ISL_9844724
EPI_ISL_9844723	EPI_ISL_9844720	EPI_ISL_9844717	EPI_ISL_9844714	EPI_ISL_9844707
EPI_ISL_9844701	EPI_ISL_9844700	EPI_ISL_9844696	EPI_ISL_9844692	EPI_ISL_9844680
EPI_ISL_9844677	EPI_ISL_9844672	EPI_ISL_9844670	EPI_ISL_9844667	EPI_ISL_9844666
EPI_ISL_9844651	EPI_ISL_9844649	EPI_ISL_10069074	EPI_ISL_10316574	EPI_ISL_10441844
EPI_ISL_9658877	EPI_ISL_10449968	EPI_ISL_10449955	EPI_ISL_10450041	EPI_ISL_10450017
EPI_ISL_10316578	EPI_ISL_10316879	EPI_ISL_10316881	EPI_ISL_10069248	EPI_ISL_10069060
EPI_ISL_10069239	EPI_ISL_10316583	EPI_ISL_10590583	EPI_ISL_9844683	EPI_ISL_10578624
EPI_ISL_10069228	EPI_ISL_9823286	EPI_ISL_9823278	EPI_ISL_9968341	EPI_ISL_9968448
EPI_ISL_9968290	EPI_ISL_10069071	EPI_ISL_9968292	EPI_ISL_9968392	EPI_ISL_9968345
EPI_ISL_10250594	EPI_ISL_9750763	EPI_ISL_9650885	EPI_ISL_9750764	EPI_ISL_10341554
EPI_ISL_9753087	EPI_ISL_9658866	EPI_ISL_10348752	EPI_ISL_10316787	EPI_ISL_9844988
EPI_ISL_10249288	EPI_ISL_10590579	EPI_ISL_9750768	EPI_ISL_9658903	EPI_ISL_10127289
EPI_ISL_10127081	EPI_ISL_10450081	EPI_ISL_10127290	EPI_ISL_9855455	EPI_ISL_10069070
EPI_ISL_9658958	EPI_ISL_10125392	EPI_ISL_10127092	EPI_ISL_10341592	EPI_ISL_10450055
EPI_ISL_10127293	EPI_ISL_10132693	EPI_ISL_9963894	EPI_ISL_10069283	EPI_ISL_10316775
EPI_ISL_9663656	EPI_ISL_9845156	EPI_ISL_10316796	EPI_ISL_10436825	EPI_ISL_10132659
EPI_ISL_10253079	EPI_ISL_9793664	EPI_ISL_10069252	EPI_ISL_9753157	EPI_ISL_10441857
EPI_ISL_10450052	EPI_ISL_9845184	EPI_ISL_9793663	EPI_ISL_10590560	EPI_ISL_10590570
EPI_ISL_10590565	EPI_ISL_10132678	EPI_ISL_10436857	EPI_ISL_10450099	EPI_ISL_9751066
EPI_ISL_9900025	EPI_ISL_10449926	EPI_ISL_10249289	EPI_ISL_9878127	EPI_ISL_10125390

EPI_ISL_10132627	EPI_ISL_10205690	EPI_ISL_10253058	EPI_ISL_9751154	EPI_ISL_9650898
EPI_ISL_9963880	EPI_ISL_9658841	EPI_ISL_9658847	EPI_ISL_9658869	EPI_ISL_9658915
EPI_ISL_9658942	EPI_ISL_9658939	EPI_ISL_9658979	EPI_ISL_9663347	EPI_ISL_9663357
EPI_ISL_9663349	EPI_ISL_9662941	EPI_ISL_9663680	EPI_ISL_9663695	EPI_ISL_9663696
EPI_ISL_9663622	EPI_ISL_9663634	EPI_ISL_9663630	EPI_ISL_9663629	EPI_ISL_9663644
EPI_ISL_9663652	EPI_ISL_9663646	EPI_ISL_9844665	EPI_ISL_9650893	EPI_ISL_9900045
EPI_ISL_10132660	EPI_ISL_10069297	EPI_ISL_9751160	EPI_ISL_9751041	EPI_ISL_9751036
EPI_ISL_9753124	EPI_ISL_9753102	EPI_ISL_9753162	EPI_ISL_9753144	EPI_ISL_9753156
EPI_ISL_9751079	EPI_ISL_9751078	EPI_ISL_10590622	EPI_ISL_9650892	EPI_ISL_10125427
EPI_ISL_10069230	EPI_ISL_10450122	EPI_ISL_10450119	EPI_ISL_10450106	EPI_ISL_10450105
EPI_ISL_10450094	EPI_ISL_10450093	EPI_ISL_10450090	EPI_ISL_10450087	EPI_ISL_10450085
EPI_ISL_10450084	EPI_ISL_10450078	EPI_ISL_10450069	EPI_ISL_10450068	EPI_ISL_10450066
EPI_ISL_10450063	EPI_ISL_10450054	EPI_ISL_10450050	EPI_ISL_10450047	EPI_ISL_10450046
EPI_ISL_10450045	EPI_ISL_10450043	EPI_ISL_10450042	EPI_ISL_10450039	EPI_ISL_10450037
EPI_ISL_10450036	EPI_ISL_10450024	EPI_ISL_10450018	EPI_ISL_10450015	EPI_ISL_10450014
EPI_ISL_10450011	EPI_ISL_10450006	EPI_ISL_10450001	EPI_ISL_10449993	EPI_ISL_10449990
EPI_ISL_10449989	EPI_ISL_10449988	EPI_ISL_10449982	EPI_ISL_10449977	EPI_ISL_10449976
EPI_ISL_10449969	EPI_ISL_10449964	EPI_ISL_10449962	EPI_ISL_10449957	EPI_ISL_10449956
EPI_ISL_10449952	EPI_ISL_10449950	EPI_ISL_10449946	EPI_ISL_10449940	EPI_ISL_10449933
EPI_ISL_10449928	EPI_ISL_10449927	EPI_ISL_10449924	EPI_ISL_10449921	EPI_ISL_10449920
EPI_ISL_10449918	EPI_ISL_10449916	EPI_ISL_10449912	EPI_ISL_10449911	EPI_ISL_10449909
EPI_ISL_10449904	EPI_ISL_10449902	EPI_ISL_10449892	EPI_ISL_10449891	EPI_ISL_10449890
EPI_ISL_10449889	EPI_ISL_10449887	EPI_ISL_10449885	EPI_ISL_10449883	EPI_ISL_10449867
EPI_ISL_10449860	EPI_ISL_10449857	EPI_ISL_10449856	EPI_ISL_10449854	EPI_ISL_10449847
EPI_ISL_10449844	EPI_ISL_10449842	EPI_ISL_10449838	EPI_ISL_10449837	EPI_ISL_10449835
EPI_ISL_10449834	EPI_ISL_10449833	EPI_ISL_10449824	EPI_ISL_10449821	EPI_ISL_10449820
EPI_ISL_10449813	EPI_ISL_10449808	EPI_ISL_10449798	EPI_ISL_10449795	EPI_ISL_10449787
EPI_ISL_10449785	EPI_ISL_10449784	EPI_ISL_10449782	EPI_ISL_10448266	EPI_ISL_10448264
EPI_ISL_10448256	EPI_ISL_10448254	EPI_ISL_10448249	EPI_ISL_10448248	EPI_ISL_10448246
EPI_ISL_10448235	EPI_ISL_10448229	EPI_ISL_10448228	EPI_ISL_10448223	EPI_ISL_10448209
EPI_ISL_10448206	EPI_ISL_10448186	EPI_ISL_10448185	EPI_ISL_10448179	EPI_ISL_10448155
EPI_ISL_10448143	EPI_ISL_10448139	EPI_ISL_9793684	EPI_ISL_9793682	EPI_ISL_9793650
EPI_ISL_9793631	EPI_ISL_9793626	EPI_ISL_9793627	EPI_ISL_9793622	EPI_ISL_9793605
EPI_ISL_9793596	EPI_ISL_9793597	EPI_ISL_9793595	EPI_ISL_10448172	EPI_ISL_10441862
EPI_ISL_10441860	EPI_ISL_10441859	EPI_ISL_10441855	EPI_ISL_10441851	EPI_ISL_10441848
EPI_ISL_10441843	EPI_ISL_10441828	EPI_ISL_10441827	EPI_ISL_10441824	EPI_ISL_10441819
EPI_ISL_10441818	EPI_ISL_10441815	EPI_ISL_10440805	EPI_ISL_10440802	EPI_ISL_10440794
EPI_ISL_10440792	EPI_ISL_10440773	EPI_ISL_10440771	EPI_ISL_10440769	EPI_ISL_10440767
EPI_ISL_10253080	EPI_ISL_10449827	EPI_ISL_10102029	EPI_ISL_10436885	EPI_ISL_10436878
EPI_ISL_10436867	EPI_ISL_10436859	EPI_ISL_10436849	EPI_ISL_10436844	EPI_ISL_10436824
EPI_ISL_10542430	EPI_ISL_9845131	EPI_ISL_11008768	EPI_ISL_11008767	EPI_ISL_11008761
EPI_ISL_11008755	EPI_ISL_11008729	EPI_ISL_11008727	EPI_ISL_11008724	EPI_ISL_11008723
EPI_ISL_11008713	EPI_ISL_11008704	EPI_ISL_11004905	EPI_ISL_11004902	EPI_ISL_11004899
EPI_ISL_11004896	EPI_ISL_11004894	EPI_ISL_11004884	EPI_ISL_11004861	EPI_ISL_11004859
EPI_ISL_10993779	EPI_ISL_10993740	EPI_ISL_10993713	EPI_ISL_10993711	EPI_ISL_10993708
EPI_ISL_10993707	EPI_ISL_10993680	EPI_ISL_10993679	EPI_ISL_10993658	EPI_ISL_10993656
EPI_ISL_10993655	EPI_ISL_10987671	EPI_ISL_10981371	EPI_ISL_10981367	EPI_ISL_10981366
EPI_ISL_10981362	EPI_ISL_10981293	EPI_ISL_10981292	EPI_ISL_10929389	EPI_ISL_10929384
EPI_ISL_10929364	EPI_ISL_10929358	EPI_ISL_10929352	EPI_ISL_10929338	EPI_ISL_10878403
EPI_ISL_10829949	EPI_ISL_10829945	EPI_ISL_10829943	EPI_ISL_10829942	EPI_ISL_10829938
EPI_ISL_10829936	EPI_ISL_10829934	EPI_ISL_10829930	EPI_ISL_10829927	EPI_ISL_10829912
EPI_ISL_10829911	EPI_ISL_10829910	EPI_ISL_10829904	EPI_ISL_10829903	EPI_ISL_10829893
EPI_ISL_10829892	EPI_ISL_10829890	EPI_ISL_10829884	EPI_ISL_10829883	EPI_ISL_10829878
EPI_ISL_10829876	EPI_ISL_10829874	EPI_ISL_10829871	EPI_ISL_10829868	EPI_ISL_10829867
EPI_ISL_10816721	EPI_ISL_10816719	EPI_ISL_10816716	EPI_ISL_10816714	EPI_ISL_10816712

EPI_ISL_10816710	EPI_ISL_10816705	EPI_ISL_10816702	EPI_ISL_10816701	EPI_ISL_10816699
EPI_ISL_10810620	EPI_ISL_10805376	EPI_ISL_10805331	EPI_ISL_10803991	EPI_ISL_10803977
EPI_ISL_10803976	EPI_ISL_10803971	EPI_ISL_10782923	EPI_ISL_10782920	EPI_ISL_10782916
EPI_ISL_10782913	EPI_ISL_10782911	EPI_ISL_10765329	EPI_ISL_10765328	EPI_ISL_10765326
EPI_ISL_10765308	EPI_ISL_10765293	EPI_ISL_10765286	EPI_ISL_10765232	EPI_ISL_10765223
EPI_ISL_10765218	EPI_ISL_10765214	EPI_ISL_10765193	EPI_ISL_10765191	EPI_ISL_10754165
EPI_ISL_10754138	EPI_ISL_10754136	EPI_ISL_10754133	EPI_ISL_10754131	EPI_ISL_10754094
EPI_ISL_10754091	EPI_ISL_10754085	EPI_ISL_10754081	EPI_ISL_10754032	EPI_ISL_10754026
EPI_ISL_10754021	EPI_ISL_10754012	EPI_ISL_10754009	EPI_ISL_10754008	EPI_ISL_10753999
EPI_ISL_10750690	EPI_ISL_10750682	EPI_ISL_10750680	EPI_ISL_10750678	EPI_ISL_10750676
EPI_ISL_10750669	EPI_ISL_10750668	EPI_ISL_10750653	EPI_ISL_10750640	EPI_ISL_10750612
EPI_ISL_10750611	EPI_ISL_10703895	EPI_ISL_10703894	EPI_ISL_10703884	EPI_ISL_10703877
EPI_ISL_10703868	EPI_ISL_10703860	EPI_ISL_10703858	EPI_ISL_10703847	EPI_ISL_10703841
EPI_ISL_10703839	EPI_ISL_10703836	EPI_ISL_10703826	EPI_ISL_10676406	EPI_ISL_10676403
EPI_ISL_10676385	EPI_ISL_10676382	EPI_ISL_10676380	EPI_ISL_10676371	EPI_ISL_10676370
EPI_ISL_10676369	EPI_ISL_10676352	EPI_ISL_10631359	EPI_ISL_10631354	EPI_ISL_10631331
EPI_ISL_10631328	EPI_ISL_10631289	EPI_ISL_10631288	EPI_ISL_10590657	EPI_ISL_10590654
EPI_ISL_10590648	EPI_ISL_10590628	EPI_ISL_10590615	EPI_ISL_10590613	EPI_ISL_10590610
EPI_ISL_10590607	EPI_ISL_10590604	EPI_ISL_10590602	EPI_ISL_10590567	EPI_ISL_10590561
EPI_ISL_10590549	EPI_ISL_10590546	EPI_ISL_10590544	EPI_ISL_10590537	EPI_ISL_10590533
EPI_ISL_10590532	EPI_ISL_10590529	EPI_ISL_10590528	EPI_ISL_10590517	EPI_ISL_10590516
EPI_ISL_10590515	EPI_ISL_10590509	EPI_ISL_10590505	EPI_ISL_10590497	EPI_ISL_10578623
EPI_ISL_10542470	EPI_ISL_10542469	EPI_ISL_10542464	EPI_ISL_10542462	EPI_ISL_10542456
EPI_ISL_10542454	EPI_ISL_10542448	EPI_ISL_10542434	EPI_ISL_10542433	EPI_ISL_10542409
EPI_ISL_10542390	EPI_ISL_10542386	EPI_ISL_10542380	EPI_ISL_10505983	EPI_ISL_10505980
EPI_ISL_10505975	EPI_ISL_10505972	EPI_ISL_10505965	EPI_ISL_10501735	EPI_ISL_10501721
EPI_ISL_10501711	EPI_ISL_10348762	EPI_ISL_10348746	EPI_ISL_10348740	EPI_ISL_10348739
EPI_ISL_10341611	EPI_ISL_10341585	EPI_ISL_10341548	EPI_ISL_10341542	EPI_ISL_10316903
EPI_ISL_10316899	EPI_ISL_10316891	EPI_ISL_10316889	EPI_ISL_10316886	EPI_ISL_10316873
EPI_ISL_10316847	EPI_ISL_10316844	EPI_ISL_10316835	EPI_ISL_10316823	EPI_ISL_10316822
EPI_ISL_10316821	EPI_ISL_10316806	EPI_ISL_10316802	EPI_ISL_10316800	EPI_ISL_10316794
EPI_ISL_10316598	EPI_ISL_10316596	EPI_ISL_10316592	EPI_ISL_10316589	EPI_ISL_10316577
EPI_ISL_10316576	EPI_ISL_10316563	EPI_ISL_10316561	EPI_ISL_10297275	EPI_ISL_10253119
EPI_ISL_10253118	EPI_ISL_10253117	EPI_ISL_10253099	EPI_ISL_10253061	EPI_ISL_10250628
EPI_ISL_10250623	EPI_ISL_10250605	EPI_ISL_10249354	EPI_ISL_10249343	EPI_ISL_10249335
EPI_ISL_10249320	EPI_ISL_10249314	EPI_ISL_10249307	EPI_ISL_10249303	EPI_ISL_10227338
EPI_ISL_10205683	EPI_ISL_10205650	EPI_ISL_10205648	EPI_ISL_10205637	EPI_ISL_10205636
EPI_ISL_10205632	EPI_ISL_10205631	EPI_ISL_10205621	EPI_ISL_10205620	EPI_ISL_10205618
EPI_ISL_10205614	EPI_ISL_10132849	EPI_ISL_10132691	EPI_ISL_10132683	EPI_ISL_10132679
EPI_ISL_10132672	EPI_ISL_10132670	EPI_ISL_10132657	EPI_ISL_10132655	EPI_ISL_10132649
EPI_ISL_10132647	EPI_ISL_10132646	EPI_ISL_10132644	EPI_ISL_10132643	EPI_ISL_10132633
EPI_ISL_10132629	EPI_ISL_10132624	EPI_ISL_10132623	EPI_ISL_10132621	EPI_ISL_10132620
EPI_ISL_10127287	EPI_ISL_10127281	EPI_ISL_10127262	EPI_ISL_10127108	EPI_ISL_10127107
EPI_ISL_10127094	EPI_ISL_10127091	EPI_ISL_10127082	EPI_ISL_10127078	EPI_ISL_10127073
EPI_ISL_10127072	EPI_ISL_10127068	EPI_ISL_10127063	EPI_ISL_10125422	EPI_ISL_10125415
EPI_ISL_10125409	EPI_ISL_10125408	EPI_ISL_10125407	EPI_ISL_10125402	EPI_ISL_10125385
EPI_ISL_10125383	EPI_ISL_10125374	EPI_ISL_10125371	EPI_ISL_10125369	EPI_ISL_10125355
EPI_ISL_10125346	EPI_ISL_10125344	EPI_ISL_10125343	EPI_ISL_10125342	EPI_ISL_10125335
EPI_ISL_10125312	EPI_ISL_10125306	EPI_ISL_10125301	EPI_ISL_10125293	EPI_ISL_10125275
EPI_ISL_10125272	EPI_ISL_10102043	EPI_ISL_10102030	EPI_ISL_10102027	EPI_ISL_10102024
EPI_ISL_10102009	EPI_ISL_10102006	EPI_ISL_10102003	EPI_ISL_10101999	EPI_ISL_10101993
EPI_ISL_10101990	EPI_ISL_10101989	EPI_ISL_10101984	EPI_ISL_10101974	EPI_ISL_10101973
EPI_ISL_10101970	EPI_ISL_10101969	EPI_ISL_10101968	EPI_ISL_10101966	EPI_ISL_9981580
EPI_ISL_9981578	EPI_ISL_9981577	EPI_ISL_9981573	EPI_ISL_9981569	EPI_ISL_9981565
EPI_ISL_9981560	EPI_ISL_9981552	EPI_ISL_9981548	EPI_ISL_9981547	EPI_ISL_9981545

EPI_ISL_9981544	EPI_ISL_9981539	EPI_ISL_9981537	EPI_ISL_9981534	EPI_ISL_9981532
EPI_ISL_9981524	EPI_ISL_9981523	EPI_ISL_9981522	EPI_ISL_9981520	EPI_ISL_9968440
EPI_ISL_9968438	EPI_ISL_9968352	EPI_ISL_9968331	EPI_ISL_9968315	EPI_ISL_9968313
EPI_ISL_9968309	EPI_ISL_9963924	EPI_ISL_9963920	EPI_ISL_9963919	EPI_ISL_9963917
EPI_ISL_9963914	EPI_ISL_9963905	EPI_ISL_9963899	EPI_ISL_9963897	EPI_ISL_9963877
EPI_ISL_9955296	EPI_ISL_9955295	EPI_ISL_9955288	EPI_ISL_9955270	EPI_ISL_9955252
EPI_ISL_9955250	EPI_ISL_9955230	EPI_ISL_9955227	EPI_ISL_9955223	EPI_ISL_9955210
EPI_ISL_9955180	EPI_ISL_9954921	EPI_ISL_9954894	EPI_ISL_9954842	EPI_ISL_9954841
EPI_ISL_9954834	EPI_ISL_9914593	EPI_ISL_9914592	EPI_ISL_9900065	EPI_ISL_9900052
EPI_ISL_9900048	EPI_ISL_9900046	EPI_ISL_9900044	EPI_ISL_9900041	EPI_ISL_9900040
EPI_ISL_9900038	EPI_ISL_9900035	EPI_ISL_9900024	EPI_ISL_9900012	EPI_ISL_9900008
EPI_ISL_9900007	EPI_ISL_9899996	EPI_ISL_9899995	EPI_ISL_9899994	EPI_ISL_9899988
EPI_ISL_9855474	EPI_ISL_9855461	EPI_ISL_9855460	EPI_ISL_9855436	EPI_ISL_9823322
EPI_ISL_9823319	EPI_ISL_9823290	EPI_ISL_9823274	EPI_ISL_9823265	EPI_ISL_9823251
EPI_ISL_9793679	EPI_ISL_9793671	EPI_ISL_9793669	EPI_ISL_9793668	EPI_ISL_9793659
EPI_ISL_9793657	EPI_ISL_9793656	EPI_ISL_9793654	EPI_ISL_9793646	EPI_ISL_9793634
EPI_ISL_9793621	EPI_ISL_9793618	EPI_ISL_9793615	EPI_ISL_9793591	EPI_ISL_9753165
EPI_ISL_9753164	EPI_ISL_9753153	EPI_ISL_9753143	EPI_ISL_9753141	EPI_ISL_9753139
EPI_ISL_9753125	EPI_ISL_9753122	EPI_ISL_9753121	EPI_ISL_9753120	EPI_ISL_9753119
EPI_ISL_9753112	EPI_ISL_9753088	EPI_ISL_9753085	EPI_ISL_9753084	EPI_ISL_9751175
EPI_ISL_9751140	EPI_ISL_9751133	EPI_ISL_9751131	EPI_ISL_9751126	EPI_ISL_9751123
EPI_ISL_9751122	EPI_ISL_9751118	EPI_ISL_9751113	EPI_ISL_9751108	EPI_ISL_9751105
EPI_ISL_9751104	EPI_ISL_9751103	EPI_ISL_9751101	EPI_ISL_9751093	EPI_ISL_9751087
EPI_ISL_9751082	EPI_ISL_9751069	EPI_ISL_9751064	EPI_ISL_9751056	EPI_ISL_9751053
EPI_ISL_9751052	EPI_ISL_9751047	EPI_ISL_9751037	EPI_ISL_9751035	EPI_ISL_9751034
EPI_ISL_9751032	EPI_ISL_9750784	EPI_ISL_9750781	EPI_ISL_9750779	EPI_ISL_9750773
EPI_ISL_9750767	EPI_ISL_9750760	EPI_ISL_9663704	EPI_ISL_11008770	EPI_ISL_11008775
EPI_ISL_11008777	EPI_ISL_11008780	EPI_ISL_11008782	EPI_ISL_11008784	EPI_ISL_11008786
EPI_ISL_11008793	EPI_ISL_11008812	EPI_ISL_11008813	EPI_ISL_11008820	EPI_ISL_11008822
EPI_ISL_11008823	EPI_ISL_11008834	EPI_ISL_11008855	EPI_ISL_11008861	EPI_ISL_11008876
EPI_ISL_11008881	EPI_ISL_11008891	EPI_ISL_11008903	EPI_ISL_11008911	EPI_ISL_11008932
EPI_ISL_11008958	EPI_ISL_11008959	EPI_ISL_11008991	EPI_ISL_11008993	EPI_ISL_11009001
EPI_ISL_11009002	EPI_ISL_11009012	EPI_ISL_11009384	EPI_ISL_11011999	EPI_ISL_11021099
EPI_ISL_11021112	EPI_ISL_11021116	EPI_ISL_11021120	EPI_ISL_11021149	EPI_ISL_11021166
EPI_ISL_11021175	EPI_ISL_11021180	EPI_ISL_11021875	EPI_ISL_11021883	EPI_ISL_11021912
EPI_ISL_11021917	EPI_ISL_11058725	EPI_ISL_11075719	EPI_ISL_11075720	EPI_ISL_11075737
EPI_ISL_11075786	EPI_ISL_11075800	EPI_ISL_11109245	EPI_ISL_11109274	EPI_ISL_11124896
EPI_ISL_11124903	EPI_ISL_11124906	EPI_ISL_11159919	EPI_ISL_11159939	EPI_ISL_11159959
EPI_ISL_11162878	EPI_ISL_11162892	EPI_ISL_11162907	EPI_ISL_11162948	EPI_ISL_11162956
EPI_ISL_11217529	EPI_ISL_11217530	EPI_ISL_11217539	EPI_ISL_11217550	EPI_ISL_11217554
EPI_ISL_11217557	EPI_ISL_11217567	EPI_ISL_11217572	EPI_ISL_11217584	EPI_ISL_11217585
EPI_ISL_11265926	EPI_ISL_11265935	EPI_ISL_11265942	EPI_ISL_11265961	EPI_ISL_11265971
EPI_ISL_11327023	EPI_ISL_11327024	EPI_ISL_11327041	EPI_ISL_11343265	EPI_ISL_11343271
EPI_ISL_11343303	EPI_ISL_11343309	EPI_ISL_11343334	EPI_ISL_11343363	EPI_ISL_11343371
EPI_ISL_11343387	EPI_ISL_11343394	EPI_ISL_11343396	EPI_ISL_11343400	EPI_ISL_11343429
EPI_ISL_11343433	EPI_ISL_11343436	EPI_ISL_11343444	EPI_ISL_11343448	EPI_ISL_11343451
EPI_ISL_11343471	EPI_ISL_11343502	EPI_ISL_11343510	EPI_ISL_11343530	EPI_ISL_11343540
EPI_ISL_11343542	EPI_ISL_11343551	EPI_ISL_11343555	EPI_ISL_11349883	EPI_ISL_11349885
EPI_ISL_11354347	EPI_ISL_11354633	EPI_ISL_11354640	EPI_ISL_11354646	EPI_ISL_11378215
EPI_ISL_11378220	EPI_ISL_11378226	EPI_ISL_11378227	EPI_ISL_11378242	EPI_ISL_11378243
EPI_ISL_11378256	EPI_ISL_11414096	EPI_ISL_11414104	EPI_ISL_11414113	EPI_ISL_11414129
EPI_ISL_11414149	EPI_ISL_11414161	EPI_ISL_11414162	EPI_ISL_11458510	EPI_ISL_11458513
EPI_ISL_11458536	EPI_ISL_11458546	EPI_ISL_11458566	EPI_ISL_11462643	EPI_ISL_11462662
EPI_ISL_11462673	EPI_ISL_11462684	EPI_ISL_11462713	EPI_ISL_11462714	EPI_ISL_11462723
EPI_ISL_11464565	EPI_ISL_11464577	EPI_ISL_11464578	EPI_ISL_11464602	EPI_ISL_11464624

EPI_ISL_11464638	EPI_ISL_11464644	EPI_ISL_11464648	EPI_ISL_11464666	EPI_ISL_11478925
EPI_ISL_11478951	EPI_ISL_11478955	EPI_ISL_11484523	EPI_ISL_11520393	EPI_ISL_11520396
EPI_ISL_11520398	EPI_ISL_11520402	EPI_ISL_11520423	EPI_ISL_11520437	EPI_ISL_11520463
EPI_ISL_11520469	EPI_ISL_11520471	EPI_ISL_11520474	EPI_ISL_11525392	EPI_ISL_11525414
EPI_ISL_11525415	EPI_ISL_11525434	EPI_ISL_11525437	EPI_ISL_11525449	EPI_ISL_11564243
EPI_ISL_11672459	EPI_ISL_11672465	EPI_ISL_11672470	EPI_ISL_11672475	EPI_ISL_11672480
EPI_ISL_11672508	EPI_ISL_11679508	EPI_ISL_11679518	EPI_ISL_11679540	EPI_ISL_11679543
EPI_ISL_11679551	EPI_ISL_11679568	EPI_ISL_11761113	EPI_ISL_11761144	EPI_ISL_11761180
EPI_ISL_11761181	EPI_ISL_11761226	EPI_ISL_11761248	EPI_ISL_11761259	EPI_ISL_11761267
EPI_ISL_11761276	EPI_ISL_11761280	EPI_ISL_11779974	EPI_ISL_11779983	EPI_ISL_11779998
EPI_ISL_11780001	EPI_ISL_11780028	EPI_ISL_11780077	EPI_ISL_11780083	EPI_ISL_11810760
EPI_ISL_11810768	EPI_ISL_11828440	EPI_ISL_11828446	EPI_ISL_11828454	EPI_ISL_11828455
EPI_ISL_11837557	EPI_ISL_11837573	EPI_ISL_11837577	EPI_ISL_11837601	EPI_ISL_11837602
EPI_ISL_11837621	EPI_ISL_11837624	EPI_ISL_11837628	EPI_ISL_11872244	EPI_ISL_11872246
EPI_ISL_11872269	EPI_ISL_11872344	EPI_ISL_11872353	EPI_ISL_11872375	EPI_ISL_11878515
EPI_ISL_11878516	EPI_ISL_11905949	EPI_ISL_11905950	EPI_ISL_11905962	EPI_ISL_11905970
EPI_ISL_11923520	EPI_ISL_11923549	EPI_ISL_11923576	EPI_ISL_11932461	EPI_ISL_11932481
EPI_ISL_11932505	EPI_ISL_11932510	EPI_ISL_11932512	EPI_ISL_11932516	EPI_ISL_11932518
EPI_ISL_11932544	EPI_ISL_11932552	EPI_ISL_11932578	EPI_ISL_11935884	EPI_ISL_11935898
EPI_ISL_11935928	EPI_ISL_11935932	EPI_ISL_11935935	EPI_ISL_11935955	EPI_ISL_11935964
EPI_ISL_11935978	EPI_ISL_11997418	EPI_ISL_11997426	EPI_ISL_11997430	EPI_ISL_11997432
EPI_ISL_11997434	EPI_ISL_11997465	EPI_ISL_11997467	EPI_ISL_11997499	EPI_ISL_11997562
EPI_ISL_11997580	EPI_ISL_11997589	EPI_ISL_11997590	EPI_ISL_11997934	EPI_ISL_11998138
EPI_ISL_11998155	EPI_ISL_11998160	EPI_ISL_11998163	EPI_ISL_11998179	EPI_ISL_11998209
EPI_ISL_12008320	EPI_ISL_12008337	EPI_ISL_12008428	EPI_ISL_12008598	EPI_ISL_12008601
EPI_ISL_12008604	EPI_ISL_12010120	EPI_ISL_12010146	EPI_ISL_12010150	EPI_ISL_12010172
EPI_ISL_12010179	EPI_ISL_12089868	EPI_ISL_12089906	EPI_ISL_12089932	EPI_ISL_12101538
EPI_ISL_12132553	EPI_ISL_12132597	EPI_ISL_12132608	EPI_ISL_12132619	EPI_ISL_12132631
EPI_ISL_12132632	EPI_ISL_12132654	EPI_ISL_12139627	EPI_ISL_12139703	EPI_ISL_12139707
EPI_ISL_12139715	EPI_ISL_12139745	EPI_ISL_12139751	EPI_ISL_12139754	EPI_ISL_12139770
EPI_ISL_12139773	EPI_ISL_12139788	EPI_ISL_12144164	EPI_ISL_12154950	EPI_ISL_12154967
EPI_ISL_12156557	EPI_ISL_12156579	EPI_ISL_12156586	EPI_ISL_12156596	EPI_ISL_12156631
EPI_ISL_12156634	EPI_ISL_12156644	EPI_ISL_12156654	EPI_ISL_12156682	EPI_ISL_12156684
EPI_ISL_12156893	EPI_ISL_12156900	EPI_ISL_12156926	EPI_ISL_12156930	EPI_ISL_12156946
EPI_ISL_12156965	EPI_ISL_12156976	EPI_ISL_12157002	EPI_ISL_12157010	EPI_ISL_12157399
EPI_ISL_12178969	EPI_ISL_12178973	EPI_ISL_12179505	EPI_ISL_12179512	EPI_ISL_12179513
EPI_ISL_12179554	EPI_ISL_12179558	EPI_ISL_12179570	EPI_ISL_12223112	EPI_ISL_12223143
EPI_ISL_12252355	EPI_ISL_12254157	EPI_ISL_12254561	EPI_ISL_12254579	EPI_ISL_12254590
EPI_ISL_12254598	EPI_ISL_12254619	EPI_ISL_12254620	EPI_ISL_12256746	EPI_ISL_12256776
EPI_ISL_12261481	EPI_ISL_12261500	EPI_ISL_12285318	EPI_ISL_12285356	EPI_ISL_12285359
EPI_ISL_12285379	EPI_ISL_12319564	EPI_ISL_12323376	EPI_ISL_12323392	EPI_ISL_12323400
EPI_ISL_12323405	EPI_ISL_12323411	EPI_ISL_12323414	EPI_ISL_12400753	EPI_ISL_12400772
EPI_ISL_12400788	EPI_ISL_12400800	EPI_ISL_12400810	EPI_ISL_12400811	EPI_ISL_12400856
EPI_ISL_12400879	EPI_ISL_12400913	EPI_ISL_12400923	EPI_ISL_12400934	EPI_ISL_12400938
EPI_ISL_12400940	EPI_ISL_12400957	EPI_ISL_12400961	EPI_ISL_12400963	EPI_ISL_12429797
EPI_ISL_12429830	EPI_ISL_12429834	EPI_ISL_12429841	EPI_ISL_12475717	EPI_ISL_12475809
EPI_ISL_12475810	EPI_ISL_12475840	EPI_ISL_12476504	EPI_ISL_12476517	EPI_ISL_12476522
EPI_ISL_12476561	EPI_ISL_12486977	EPI_ISL_12486987	EPI_ISL_12487006	EPI_ISL_12487020
EPI_ISL_12487071	EPI_ISL_12487072	EPI_ISL_12487093	EPI_ISL_12487113	EPI_ISL_12487117
EPI_ISL_12487129	EPI_ISL_12487137	EPI_ISL_12487198	EPI_ISL_12487237	EPI_ISL_12487244
EPI_ISL_12487288	EPI_ISL_12567664	EPI_ISL_12582920	EPI_ISL_12582953	EPI_ISL_12582990
EPI_ISL_12583007	EPI_ISL_12583020	EPI_ISL_12583072	EPI_ISL_12586103	EPI_ISL_12586115
EPI_ISL_12590833	EPI_ISL_12590857	EPI_ISL_12590888	EPI_ISL_12590920	EPI_ISL_12610780
EPI_ISL_12613879	EPI_ISL_12613884	EPI_ISL_12613917	EPI_ISL_12613950	EPI_ISL_12640115
EPI_ISL_12640127	EPI_ISL_12640202	EPI_ISL_12640206	EPI_ISL_12640238	EPI_ISL_12686296

EPI_ISL_12689657	EPI_ISL_12706094	EPI_ISL_12706106	EPI_ISL_12757515	EPI_ISL_12785579
EPI_ISL_12804176	EPI_ISL_12861948	EPI_ISL_12861962	EPI_ISL_12861994	EPI_ISL_12862032
EPI_ISL_12903210	EPI_ISL_12903218	EPI_ISL_12952821	EPI_ISL_12952834	EPI_ISL_12952865
EPI_ISL_12952896	EPI_ISL_12952915	EPI_ISL_12952939	EPI_ISL_12952962	EPI_ISL_12952978
EPI_ISL_12952997	EPI_ISL_13028014	EPI_ISL_13028015	EPI_ISL_13028017	EPI_ISL_13028029
EPI_ISL_13028048	EPI_ISL_13028075	EPI_ISL_13034828	EPI_ISL_13037391	EPI_ISL_13037531
EPI_ISL_13037630	EPI_ISL_13065505	EPI_ISL_13158505	EPI_ISL_13183265	EPI_ISL_13202497
EPI_ISL_13202706	EPI_ISL_13202722	EPI_ISL_13202727	EPI_ISL_13202738	EPI_ISL_13202756
EPI_ISL_13228298	EPI_ISL_13228306	EPI_ISL_13228318	EPI_ISL_13251726	EPI_ISL_13251744
EPI_ISL_13251761	EPI_ISL_13251769	EPI_ISL_13251797	EPI_ISL_13285261	EPI_ISL_13285275
EPI_ISL_13285291	EPI_ISL_13285292	EPI_ISL_13285306	EPI_ISL_13285308	EPI_ISL_13372010
EPI_ISL_13372049	EPI_ISL_13391372	EPI_ISL_13619362	EPI_ISL_13619367	EPI_ISL_13692308
EPI_ISL_13744362	EPI_ISL_13744371	EPI_ISL_13744712	EPI_ISL_13755373	EPI_ISL_13755375
EPI_ISL_13756483	EPI_ISL_13756503	EPI_ISL_13799285	EPI_ISL_13869053	EPI_ISL_13869104
EPI_ISL_13884244	EPI_ISL_13906805	EPI_ISL_13952331	EPI_ISL_13961866	EPI_ISL_14026152
EPI_ISL_14026155	EPI_ISL_14026195	EPI_ISL_14026226	EPI_ISL_14026229	EPI_ISL_14026230
EPI_ISL_14026255	EPI_ISL_14026260	EPI_ISL_14026739	EPI_ISL_14026780	EPI_ISL_14096126
EPI_ISL_14174913	EPI_ISL_14174918	EPI_ISL_14174954	EPI_ISL_14175029	EPI_ISL_14197057
EPI_ISL_14197066	EPI_ISL_14199653	EPI_ISL_14201176	EPI_ISL_14218619	EPI_ISL_14238050
EPI_ISL_14238058	EPI_ISL_14238060	EPI_ISL_14238086	EPI_ISL_14238087	EPI_ISL_14240261
EPI_ISL_14240264	EPI_ISL_14240347	EPI_ISL_14240357	EPI_ISL_14259784	EPI_ISL_14259792
EPI_ISL_14315432	EPI_ISL_14356827	EPI_ISL_14356853	EPI_ISL_14362382	EPI_ISL_14362427
EPI_ISL_14362502	EPI_ISL_14440079	EPI_ISL_14440105	EPI_ISL_14440698	EPI_ISL_14440734
EPI_ISL_14440764	EPI_ISL_14441508	EPI_ISL_14441512	EPI_ISL_14441522	EPI_ISL_14475812
EPI_ISL_14475825	EPI_ISL_14475849	EPI_ISL_14475875	EPI_ISL_14524290	EPI_ISL_14524328
EPI_ISL_14524336	EPI_ISL_14590640	EPI_ISL_14590671	EPI_ISL_14590676	EPI_ISL_14590690
EPI_ISL_14590695	EPI_ISL_14590700	EPI_ISL_14604110	EPI_ISL_14604171	EPI_ISL_14604208
EPI_ISL_14604215	EPI_ISL_14604226	EPI_ISL_14604254	EPI_ISL_14604267	EPI_ISL_14659969
EPI_ISL_14660020	EPI_ISL_14660043	EPI_ISL_14701329	EPI_ISL_14701339	EPI_ISL_14701368
EPI_ISL_14701379	EPI_ISL_14766144	EPI_ISL_14766179	EPI_ISL_14766182	EPI_ISL_14766189
EPI_ISL_14768807	EPI_ISL_14768865	EPI_ISL_14768891	EPI_ISL_14768905	EPI_ISL_14768917
EPI_ISL_14768976	EPI_ISL_14768980	EPI_ISL_14769016	EPI_ISL_14769031	EPI_ISL_14770550
EPI_ISL_14778247	EPI_ISL_14778275	EPI_ISL_14778276	EPI_ISL_14778291	EPI_ISL_14815266
EPI_ISL_14815283	EPI_ISL_14815290	EPI_ISL_14815320	EPI_ISL_14815322	EPI_ISL_14841387
EPI_ISL_14841394	EPI_ISL_14841426	EPI_ISL_14841445	EPI_ISL_14846557	EPI_ISL_14846582
EPI_ISL_14846602	EPI_ISL_14864397	EPI_ISL_14864451	EPI_ISL_14888433	EPI_ISL_14888447
EPI_ISL_14888484	EPI_ISL_14888524	EPI_ISL_14888526	EPI_ISL_14888565	EPI_ISL_14892969
EPI_ISL_14892990	EPI_ISL_14893027	EPI_ISL_14893038	EPI_ISL_14893039	EPI_ISL_14893047
EPI_ISL_14893075	EPI_ISL_14893078	EPI_ISL_14893080	EPI_ISL_14920388	EPI_ISL_14920399
EPI_ISL_14922152	EPI_ISL_14922166	EPI_ISL_14934903	EPI_ISL_14946131	EPI_ISL_14946176
EPI_ISL_15021450	EPI_ISL_15021453	EPI_ISL_15022651	EPI_ISL_15022709	EPI_ISL_15022728
EPI_ISL_15023882	EPI_ISL_15023883	EPI_ISL_15023893	EPI_ISL_15023925	EPI_ISL_15038294
EPI_ISL_15038344	EPI_ISL_15038362	EPI_ISL_15038381	EPI_ISL_15045864	EPI_ISL_15045871
EPI_ISL_15058395	EPI_ISL_15058433	EPI_ISL_15058449	EPI_ISL_15074557	EPI_ISL_15074596
EPI_ISL_15139572	EPI_ISL_15139589	EPI_ISL_15139597	EPI_ISL_15139598	EPI_ISL_15139611
EPI_ISL_15139651	EPI_ISL_15139662	EPI_ISL_15139681	EPI_ISL_15139710	EPI_ISL_15139713
EPI_ISL_15139734	EPI_ISL_15169420	EPI_ISL_15169422	EPI_ISL_15296700	EPI_ISL_15296748
EPI_ISL_15296782	EPI_ISL_15367867	EPI_ISL_15367897	EPI_ISL_15494031	EPI_ISL_15856254
EPI_ISL_16128216	EPI_ISL_16128232	EPI_ISL_6324640	EPI_ISL_6774131	EPI_ISL_6774183
EPI_ISL_6774258	EPI_ISL_6774308	EPI_ISL_6774334	EPI_ISL_6774366	EPI_ISL_6774411
EPI_ISL_6774455	EPI_ISL_6774456	EPI_ISL_6774468	EPI_ISL_6774476	EPI_ISL_6774512
EPI_ISL_6774600	EPI_ISL_6774576	EPI_ISL_6774630	EPI_ISL_6774686	EPI_ISL_6774929
EPI_ISL_6775049	EPI_ISL_6775061	EPI_ISL_6775150	EPI_ISL_6775169	EPI_ISL_6775269
EPI_ISL_6810697	EPI_ISL_6858522	EPI_ISL_6858515	EPI_ISL_6858518	EPI_ISL_6858569
EPI_ISL_6944705	EPI_ISL_6944719	EPI_ISL_6944733	EPI_ISL_6944752	EPI_ISL_6944796

EPI_ISL_6944799	EPI_ISL_6944807	EPI_ISL_6944814	EPI_ISL_6944818	EPI_ISL_6944855
EPI_ISL_6944844	EPI_ISL_6944847	EPI_ISL_6944884	EPI_ISL_6944885	EPI_ISL_6944886
EPI_ISL_6944900	EPI_ISL_6944903	EPI_ISL_6944918	EPI_ISL_6944927	EPI_ISL_6944928
EPI_ISL_6944938	EPI_ISL_6944941	EPI_ISL_6944951	EPI_ISL_6944954	EPI_ISL_6944960
EPI_ISL_6944961	EPI_ISL_6945024	EPI_ISL_6945019	EPI_ISL_8420939	EPI_ISL_8420937
EPI_ISL_8420906	EPI_ISL_7141827	EPI_ISL_7141832	EPI_ISL_7141843	EPI_ISL_7141880
EPI_ISL_7141879	EPI_ISL_7141907	EPI_ISL_7141886	EPI_ISL_7141906	EPI_ISL_8379667
EPI_ISL_8377333	EPI_ISL_8377330	EPI_ISL_8377276	EPI_ISL_8377272	EPI_ISL_8377128
EPI_ISL_8377117	EPI_ISL_8377098	EPI_ISL_8377090	EPI_ISL_8351340	EPI_ISL_8345162
EPI_ISL_8345149	EPI_ISL_8345143	EPI_ISL_8338720	EPI_ISL_8338716	EPI_ISL_8338684
EPI_ISL_8338656	EPI_ISL_8338629	EPI_ISL_6110450	EPI_ISL_6110147	EPI_ISL_8315127
EPI_ISL_7367171	EPI_ISL_7367172	EPI_ISL_7367175	EPI_ISL_7367235	EPI_ISL_7367814
EPI_ISL_7367815	EPI_ISL_7367829	EPI_ISL_7367830	EPI_ISL_7367842	EPI_ISL_7367849
EPI_ISL_7367851	EPI_ISL_7367873	EPI_ISL_7367880	EPI_ISL_7367947	EPI_ISL_7367953
EPI_ISL_7367957	EPI_ISL_7368373	EPI_ISL_7368377	EPI_ISL_7368380	EPI_ISL_7368394
EPI_ISL_7368402	EPI_ISL_7368403	EPI_ISL_7368405	EPI_ISL_7368451	EPI_ISL_7368465
EPI_ISL_7368466	EPI_ISL_8306924	EPI_ISL_8306912	EPI_ISL_8306903	EPI_ISL_8294553
EPI_ISL_8294541	EPI_ISL_8286638	EPI_ISL_8286634	EPI_ISL_8285479	EPI_ISL_7622898
EPI_ISL_7622911	EPI_ISL_7622915	EPI_ISL_7622934	EPI_ISL_7622960	EPI_ISL_7622976
EPI_ISL_7622989	EPI_ISL_7623004	EPI_ISL_7623021	EPI_ISL_7623023	EPI_ISL_7623042
EPI_ISL_4633068	EPI_ISL_7731769	EPI_ISL_6012992	EPI_ISL_7835391	EPI_ISL_7835425
EPI_ISL_7836611	EPI_ISL_7836639	EPI_ISL_7836708	EPI_ISL_7836709	EPI_ISL_7836751
EPI_ISL_7836739	EPI_ISL_7836785	EPI_ISL_7836896	EPI_ISL_7836909	EPI_ISL_7836915
EPI_ISL_7836930	EPI_ISL_7836949	EPI_ISL_7837016	EPI_ISL_7886009	EPI_ISL_7886035
EPI_ISL_7886043	EPI_ISL_7886115	EPI_ISL_8188649	EPI_ISL_8188637	EPI_ISL_8173074
EPI_ISL_8173065	EPI_ISL_6931749	EPI_ISL_8165738	EPI_ISL_8164982	EPI_ISL_7924613
EPI_ISL_7924607	EPI_ISL_7924620	EPI_ISL_7924622	EPI_ISL_7924626	EPI_ISL_7924643
EPI_ISL_7924671	EPI_ISL_8152622	EPI_ISL_8152612	EPI_ISL_8152602	EPI_ISL_7949582
EPI_ISL_7949633	EPI_ISL_7949716	EPI_ISL_7949725	EPI_ISL_7949743	EPI_ISL_7949751
EPI_ISL_7949785	EPI_ISL_7949779	EPI_ISL_7949821	EPI_ISL_7949817	EPI_ISL_7949812
EPI_ISL_7949887	EPI_ISL_7949834	EPI_ISL_7949866	EPI_ISL_7949900	EPI_ISL_7949909
EPI_ISL_7949950	EPI_ISL_7949949	EPI_ISL_7949952	EPI_ISL_7950003	EPI_ISL_7950015
EPI_ISL_7950040	EPI_ISL_7950052	EPI_ISL_7950075	EPI_ISL_7950116	EPI_ISL_7950202
EPI_ISL_8151209	EPI_ISL_8151166	EPI_ISL_8147602	EPI_ISL_8147596	EPI_ISL_8147590
EPI_ISL_8147556	EPI_ISL_8145425	EPI_ISL_8145419	EPI_ISL_8145387	EPI_ISL_8145358
EPI_ISL_6945031	EPI_ISL_6944819	EPI_ISL_8125189	EPI_ISL_8125208	EPI_ISL_8125212
EPI_ISL_8125224	EPI_ISL_8125225	EPI_ISL_8125229	EPI_ISL_8125248	EPI_ISL_8125254
EPI_ISL_8125289	EPI_ISL_8125272	EPI_ISL_8125320	EPI_ISL_8125329	EPI_ISL_8125343
EPI_ISL_8125388	EPI_ISL_8125400	EPI_ISL_8125410	EPI_ISL_8125419	EPI_ISL_8125421
EPI_ISL_8125444	EPI_ISL_8131250	EPI_ISL_8131318	EPI_ISL_8131326	EPI_ISL_8131370
EPI_ISL_8131414	EPI_ISL_8131416	EPI_ISL_8131449	EPI_ISL_8131505	EPI_ISL_8051449
EPI_ISL_8051437	EPI_ISL_8051435	EPI_ISL_8051086	EPI_ISL_8294994	EPI_ISL_8295022
EPI_ISL_8295039	EPI_ISL_8295057	EPI_ISL_8295062	EPI_ISL_8295064	EPI_ISL_5914802
EPI_ISL_8007727	EPI_ISL_8007718	EPI_ISL_8007710	EPI_ISL_8007671	EPI_ISL_8007669
EPI_ISL_8007664	EPI_ISL_8007652	EPI_ISL_8007596	EPI_ISL_8007595	EPI_ISL_8006728
EPI_ISL_8006716	EPI_ISL_7996522	EPI_ISL_7996512	EPI_ISL_7996504	EPI_ISL_7996489
EPI_ISL_7996441	EPI_ISL_7996429	EPI_ISL_7996425	EPI_ISL_7996418	EPI_ISL_7996400
EPI_ISL_7991453	EPI_ISL_8356240	EPI_ISL_8356315	EPI_ISL_8356246	EPI_ISL_8356295
EPI_ISL_8356280	EPI_ISL_8356368	EPI_ISL_8356382	EPI_ISL_8356416	EPI_ISL_8356229
EPI_ISL_8356314	EPI_ISL_7950171	EPI_ISL_7950103	EPI_ISL_7950013	EPI_ISL_7950010
EPI_ISL_7949748	EPI_ISL_7949705	EPI_ISL_7936658	EPI_ISL_7935414	EPI_ISL_7935411
EPI_ISL_7935404	EPI_ISL_7935396	EPI_ISL_7924641	EPI_ISL_8377336	EPI_ISL_7886099
EPI_ISL_7886089	EPI_ISL_7880860	EPI_ISL_7880856	EPI_ISL_7880847	EPI_ISL_7880840
EPI_ISL_7880828	EPI_ISL_7880825	EPI_ISL_7880820	EPI_ISL_7880806	EPI_ISL_7880792
EPI_ISL_7880790	EPI_ISL_7880687	EPI_ISL_7880676	EPI_ISL_7880645	EPI_ISL_7880637

EPI_ISL_7880625	EPI_ISL_7880609	EPI_ISL_7880220	EPI_ISL_7880209	EPI_ISL_7880204
EPI_ISL_7880193	EPI_ISL_7880181	EPI_ISL_7877195	EPI_ISL_7877189	EPI_ISL_7877159
EPI_ISL_7877138	EPI_ISL_7877135	EPI_ISL_7877120	EPI_ISL_7870097	EPI_ISL_7870092
EPI_ISL_7870076	EPI_ISL_7869973	EPI_ISL_7869957	EPI_ISL_7869934	EPI_ISL_7869920
EPI_ISL_7869890	EPI_ISL_7869825	EPI_ISL_7869801	EPI_ISL_8381565	EPI_ISL_8381563
EPI_ISL_7837000	EPI_ISL_7836980	EPI_ISL_7836923	EPI_ISL_7836904	EPI_ISL_7836854
EPI_ISL_7836852	EPI_ISL_7836576	EPI_ISL_7836550	EPI_ISL_7835403	EPI_ISL_7824093
EPI_ISL_7803965	EPI_ISL_7773930	EPI_ISL_7773915	EPI_ISL_7773868	EPI_ISL_7773821
EPI_ISL_7773799	EPI_ISL_5885425	EPI_ISL_5885394	EPI_ISL_5885379	EPI_ISL_5885370
EPI_ISL_7740377	EPI_ISL_7740370	EPI_ISL_7740361	EPI_ISL_7740348	EPI_ISL_7740336
EPI_ISL_7740333	EPI_ISL_8377077	EPI_ISL_8377083	EPI_ISL_7691199	EPI_ISL_7691137
EPI_ISL_7688285	EPI_ISL_7688250	EPI_ISL_7688219	EPI_ISL_7688175	EPI_ISL_7669072
EPI_ISL_7669020	EPI_ISL_7668796	EPI_ISL_7668735	EPI_ISL_7668697	EPI_ISL_7668680
EPI_ISL_7668670	EPI_ISL_8377306	EPI_ISL_7662285	EPI_ISL_7662217	EPI_ISL_7661667
EPI_ISL_7653488	EPI_ISL_7653474	EPI_ISL_7653464	EPI_ISL_7653450	EPI_ISL_7653423
EPI_ISL_7653354	EPI_ISL_7653351	EPI_ISL_7545416	EPI_ISL_7545410	EPI_ISL_7545398
EPI_ISL_7461491	EPI_ISL_7461241	EPI_ISL_5860460	EPI_ISL_7406114	EPI_ISL_7404487
EPI_ISL_7404485	EPI_ISL_7404474	EPI_ISL_7404225	EPI_ISL_7404159	EPI_ISL_7403961
EPI_ISL_7398738	EPI_ISL_7398733	EPI_ISL_7398732	EPI_ISL_7398725	EPI_ISL_7398719
EPI_ISL_7398717	EPI_ISL_7398711	EPI_ISL_7398671	EPI_ISL_7398669	EPI_ISL_7398664
EPI_ISL_7398650	EPI_ISL_5854226	EPI_ISL_7365498	EPI_ISL_7365472	EPI_ISL_7365376
EPI_ISL_7365270	EPI_ISL_7365263	EPI_ISL_7365224	EPI_ISL_7365213	EPI_ISL_7364294
EPI_ISL_7364270	EPI_ISL_7364252	EPI_ISL_7320654	EPI_ISL_7320618	EPI_ISL_7320549
EPI_ISL_7320163	EPI_ISL_7320085	EPI_ISL_7320069	EPI_ISL_7320060	EPI_ISL_7283330
EPI_ISL_7283305	EPI_ISL_7283304	EPI_ISL_7283264	EPI_ISL_7283091	EPI_ISL_8035341
EPI_ISL_7226207	EPI_ISL_7226099	EPI_ISL_7226095	EPI_ISL_7226090	EPI_ISL_7226077
EPI_ISL_7226025	EPI_ISL_7154253	EPI_ISL_7154247	EPI_ISL_7154226	EPI_ISL_7154206
EPI_ISL_7154180	EPI_ISL_7128209	EPI_ISL_7128114	EPI_ISL_7051622	EPI_ISL_7051469
EPI_ISL_7051433	EPI_ISL_5796206	EPI_ISL_5796134	EPI_ISL_6986478	EPI_ISL_6986475
EPI_ISL_6986428	EPI_ISL_6986286	EPI_ISL_6986006	EPI_ISL_6985985	EPI_ISL_6986090
EPI_ISL_6109860	EPI_ISL_6944995	EPI_ISL_6944917	EPI_ISL_6944878	EPI_ISL_6944788
EPI_ISL_6932037	EPI_ISL_6931961	EPI_ISL_6931960	EPI_ISL_6931675	EPI_ISL_6931081
EPI_ISL_6931048	EPI_ISL_6931043	EPI_ISL_6931032	EPI_ISL_6931025	EPI_ISL_6931017
EPI_ISL_6915169	EPI_ISL_6915166	EPI_ISL_6915097	EPI_ISL_8318245	EPI_ISL_6857013
EPI_ISL_6844382	EPI_ISL_6833172	EPI_ISL_6812618	EPI_ISL_6812616	EPI_ISL_6812593
EPI_ISL_6795798	EPI_ISL_6795794	EPI_ISL_5771092	EPI_ISL_5770995	EPI_ISL_5770909
EPI_ISL_6778517	EPI_ISL_6775138	EPI_ISL_6775117	EPI_ISL_6774768	EPI_ISL_6774447
EPI_ISL_6774431	EPI_ISL_6774136	EPI_ISL_6718023	EPI_ISL_6717992	EPI_ISL_6709867
EPI_ISL_6704828	EPI_ISL_6704549	EPI_ISL_6704420	EPI_ISL_6700536	EPI_ISL_6666328
EPI_ISL_6666301	EPI_ISL_6657444	EPI_ISL_6656692	EPI_ISL_6656612	EPI_ISL_6656304
EPI_ISL_6641732	EPI_ISL_6641694	EPI_ISL_6641631	EPI_ISL_6641541	EPI_ISL_6590743
EPI_ISL_6565224	EPI_ISL_6565088	EPI_ISL_6563125	EPI_ISL_6507310	EPI_ISL_6507287
EPI_ISL_6475311	EPI_ISL_6475294	EPI_ISL_6471674	EPI_ISL_6471643	EPI_ISL_6471641
EPI_ISL_6469401	EPI_ISL_6469390	EPI_ISL_6469334	EPI_ISL_6444346	EPI_ISL_6444124
EPI_ISL_7051404	EPI_ISL_6369725	EPI_ISL_6369416	EPI_ISL_5686656	EPI_ISL_5655342
EPI_ISL_6229338	EPI_ISL_6229329	EPI_ISL_5524418	EPI_ISL_5524407	EPI_ISL_5509391
EPI_ISL_6251485	EPI_ISL_5331629	EPI_ISL_5331708	EPI_ISL_5331602	EPI_ISL_5331562
EPI_ISL_6272712	EPI_ISL_5307492	EPI_ISL_5268302	EPI_ISL_6324509	EPI_ISL_6324520
EPI_ISL_6324560	EPI_ISL_6324578	EPI_ISL_6324380	EPI_ISL_5069838	EPI_ISL_4943342
EPI_ISL_4943208	EPI_ISL_5408329	EPI_ISL_7128153	EPI_ISL_7128260	EPI_ISL_7128235
EPI_ISL_4748072	EPI_ISL_6369866	EPI_ISL_7129988	EPI_ISL_7129990	EPI_ISL_7129993
EPI_ISL_4551628	EPI_ISL_4547742	EPI_ISL_6775243	EPI_ISL_4472544	EPI_ISL_4472459
EPI_ISL_7141851	EPI_ISL_5509275	EPI_ISL_6442915	EPI_ISL_5543578	EPI_ISL_4256330
EPI_ISL_6475267	EPI_ISL_7154187	EPI_ISL_7154195	EPI_ISL_7154215	EPI_ISL_7154200
EPI_ISL_7154262	EPI_ISL_7154258	EPI_ISL_5602935	EPI_ISL_5602965	EPI_ISL_5650012

EPI_ISL_7154198	EPI_ISL_7154301	EPI_ISL_6590750	EPI_ISL_6590621	EPI_ISL_6590640
EPI_ISL_7283151	EPI_ISL_7364286	EPI_ISL_7364202	EPI_ISL_7364221	EPI_ISL_7365171
EPI_ISL_6656639	EPI_ISL_6656645	EPI_ISL_7364160	EPI_ISL_7364153	EPI_ISL_6655032
EPI_ISL_6655046	EPI_ISL_6657576	EPI_ISL_6657317	EPI_ISL_7368413	EPI_ISL_7365307
EPI_ISL_7365343	EPI_ISL_7367853	EPI_ISL_7367863	EPI_ISL_7365202	EPI_ISL_7365232
EPI_ISL_7365239	EPI_ISL_7368390	EPI_ISL_7367790	EPI_ISL_7364323	EPI_ISL_7364342
EPI_ISL_7364360	EPI_ISL_7364383	EPI_ISL_7364388	EPI_ISL_7364377	EPI_ISL_7368489
EPI_ISL_7927046	EPI_ISL_6718028	EPI_ISL_7404227	EPI_ISL_7398689	EPI_ISL_7398661
EPI_ISL_7404489	EPI_ISL_7404497	EPI_ISL_5924743	EPI_ISL_8173101	EPI_ISL_5853552
EPI_ISL_7623031	EPI_ISL_6931055	EPI_ISL_5854251	EPI_ISL_7653370	EPI_ISL_7653384
EPI_ISL_5854182	EPI_ISL_5860697	EPI_ISL_7662171	EPI_ISL_7661678	EPI_ISL_7661660
EPI_ISL_7662286	EPI_ISL_7662284	EPI_ISL_7661795	EPI_ISL_7661780	EPI_ISL_7668985
EPI_ISL_7675874	EPI_ISL_7675865	EPI_ISL_7691147	EPI_ISL_7691188	EPI_ISL_5331936
EPI_ISL_5332048	EPI_ISL_5332069	EPI_ISL_5332113	EPI_ISL_5332108	EPI_ISL_5332144
EPI_ISL_7731798	EPI_ISL_5639979	EPI_ISL_5640125	EPI_ISL_5738479	EPI_ISL_5795943
EPI_ISL_5795950	EPI_ISL_5795980	EPI_ISL_5795971	EPI_ISL_5796015	EPI_ISL_6012991
EPI_ISL_6110115	EPI_ISL_6110169	EPI_ISL_6110236	EPI_ISL_6110682	EPI_ISL_6110649
EPI_ISL_6438895	EPI_ISL_6442547	EPI_ISL_6443506	EPI_ISL_6443651	EPI_ISL_6443902
EPI_ISL_6443859	EPI_ISL_7775295	EPI_ISL_7773814	EPI_ISL_7835389	EPI_ISL_7545624
EPI_ISL_7545388	EPI_ISL_5999424	EPI_ISL_7949927	EPI_ISL_7996420	EPI_ISL_8007616
EPI_ISL_8145356	EPI_ISL_8051089	EPI_ISL_7949860	EPI_ISL_7996503	EPI_ISL_7622978
EPI_ISL_7622904	EPI_ISL_7622970	EPI_ISL_7622875	EPI_ISL_7622945	EPI_ISL_7622998
EPI_ISL_7622962	EPI_ISL_7622883	EPI_ISL_7622866	EPI_ISL_7950074	EPI_ISL_8377274
EPI_ISL_7623040	EPI_ISL_7622946	EPI_ISL_7622922	EPI_ISL_7623041	EPI_ISL_7622961
EPI_ISL_7623007	EPI_ISL_8173062	EPI_ISL_6110820	EPI_ISL_8152562	EPI_ISL_6229369
EPI_ISL_7924575	EPI_ISL_7869984	EPI_ISL_8338675	EPI_ISL_7949884	EPI_ISL_6324518
EPI_ISL_5999425	EPI_ISL_7653379	EPI_ISL_7653486	EPI_ISL_7661738	EPI_ISL_7661835
EPI_ISL_7662247	EPI_ISL_7662246	EPI_ISL_7661655	EPI_ISL_7661663	EPI_ISL_7661754
EPI_ISL_7668665	EPI_ISL_7668978	EPI_ISL_7668600	EPI_ISL_7949764	EPI_ISL_7886164
EPI_ISL_8035364	EPI_ISL_8294576	EPI_ISL_7949615	EPI_ISL_8294556	EPI_ISL_7675837
EPI_ISL_7675846	EPI_ISL_7675851	EPI_ISL_7675852	EPI_ISL_7668884	EPI_ISL_6857256
EPI_ISL_7688190	EPI_ISL_7688173	EPI_ISL_7688259	EPI_ISL_7688183	EPI_ISL_7688265
EPI_ISL_7688270	EPI_ISL_7731721	EPI_ISL_7731732	EPI_ISL_6028095	EPI_ISL_8151177
EPI_ISL_6027991	EPI_ISL_6028106	EPI_ISL_7836696	EPI_ISL_8377286	EPI_ISL_8338686
EPI_ISL_7691120	EPI_ISL_6034365	EPI_ISL_7928145	EPI_ISL_7869760	EPI_ISL_7691108
EPI_ISL_7691123	EPI_ISL_7691132	EPI_ISL_7691189	EPI_ISL_7691208	EPI_ISL_7691214
EPI_ISL_7949912	EPI_ISL_7950059	EPI_ISL_7928154	EPI_ISL_8006627	EPI_ISL_8051078
EPI_ISL_7710842	EPI_ISL_8125287	EPI_ISL_7710848	EPI_ISL_7869879	EPI_ISL_7710776
EPI_ISL_7710831	EPI_ISL_7710836	EPI_ISL_7935403	EPI_ISL_7949588	EPI_ISL_8125380
EPI_ISL_7949854	EPI_ISL_7924647	EPI_ISL_7924654	EPI_ISL_7949618	EPI_ISL_7949569
EPI_ISL_7950170	EPI_ISL_7949853	EPI_ISL_7950064	EPI_ISL_6324620	EPI_ISL_7740373
EPI_ISL_8356269	EPI_ISL_8356256	EPI_ISL_7740349	EPI_ISL_6125811	EPI_ISL_8164890
EPI_ISL_7731774	EPI_ISL_7803610	EPI_ISL_7803966	EPI_ISL_7775293	EPI_ISL_8381542
EPI_ISL_7834657	EPI_ISL_7740369	EPI_ISL_7740331	EPI_ISL_8147538	EPI_ISL_8170538
EPI_ISL_7759526	EPI_ISL_7950092	EPI_ISL_8356292	EPI_ISL_8051065	EPI_ISL_8351353
EPI_ISL_7869960	EPI_ISL_7869964	EPI_ISL_7775303	EPI_ISL_7886065	EPI_ISL_7773932
EPI_ISL_7949893	EPI_ISL_8356293	EPI_ISL_8420893	EPI_ISL_4542146	EPI_ISL_6229301
EPI_ISL_8294583	EPI_ISL_6251498	EPI_ISL_6110991	EPI_ISL_8151204	EPI_ISL_7824084
EPI_ISL_7836888	EPI_ISL_7835387	EPI_ISL_7836820	EPI_ISL_7835394	EPI_ISL_7836616
EPI_ISL_7836558	EPI_ISL_7836893	EPI_ISL_7836769	EPI_ISL_7836837	EPI_ISL_7836776
EPI_ISL_7836797	EPI_ISL_7836734	EPI_ISL_7836981	EPI_ISL_7837011	EPI_ISL_7836843
EPI_ISL_7836729	EPI_ISL_7836838	EPI_ISL_7836736	EPI_ISL_7836964	EPI_ISL_7836850
EPI_ISL_7836932	EPI_ISL_7836743	EPI_ISL_7924656	EPI_ISL_8188647	EPI_ISL_6324332
EPI_ISL_6324422	EPI_ISL_6324622	EPI_ISL_6324566	EPI_ISL_4633053	EPI_ISL_7950195
EPI_ISL_7950084	EPI_ISL_8131496	EPI_ISL_8131296	EPI_ISL_8381549	EPI_ISL_7283075

EPI_ISL_8172997	EPI_ISL_7836962	EPI_ISL_7836600	EPI_ISL_7836689	EPI_ISL_7836857
EPI_ISL_7836715	EPI_ISL_8131369	EPI_ISL_8131361	EPI_ISL_8131367	EPI_ISL_8131484
EPI_ISL_8035361	EPI_ISL_7935398	EPI_ISL_7949768	EPI_ISL_8351368	EPI_ISL_7949572
EPI_ISL_8051064	EPI_ISL_7877147	EPI_ISL_7949798	EPI_ISL_7949998	EPI_ISL_7877169
EPI_ISL_7886173	EPI_ISL_8131473	EPI_ISL_7364281	EPI_ISL_7364305	EPI_ISL_7367626
EPI_ISL_7367663	EPI_ISL_7368421	EPI_ISL_7368464	EPI_ISL_7368479	EPI_ISL_7398673
EPI_ISL_7950126	EPI_ISL_7869975	EPI_ISL_8172990	EPI_ISL_8173081	EPI_ISL_8131477
EPI_ISL_6700547	EPI_ISL_6774587	EPI_ISL_6774529	EPI_ISL_6775209	EPI_ISL_6775207
EPI_ISL_6775225	EPI_ISL_6775194	EPI_ISL_6774619	EPI_ISL_6775062	EPI_ISL_6774574
EPI_ISL_6774511	EPI_ISL_6774551	EPI_ISL_6774550	EPI_ISL_7461221	EPI_ISL_7461385
EPI_ISL_7461412	EPI_ISL_7461482	EPI_ISL_6774300	EPI_ISL_6774238	EPI_ISL_6774232
EPI_ISL_6774278	EPI_ISL_6774168	EPI_ISL_7461571	EPI_ISL_6774911	EPI_ISL_6774913
EPI_ISL_6774935	EPI_ISL_6775292	EPI_ISL_6778537	EPI_ISL_6795785	EPI_ISL_6795782
EPI_ISL_6795764	EPI_ISL_6774602	EPI_ISL_6810761	EPI_ISL_6810776	EPI_ISL_6810726
EPI_ISL_6810730	EPI_ISL_6812609	EPI_ISL_7886156	EPI_ISL_7836959	EPI_ISL_8125221
EPI_ISL_7668945	EPI_ISL_7668587	EPI_ISL_8125315	EPI_ISL_6324402	EPI_ISL_8006715
EPI_ISL_8125166	EPI_ISL_8131520	EPI_ISL_8131508	EPI_ISL_8131413	EPI_ISL_8131321
EPI_ISL_8131289	EPI_ISL_6833095	EPI_ISL_6833157	EPI_ISL_8147545	EPI_ISL_8147565
EPI_ISL_8151157	EPI_ISL_8151194	EPI_ISL_8125269	EPI_ISL_8152621	EPI_ISL_6844311
EPI_ISL_8165705	EPI_ISL_6844326	EPI_ISL_6666266	EPI_ISL_8164985	EPI_ISL_8165804
EPI_ISL_6847119	EPI_ISL_6810743	EPI_ISL_4882075	EPI_ISL_8173108	EPI_ISL_8173104
EPI_ISL_8173052	EPI_ISL_8173047	EPI_ISL_7880778	EPI_ISL_6852326	EPI_ISL_6852370
EPI_ISL_6852353	EPI_ISL_6852400	EPI_ISL_6021442	EPI_ISL_4891961	EPI_ISL_6857056
EPI_ISL_6857029	EPI_ISL_6858486	EPI_ISL_6857153	EPI_ISL_6857164	EPI_ISL_6857129
EPI_ISL_6858542	EPI_ISL_6858549	EPI_ISL_6858550	EPI_ISL_6858555	EPI_ISL_6858527
EPI_ISL_6858507	EPI_ISL_6858501	EPI_ISL_6858510	EPI_ISL_6858575	EPI_ISL_6857345
EPI_ISL_6857321	EPI_ISL_8188672	EPI_ISL_6833178	EPI_ISL_6774683	EPI_ISL_8377340
EPI_ISL_8216483	EPI_ISL_8216495	EPI_ISL_8216514	EPI_ISL_6866328	EPI_ISL_6866331
EPI_ISL_6866381	EPI_ISL_6866356	EPI_ISL_6866443	EPI_ISL_6866417	EPI_ISL_6844306
EPI_ISL_6858514	EPI_ISL_8131333	EPI_ISL_7545597	EPI_ISL_6021777	EPI_ISL_5640109
EPI_ISL_8188681	EPI_ISL_8164999	EPI_ISL_8125182	EPI_ISL_8131499	EPI_ISL_8151199
EPI_ISL_7949739	EPI_ISL_6944753	EPI_ISL_6944974	EPI_ISL_8035359	EPI_ISL_7675855
EPI_ISL_8294572	EPI_ISL_8151173	EPI_ISL_6471663	EPI_ISL_6475243	EPI_ISL_6717969
EPI_ISL_8125191	EPI_ISL_8530773	EPI_ISL_9664910	EPI_ISL_9664906	EPI_ISL_9658328
EPI_ISL_9658292	EPI_ISL_8524345	EPI_ISL_8524331	EPI_ISL_9651598	EPI_ISL_9650508
EPI_ISL_8519140	EPI_ISL_8519130	EPI_ISL_9614873	EPI_ISL_9614591	EPI_ISL_9614584
EPI_ISL_9612785	EPI_ISL_9600743	EPI_ISL_9600648	EPI_ISL_9600572	EPI_ISL_9589540
EPI_ISL_9822915	EPI_ISL_9572056	EPI_ISL_9572044	EPI_ISL_9572042	EPI_ISL_9572041
EPI_ISL_9572036	EPI_ISL_10134817	EPI_ISL_9515433	EPI_ISL_9515409	EPI_ISL_9512487
EPI_ISL_9512482	EPI_ISL_9512481	EPI_ISL_9506428	EPI_ISL_9506385	EPI_ISL_9506379
EPI_ISL_9506270	EPI_ISL_9503339	EPI_ISL_9503264	EPI_ISL_8481718	EPI_ISL_8481673
EPI_ISL_9498975	EPI_ISL_9498870	EPI_ISL_9498849	EPI_ISL_8476558	EPI_ISL_8476549
EPI_ISL_8476503	EPI_ISL_8476502	EPI_ISL_8475919	EPI_ISL_9492876	EPI_ISL_9491004
EPI_ISL_9489510	EPI_ISL_9489440	EPI_ISL_8460250	EPI_ISL_8458737	EPI_ISL_9854604
EPI_ISL_9854542	EPI_ISL_9854536	EPI_ISL_9854434	EPI_ISL_9854419	EPI_ISL_9357097
EPI_ISL_9352524	EPI_ISL_9859354	EPI_ISL_9316003	EPI_ISL_9290078	EPI_ISL_9352702
EPI_ISL_9243378	EPI_ISL_9243361	EPI_ISL_9874999	EPI_ISL_9179844	EPI_ISL_9179839
EPI_ISL_9165109	EPI_ISL_9165108	EPI_ISL_9165100	EPI_ISL_9163135	EPI_ISL_9161511
EPI_ISL_9161498	EPI_ISL_9161478	EPI_ISL_9161474	EPI_ISL_9157020	EPI_ISL_9156957
EPI_ISL_9156706	EPI_ISL_9140772	EPI_ISL_9140674	EPI_ISL_9140629	EPI_ISL_9131359
EPI_ISL_9131355	EPI_ISL_8925406	EPI_ISL_9650406	EPI_ISL_9069119	EPI_ISL_9069118
EPI_ISL_9059204	EPI_ISL_9030250	EPI_ISL_9030209	EPI_ISL_9030203	EPI_ISL_9021564
EPI_ISL_9021549	EPI_ISL_9012744	EPI_ISL_9012708	EPI_ISL_8985785	EPI_ISL_8985740
EPI_ISL_8985716	EPI_ISL_8985689	EPI_ISL_8985636	EPI_ISL_9822910	EPI_ISL_8925417
EPI_ISL_8925414	EPI_ISL_8925401	EPI_ISL_8925370	EPI_ISL_8893452	EPI_ISL_8882308

EPI_ISL_8882278	EPI_ISL_8851490	EPI_ISL_8851231	EPI_ISL_8838084	EPI_ISL_8830254
EPI_ISL_8829664	EPI_ISL_8829658	EPI_ISL_9977809	EPI_ISL_9977895	EPI_ISL_9976447
EPI_ISL_8808615	EPI_ISL_8799597	EPI_ISL_8799577	EPI_ISL_9981793	EPI_ISL_8787807
EPI_ISL_8787787	EPI_ISL_8785500	EPI_ISL_8785485	EPI_ISL_8783197	EPI_ISL_8783196
EPI_ISL_8783170	EPI_ISL_8764327	EPI_ISL_9985763	EPI_ISL_8744812	EPI_ISL_8744808
EPI_ISL_8744791	EPI_ISL_8718636	EPI_ISL_8702124	EPI_ISL_10014562	EPI_ISL_8692625
EPI_ISL_8672128	EPI_ISL_9503020	EPI_ISL_9822720	EPI_ISL_8656772	EPI_ISL_8642803
EPI_ISL_8931076	EPI_ISL_8614229	EPI_ISL_8609718	EPI_ISL_8609700	EPI_ISL_8609661
EPI_ISL_8608740	EPI_ISL_8591525	EPI_ISL_8591501	EPI_ISL_8589925	EPI_ISL_8589880
EPI_ISL_8589858	EPI_ISL_8582185	EPI_ISL_8582041	EPI_ISL_8582013	EPI_ISL_8581986
EPI_ISL_8569043	EPI_ISL_8569039	EPI_ISL_8569034	EPI_ISL_8540948	EPI_ISL_8540939
EPI_ISL_8540429	EPI_ISL_8540419	EPI_ISL_8540411	EPI_ISL_8540403	EPI_ISL_8540402
EPI_ISL_8540375	EPI_ISL_8540361	EPI_ISL_9650525	EPI_ISL_10136933	EPI_ISL_8558310
EPI_ISL_8558325	EPI_ISL_9316034	EPI_ISL_9509634	EPI_ISL_9512535	EPI_ISL_9375836
EPI_ISL_8658374	EPI_ISL_8658417	EPI_ISL_8658447	EPI_ISL_8658520	EPI_ISL_8658556
EPI_ISL_8658564	EPI_ISL_8658617	EPI_ISL_8716575	EPI_ISL_8716627	EPI_ISL_8716721
EPI_ISL_8716748	EPI_ISL_8716774	EPI_ISL_8716805	EPI_ISL_8716816	EPI_ISL_9976642
EPI_ISL_9985542	EPI_ISL_8582039	EPI_ISL_8799734	EPI_ISL_8800005	EPI_ISL_8800036
EPI_ISL_8800051	EPI_ISL_8831260	EPI_ISL_8831281	EPI_ISL_8831289	EPI_ISL_8831312
EPI_ISL_8831314	EPI_ISL_8831327	EPI_ISL_8831505	EPI_ISL_8831589	EPI_ISL_8831587
EPI_ISL_8837435	EPI_ISL_8837439	EPI_ISL_8837537	EPI_ISL_8837610	EPI_ISL_8837634
EPI_ISL_8837705	EPI_ISL_8837768	EPI_ISL_8837802	EPI_ISL_8837813	EPI_ISL_8837842
EPI_ISL_8837853	EPI_ISL_8837868	EPI_ISL_8837870	EPI_ISL_8838018	EPI_ISL_8838030
EPI_ISL_8838033	EPI_ISL_8838053	EPI_ISL_9752445	EPI_ISL_8975411	EPI_ISL_8975424
EPI_ISL_8975441	EPI_ISL_8975515	EPI_ISL_8975667	EPI_ISL_8975773	EPI_ISL_8975827
EPI_ISL_8976046	EPI_ISL_8976050	EPI_ISL_8420963	EPI_ISL_8420967	EPI_ISL_9753050
EPI_ISL_9985450	EPI_ISL_10136950	EPI_ISL_10138750	EPI_ISL_9111393	EPI_ISL_9111410
EPI_ISL_9111712	EPI_ISL_9111824	EPI_ISL_9111833	EPI_ISL_9658253	EPI_ISL_9154212
EPI_ISL_9154214	EPI_ISL_9154235	EPI_ISL_9154355	EPI_ISL_9154359	EPI_ISL_9154361
EPI_ISL_9154519	EPI_ISL_9154639	EPI_ISL_9154680	EPI_ISL_9154740	EPI_ISL_9154917
EPI_ISL_9163206	EPI_ISL_9202352	EPI_ISL_9202410	EPI_ISL_9202434	EPI_ISL_9202539
EPI_ISL_9202591	EPI_ISL_9202680	EPI_ISL_9203009	EPI_ISL_9202993	EPI_ISL_9202995
EPI_ISL_9203010	EPI_ISL_9267063	EPI_ISL_9267093	EPI_ISL_9267280	EPI_ISL_9375816
EPI_ISL_9375948	EPI_ISL_9375938	EPI_ISL_9376075	EPI_ISL_9376106	EPI_ISL_9376127
EPI_ISL_9376228	EPI_ISL_9267406	EPI_ISL_9392147	EPI_ISL_9392204	EPI_ISL_9392305
EPI_ISL_9392280	EPI_ISL_9392320	EPI_ISL_9392468	EPI_ISL_9392443	EPI_ISL_9427017
EPI_ISL_9431464	EPI_ISL_9431532	EPI_ISL_9431739	EPI_ISL_9431787	EPI_ISL_9431789
EPI_ISL_9431912	EPI_ISL_9431981	EPI_ISL_9432035	EPI_ISL_9432095	EPI_ISL_9432417
EPI_ISL_9432443	EPI_ISL_9432474	EPI_ISL_9432548	EPI_ISL_9432644	EPI_ISL_9509283
EPI_ISL_9508877	EPI_ISL_9508900	EPI_ISL_9508908	EPI_ISL_9508973	EPI_ISL_9508987
EPI_ISL_9508995	EPI_ISL_9509000	EPI_ISL_9509032	EPI_ISL_9509161	EPI_ISL_9509115
EPI_ISL_9509213	EPI_ISL_9509450	EPI_ISL_9509484	EPI_ISL_9509559	EPI_ISL_9509578
EPI_ISL_9509573	EPI_ISL_9509640	EPI_ISL_9509684	EPI_ISL_9509715	EPI_ISL_9509736
EPI_ISL_9509738	EPI_ISL_9509744	EPI_ISL_9509747	EPI_ISL_9534158	EPI_ISL_10140365
EPI_ISL_9673188	EPI_ISL_9673239	EPI_ISL_9673227	EPI_ISL_9673269	EPI_ISL_9673276
EPI_ISL_9673280	EPI_ISL_9673322	EPI_ISL_9673348	EPI_ISL_9673368	EPI_ISL_9673386
EPI_ISL_9673390	EPI_ISL_9673508	EPI_ISL_9673539	EPI_ISL_9687853	EPI_ISL_9687862
EPI_ISL_9687868	EPI_ISL_9687950	EPI_ISL_9688024	EPI_ISL_9688241	EPI_ISL_9688263
EPI_ISL_9705892	EPI_ISL_9706004	EPI_ISL_9706068	EPI_ISL_9705950	EPI_ISL_9706041
EPI_ISL_9706091	EPI_ISL_9706076	EPI_ISL_9706350	EPI_ISL_9712704	EPI_ISL_9712843
EPI_ISL_9712863	EPI_ISL_9712937	EPI_ISL_9712970	EPI_ISL_9712948	EPI_ISL_9712985
EPI_ISL_9712991	EPI_ISL_9713009	EPI_ISL_9713023	EPI_ISL_9713087	EPI_ISL_9713123
EPI_ISL_9713151	EPI_ISL_9713217	EPI_ISL_9713229	EPI_ISL_9713266	EPI_ISL_9713303
EPI_ISL_9713415	EPI_ISL_9713446	EPI_ISL_9713631	EPI_ISL_9706167	EPI_ISL_9748707
EPI_ISL_9748846	EPI_ISL_9748855	EPI_ISL_9748945	EPI_ISL_9748982	EPI_ISL_9749050

EPI_ISL_9749053	EPI_ISL_9749073	EPI_ISL_9820223	EPI_ISL_9820254	EPI_ISL_9820289
EPI_ISL_9820283	EPI_ISL_9820324	EPI_ISL_9820356	EPI_ISL_9820398	EPI_ISL_9820382
EPI_ISL_9820401	EPI_ISL_9820405	EPI_ISL_9820435	EPI_ISL_9820461	EPI_ISL_9820462
EPI_ISL_9820473	EPI_ISL_9830324	EPI_ISL_9830305	EPI_ISL_9830328	EPI_ISL_9908406
EPI_ISL_9908418	EPI_ISL_9908481	EPI_ISL_9908511	EPI_ISL_9908517	EPI_ISL_9908520
EPI_ISL_9908541	EPI_ISL_9908554	EPI_ISL_9908617	EPI_ISL_9908652	EPI_ISL_9908731
EPI_ISL_9908741	EPI_ISL_9908806	EPI_ISL_9908847	EPI_ISL_9908848	EPI_ISL_9908895
EPI_ISL_9908963	EPI_ISL_9908979	EPI_ISL_9908981	EPI_ISL_9908985	EPI_ISL_9908987
EPI_ISL_9909022	EPI_ISL_9909023	EPI_ISL_9706371	EPI_ISL_10069599	EPI_ISL_10069522
EPI_ISL_10065425	EPI_ISL_10065413	EPI_ISL_9356772	EPI_ISL_9877572	EPI_ISL_9876122
EPI_ISL_9876062	EPI_ISL_9876043	EPI_ISL_9875945	EPI_ISL_9874989	EPI_ISL_9846268
EPI_ISL_9846085	EPI_ISL_9846083	EPI_ISL_9844320	EPI_ISL_9844282	EPI_ISL_10136874
EPI_ISL_9959157	EPI_ISL_9959163	EPI_ISL_9959168	EPI_ISL_9959202	EPI_ISL_9959206
EPI_ISL_9959217	EPI_ISL_10134775	EPI_ISL_9977762	EPI_ISL_9977797	EPI_ISL_9977831
EPI_ISL_9977850	EPI_ISL_9977877	EPI_ISL_9977924	EPI_ISL_9977930	EPI_ISL_9977975
EPI_ISL_9977994	EPI_ISL_9978026	EPI_ISL_9978021	EPI_ISL_9978054	EPI_ISL_9978064
EPI_ISL_9978083	EPI_ISL_9978100	EPI_ISL_9978091	EPI_ISL_9978096	EPI_ISL_9978115
EPI_ISL_9978128	EPI_ISL_9978141	EPI_ISL_9978144	EPI_ISL_9978164	EPI_ISL_9978196
EPI_ISL_9978215	EPI_ISL_9984840	EPI_ISL_9984847	EPI_ISL_9984855	EPI_ISL_9984853
EPI_ISL_9984891	EPI_ISL_9984901	EPI_ISL_9984903	EPI_ISL_9984917	EPI_ISL_9984922
EPI_ISL_9984924	EPI_ISL_9984962	EPI_ISL_9984981	EPI_ISL_9984969	EPI_ISL_9985040
EPI_ISL_9985050	EPI_ISL_9985061	EPI_ISL_9985081	EPI_ISL_9985105	EPI_ISL_9985123
EPI_ISL_9985139	EPI_ISL_9985179	EPI_ISL_9985205	EPI_ISL_9985234	EPI_ISL_9985273
EPI_ISL_9985340	EPI_ISL_9985347	EPI_ISL_9985406	EPI_ISL_9985410	EPI_ISL_9985415
EPI_ISL_9985439	EPI_ISL_9985457	EPI_ISL_9985476	EPI_ISL_9985487	EPI_ISL_9985509
EPI_ISL_9985523	EPI_ISL_9985541	EPI_ISL_9985566	EPI_ISL_9985581	EPI_ISL_9985573
EPI_ISL_9985579	EPI_ISL_9985614	EPI_ISL_9985621	EPI_ISL_9985637	EPI_ISL_9985633
EPI_ISL_9985634	EPI_ISL_9985638	EPI_ISL_9985662	EPI_ISL_9985671	EPI_ISL_9985687
EPI_ISL_9985693	EPI_ISL_9985698	EPI_ISL_9985720	EPI_ISL_9985726	EPI_ISL_9985727
EPI_ISL_9985778	EPI_ISL_10088010	EPI_ISL_10088022	EPI_ISL_10088051	EPI_ISL_10088055
EPI_ISL_10088072	EPI_ISL_10088087	EPI_ISL_10088111	EPI_ISL_10088116	EPI_ISL_10088122
EPI_ISL_10088140	EPI_ISL_10088136	EPI_ISL_10088221	EPI_ISL_10088219	EPI_ISL_10088262
EPI_ISL_10088240	EPI_ISL_10088258	EPI_ISL_10088294	EPI_ISL_10088272	EPI_ISL_10088273
EPI_ISL_10088276	EPI_ISL_10088282	EPI_ISL_10088287	EPI_ISL_10088292	EPI_ISL_10088338
EPI_ISL_10088394	EPI_ISL_10114934	EPI_ISL_10115192	EPI_ISL_10115193	EPI_ISL_10115241
EPI_ISL_10115248	EPI_ISL_10115275	EPI_ISL_10115303	EPI_ISL_10115311	EPI_ISL_10115353
EPI_ISL_10115369	EPI_ISL_10115449	EPI_ISL_10115463	EPI_ISL_10115465	EPI_ISL_10115507
EPI_ISL_10115520	EPI_ISL_10115540	EPI_ISL_10115541	EPI_ISL_9846275	EPI_ISL_8481692
EPI_ISL_8481721	EPI_ISL_8481702	EPI_ISL_8582044	EPI_ISL_8568993	EPI_ISL_8581407
EPI_ISL_8581403	EPI_ISL_8582141	EPI_ISL_8582121	EPI_ISL_8589871	EPI_ISL_8716845
EPI_ISL_8642824	EPI_ISL_8690119	EPI_ISL_8693911	EPI_ISL_8702167	EPI_ISL_8709068
EPI_ISL_8712615	EPI_ISL_8712643	EPI_ISL_8716656	EPI_ISL_8716723	EPI_ISL_8716820
EPI_ISL_8764302	EPI_ISL_8799608	EPI_ISL_8799630	EPI_ISL_8799695	EPI_ISL_8806217
EPI_ISL_8837976	EPI_ISL_8851500	EPI_ISL_8975616	EPI_ISL_8625815	EPI_ISL_8831522
EPI_ISL_8882345	EPI_ISL_8420960	EPI_ISL_8712626	EPI_ISL_8530813	EPI_ISL_9069123
EPI_ISL_9069122	EPI_ISL_8589883	EPI_ISL_8481675	EPI_ISL_8851284	EPI_ISL_8481663
EPI_ISL_9111719	EPI_ISL_8917285	EPI_ISL_8712564	EPI_ISL_9154808	EPI_ISL_9154572
EPI_ISL_9163114	EPI_ISL_9161509	EPI_ISL_9163204	EPI_ISL_8658364	EPI_ISL_8783186
EPI_ISL_8831550	EPI_ISL_8975672	EPI_ISL_9266881	EPI_ISL_9202432	EPI_ISL_9202640
EPI_ISL_9202600	EPI_ISL_9202937	EPI_ISL_9402487	EPI_ISL_8481687	EPI_ISL_9161515
EPI_ISL_9161496	EPI_ISL_9196437	EPI_ISL_8716788	EPI_ISL_8837588	EPI_ISL_8893589
EPI_ISL_9202489	EPI_ISL_8785246	EPI_ISL_8799979	EPI_ISL_8799963	EPI_ISL_8658460
EPI_ISL_8658475	EPI_ISL_8785090	EPI_ISL_8519295	EPI_ISL_8783157	EPI_ISL_8837591
EPI_ISL_9179891	EPI_ISL_8476555	EPI_ISL_8807992	EPI_ISL_8540940	EPI_ISL_9021565
EPI_ISL_8837993	EPI_ISL_9267212	EPI_ISL_9266912	EPI_ISL_9267428	EPI_ISL_9267441

EPI_ISL_9267404	EPI_ISL_8625094	EPI_ISL_8591547	EPI_ISL_8589856	EPI_ISL_8530796
EPI_ISL_8712596	EPI_ISL_8658396	EPI_ISL_9290082	EPI_ISL_9290991	EPI_ISL_8568997
EPI_ISL_8837454	EPI_ISL_8837553	EPI_ISL_8837575	EPI_ISL_9154761	EPI_ISL_9202753
EPI_ISL_9509541	EPI_ISL_8589885	EPI_ISL_8764298	EPI_ISL_9267054	EPI_ISL_9706385
EPI_ISL_9712965	EPI_ISL_9161486	EPI_ISL_9981786	EPI_ISL_8925402	EPI_ISL_9706115
EPI_ISL_9748865	EPI_ISL_8476578	EPI_ISL_10088281	EPI_ISL_9179949	EPI_ISL_9977935
EPI_ISL_9352563	EPI_ISL_10014515	EPI_ISL_10088381	EPI_ISL_8837667	EPI_ISL_9418013
EPI_ISL_10088083	EPI_ISL_9985494	EPI_ISL_9985063	EPI_ISL_9699594	EPI_ISL_9352557
EPI_ISL_9357602	EPI_ISL_9357565	EPI_ISL_9356768	EPI_ISL_9352577	EPI_ISL_8770558
EPI_ISL_9375995	EPI_ISL_9713156	EPI_ISL_9705996	EPI_ISL_9376166	EPI_ISL_9376020
EPI_ISL_9375846	EPI_ISL_9375870	EPI_ISL_9376239	EPI_ISL_9375717	EPI_ISL_9376179
EPI_ISL_9432051	EPI_ISL_9985443	EPI_ISL_9985280	EPI_ISL_9202455	EPI_ISL_9712872
EPI_ISL_9687936	EPI_ISL_9748973	EPI_ISL_9375929	EPI_ISL_9379830	EPI_ISL_9392174
EPI_ISL_9392097	EPI_ISL_9392390	EPI_ISL_8569061	EPI_ISL_9392244	EPI_ISL_9392245
EPI_ISL_9392246	EPI_ISL_9392297	EPI_ISL_9392218	EPI_ISL_9392452	EPI_ISL_9392451
EPI_ISL_10114957	EPI_ISL_9392439	EPI_ISL_9402500	EPI_ISL_9402510	EPI_ISL_9959165
EPI_ISL_9820584	EPI_ISL_9854599	EPI_ISL_9589539	EPI_ISL_9706264	EPI_ISL_9431916
EPI_ISL_9432407	EPI_ISL_9432139	EPI_ISL_9431715	EPI_ISL_9431766	EPI_ISL_9432248
EPI_ISL_9432212	EPI_ISL_9431883	EPI_ISL_9431884	EPI_ISL_9431574	EPI_ISL_9432059
EPI_ISL_9432074	EPI_ISL_9111471	EPI_ISL_8716771	EPI_ISL_9534166	EPI_ISL_9830290
EPI_ISL_9985468	EPI_ISL_10088290	EPI_ISL_9978015	EPI_ISL_9854601	EPI_ISL_9859353
EPI_ISL_9706268	EPI_ISL_9492870	EPI_ISL_9492849	EPI_ISL_9492848	EPI_ISL_9161467
EPI_ISL_9512512	EPI_ISL_9506493	EPI_ISL_9503350	EPI_ISL_9509592	EPI_ISL_9509649
EPI_ISL_9512467	EPI_ISL_9512450	EPI_ISL_9508928	EPI_ISL_9508929	EPI_ISL_9509461
EPI_ISL_9509100	EPI_ISL_9509110	EPI_ISL_9509127	EPI_ISL_9509206	EPI_ISL_9509022
EPI_ISL_9515438	EPI_ISL_9512931	EPI_ISL_9512934	EPI_ISL_9514677	EPI_ISL_8591558
EPI_ISL_9534147	EPI_ISL_9534229	EPI_ISL_8589873	EPI_ISL_9589860	EPI_ISL_9589869
EPI_ISL_9614894	EPI_ISL_9614868	EPI_ISL_9614866	EPI_ISL_9614640	EPI_ISL_9614648
EPI_ISL_9614613	EPI_ISL_9612834	EPI_ISL_9618407	EPI_ISL_9627852	EPI_ISL_9627846
EPI_ISL_9627837	EPI_ISL_9650375	EPI_ISL_9650287	EPI_ISL_9651618	EPI_ISL_9392477
EPI_ISL_8838039	EPI_ISL_9658326	EPI_ISL_9658331	EPI_ISL_9658265	EPI_ISL_9658283
EPI_ISL_9673228	EPI_ISL_9673265	EPI_ISL_9673314	EPI_ISL_9673330	EPI_ISL_9673139
EPI_ISL_9673145	EPI_ISL_9673157	EPI_ISL_9673476	EPI_ISL_9673499	EPI_ISL_9673491
EPI_ISL_9673372	EPI_ISL_9673262	EPI_ISL_9673249	EPI_ISL_9673253	EPI_ISL_9673271
EPI_ISL_9673170	EPI_ISL_9673208	EPI_ISL_9687870	EPI_ISL_9111478	EPI_ISL_9687966
EPI_ISL_9687972	EPI_ISL_9687976	EPI_ISL_9687822	EPI_ISL_9687836	EPI_ISL_9687858
EPI_ISL_9431972	EPI_ISL_9688023	EPI_ISL_9688054	EPI_ISL_9688049	EPI_ISL_9688070
EPI_ISL_9688060	EPI_ISL_9699622	EPI_ISL_9699570	EPI_ISL_9699601	EPI_ISL_9267069
EPI_ISL_9706129	EPI_ISL_9706121	EPI_ISL_9706105	EPI_ISL_9706133	EPI_ISL_9706130
EPI_ISL_9706176	EPI_ISL_9706032	EPI_ISL_9706065	EPI_ISL_9706061	EPI_ISL_9706053
EPI_ISL_9706097	EPI_ISL_9706388	EPI_ISL_9706308	EPI_ISL_9706306	EPI_ISL_9705935
EPI_ISL_9705919	EPI_ISL_9705995	EPI_ISL_9705988	EPI_ISL_9705976	EPI_ISL_9705966
EPI_ISL_9706215	EPI_ISL_9706267	EPI_ISL_9706265	EPI_ISL_9705764	EPI_ISL_9705763
EPI_ISL_9705876	EPI_ISL_9712826	EPI_ISL_9712837	EPI_ISL_9712847	EPI_ISL_9713315
EPI_ISL_9712898	EPI_ISL_9712868	EPI_ISL_9712725	EPI_ISL_9713167	EPI_ISL_9713216
EPI_ISL_9712739	EPI_ISL_9713071	EPI_ISL_9713085	EPI_ISL_9713092	EPI_ISL_9713122
EPI_ISL_9712686	EPI_ISL_9713015	EPI_ISL_9713585	EPI_ISL_10014569	EPI_ISL_9713571
EPI_ISL_9713574	EPI_ISL_9713510	EPI_ISL_9712938	EPI_ISL_9712943	EPI_ISL_9712908
EPI_ISL_9713496	EPI_ISL_9712992	EPI_ISL_9718961	EPI_ISL_9718881	EPI_ISL_9687860
EPI_ISL_9725840	EPI_ISL_9725874	EPI_ISL_9725762	EPI_ISL_9725569	EPI_ISL_9725566
EPI_ISL_9907297	EPI_ISL_9749011	EPI_ISL_9534097	EPI_ISL_9748963	EPI_ISL_9748969
EPI_ISL_9748937	EPI_ISL_9753041	EPI_ISL_9753049	EPI_ISL_9753046	EPI_ISL_9753053
EPI_ISL_9754225	EPI_ISL_9752449	EPI_ISL_9752413	EPI_ISL_9754251	EPI_ISL_9754977
EPI_ISL_9754932	EPI_ISL_9754195	EPI_ISL_10122689	EPI_ISL_9508998	EPI_ISL_10122644
EPI_ISL_10014504	EPI_ISL_9791170	EPI_ISL_9791130	EPI_ISL_9791104	EPI_ISL_8851279

EPI_ISL_8925419	EPI_ISL_8928592	EPI_ISL_8582180	EPI_ISL_8481680	EPI_ISL_9664881
EPI_ISL_9844280	EPI_ISL_10115780	EPI_ISL_8625099	EPI_ISL_10115338	EPI_ISL_10114985
EPI_ISL_10114968	EPI_ISL_10114944	EPI_ISL_9418029	EPI_ISL_10119906	EPI_ISL_10122709
EPI_ISL_10122714	EPI_ISL_8672070	EPI_ISL_8609667	EPI_ISL_10140420	EPI_ISL_10140410
EPI_ISL_10138727	EPI_ISL_10138720	EPI_ISL_10138681	EPI_ISL_10138627	EPI_ISL_10136985
EPI_ISL_10136953	EPI_ISL_10136909	EPI_ISL_10134836	EPI_ISL_10122679	EPI_ISL_10120009
EPI_ISL_10119961	EPI_ISL_10119952	EPI_ISL_10119925	EPI_ISL_10115788	EPI_ISL_10115751
EPI_ISL_10115741	EPI_ISL_10115735	EPI_ISL_10115729	EPI_ISL_10114998	EPI_ISL_10114959
EPI_ISL_10058570	EPI_ISL_10049571	EPI_ISL_10049563	EPI_ISL_10014526	EPI_ISL_10014501
EPI_ISL_10014499	EPI_ISL_10014497	EPI_ISL_10008065	EPI_ISL_9981867	EPI_ISL_9981862
EPI_ISL_9981840	EPI_ISL_9981832	EPI_ISL_9981827	EPI_ISL_9981810	EPI_ISL_9981769
EPI_ISL_9981766	EPI_ISL_9981739	EPI_ISL_9981734	EPI_ISL_9981732	EPI_ISL_9976624
EPI_ISL_9976602	EPI_ISL_9976562	EPI_ISL_9976427	EPI_ISL_9915096	EPI_ISL_9915090
EPI_ISL_9907291	EPI_ISL_9859357	EPI_ISL_9854596	EPI_ISL_9854505	EPI_ISL_9854474
EPI_ISL_9854455	EPI_ISL_9854422	EPI_ISL_9854421	EPI_ISL_9854399	EPI_ISL_9822898
EPI_ISL_9822893	EPI_ISL_9791176	EPI_ISL_9791173	EPI_ISL_9786250	EPI_ISL_9778905
EPI_ISL_9771189	EPI_ISL_9771182	EPI_ISL_9754971	EPI_ISL_9754964	EPI_ISL_9754941
EPI_ISL_9754257	EPI_ISL_9754243	EPI_ISL_9752410	EPI_ISL_9725591	EPI_ISL_9694160
EPI_ISL_12750018	EPI_ISL_10505505	EPI_ISL_14052301	EPI_ISL_10142510	EPI_ISL_10254711
EPI_ISL_10256211	EPI_ISL_10254411	EPI_ISL_14505561	EPI_ISL_14605050	EPI_ISL_14605031
EPI_ISL_14605057	EPI_ISL_14301506	EPI_ISL_14301478	EPI_ISL_14301474	EPI_ISL_14301467
EPI_ISL_14301049	EPI_ISL_14301047	EPI_ISL_10255733	EPI_ISL_10438033	EPI_ISL_10256234
EPI_ISL_14935329	EPI_ISL_14935333	EPI_ISL_10219251	EPI_ISL_10242916	EPI_ISL_10142542
EPI_ISL_10219242	EPI_ISL_10255749	EPI_ISL_10254845	EPI_ISL_14253228	EPI_ISL_14253194
EPI_ISL_14253174	EPI_ISL_14253173	EPI_ISL_14253170	EPI_ISL_14253168	EPI_ISL_10505252
EPI_ISL_10564014	EPI_ISL_10403591	EPI_ISL_10403636	EPI_ISL_10406967	EPI_ISL_10588468
EPI_ISL_10590968	EPI_ISL_10565576	EPI_ISL_10219270	EPI_ISL_14214383	EPI_ISL_10254157
EPI_ISL_10254161	EPI_ISL_10254166	EPI_ISL_10254173	EPI_ISL_10254185	EPI_ISL_10254195
EPI_ISL_10254207	EPI_ISL_10254208	EPI_ISL_10254293	EPI_ISL_10254396	EPI_ISL_10254442
EPI_ISL_10254445	EPI_ISL_10254453	EPI_ISL_10254461	EPI_ISL_10254462	EPI_ISL_10254501
EPI_ISL_10254510	EPI_ISL_10254521	EPI_ISL_10254537	EPI_ISL_10254552	EPI_ISL_10254571
EPI_ISL_10254577	EPI_ISL_10254628	EPI_ISL_10254632	EPI_ISL_10254663	EPI_ISL_10254644
EPI_ISL_10254700	EPI_ISL_10254701	EPI_ISL_10254707	EPI_ISL_10254823	EPI_ISL_10254832
EPI_ISL_10254858	EPI_ISL_10254911	EPI_ISL_10254924	EPI_ISL_10254957	EPI_ISL_10254960
EPI_ISL_10254985	EPI_ISL_10254984	EPI_ISL_10255021	EPI_ISL_10255029	EPI_ISL_10255037
EPI_ISL_10255053	EPI_ISL_10255055	EPI_ISL_10255059	EPI_ISL_10255068	EPI_ISL_10255127
EPI_ISL_10256010	EPI_ISL_10256013	EPI_ISL_10256043	EPI_ISL_10256064	EPI_ISL_10256116
EPI_ISL_10256189	EPI_ISL_10256192	EPI_ISL_10256190	EPI_ISL_10256242	EPI_ISL_10256270
EPI_ISL_10256283	EPI_ISL_10256314	EPI_ISL_10256328	EPI_ISL_10256330	EPI_ISL_10256352
EPI_ISL_10256339	EPI_ISL_10256353	EPI_ISL_10256379	EPI_ISL_10256406	EPI_ISL_10256442
EPI_ISL_10256439	EPI_ISL_10256445	EPI_ISL_10256505	EPI_ISL_10256506	EPI_ISL_10256512
EPI_ISL_10256548	EPI_ISL_10256545	EPI_ISL_10255061	EPI_ISL_14199921	EPI_ISL_14199907
EPI_ISL_14199892	EPI_ISL_10402683	EPI_ISL_10402711	EPI_ISL_10402721	EPI_ISL_10402733
EPI_ISL_10505210	EPI_ISL_10505273	EPI_ISL_10505320	EPI_ISL_10505329	EPI_ISL_10505363
EPI_ISL_10505367	EPI_ISL_10505372	EPI_ISL_10505377	EPI_ISL_10505401	EPI_ISL_10505411
EPI_ISL_10505431	EPI_ISL_10505441	EPI_ISL_10505427	EPI_ISL_10505463	EPI_ISL_10505485
EPI_ISL_10505521	EPI_ISL_10505568	EPI_ISL_10505632	EPI_ISL_10505640	EPI_ISL_10505673
EPI_ISL_10505655	EPI_ISL_10505650	EPI_ISL_10505665	EPI_ISL_10505685	EPI_ISL_10505681
EPI_ISL_10505696	EPI_ISL_10505721	EPI_ISL_10505738	EPI_ISL_10505754	EPI_ISL_10505841
EPI_ISL_10505875	EPI_ISL_10505900	EPI_ISL_10629600	EPI_ISL_10719855	EPI_ISL_10719868
EPI_ISL_10719878	EPI_ISL_10719894	EPI_ISL_10719912	EPI_ISL_10719954	EPI_ISL_10719978
EPI_ISL_10719969	EPI_ISL_10719979	EPI_ISL_10719988	EPI_ISL_10719990	EPI_ISL_10720035
EPI_ISL_10720053	EPI_ISL_10720055	EPI_ISL_10720067	EPI_ISL_10720072	EPI_ISL_10720073
EPI_ISL_10720095	EPI_ISL_10720129	EPI_ISL_10720144	EPI_ISL_10720155	EPI_ISL_10720175
EPI_ISL_10720185	EPI_ISL_10720187	EPI_ISL_10720218	EPI_ISL_10720221	EPI_ISL_10720257

EPI_ISL_10720258	EPI_ISL_10720269	EPI_ISL_10720272	EPI_ISL_10720292	EPI_ISL_10720327
EPI_ISL_10720336	EPI_ISL_10720357	EPI_ISL_10720361	EPI_ISL_10720374	EPI_ISL_10720414
EPI_ISL_10720438	EPI_ISL_10720453	EPI_ISL_10720468	EPI_ISL_10720470	EPI_ISL_10720503
EPI_ISL_10720525	EPI_ISL_10720526	EPI_ISL_10720541	EPI_ISL_10720551	EPI_ISL_10720565
EPI_ISL_10720618	EPI_ISL_10720619	EPI_ISL_10720624	EPI_ISL_10720630	EPI_ISL_10720636
EPI_ISL_10720663	EPI_ISL_10720657	EPI_ISL_10720661	EPI_ISL_10720677	EPI_ISL_10720662
EPI_ISL_10720665	EPI_ISL_10720675	EPI_ISL_10720679	EPI_ISL_10720706	EPI_ISL_10728917
EPI_ISL_10728929	EPI_ISL_10728932	EPI_ISL_10728949	EPI_ISL_10728965	EPI_ISL_14174839
EPI_ISL_14174834	EPI_ISL_14174832	EPI_ISL_10847116	EPI_ISL_10847121	EPI_ISL_10847145
EPI_ISL_10847149	EPI_ISL_10847187	EPI_ISL_10847190	EPI_ISL_10847215	EPI_ISL_10847221
EPI_ISL_10847276	EPI_ISL_10847287	EPI_ISL_10847321	EPI_ISL_10847428	EPI_ISL_10847444
EPI_ISL_10847463	EPI_ISL_10847468	EPI_ISL_10847491	EPI_ISL_10847480	EPI_ISL_10847500
EPI_ISL_10847521	EPI_ISL_14145984	EPI_ISL_14145975	EPI_ISL_14145961	EPI_ISL_14145932
EPI_ISL_10957667	EPI_ISL_10957676	EPI_ISL_10957691	EPI_ISL_10957707	EPI_ISL_10981169
EPI_ISL_10981206	EPI_ISL_10981231	EPI_ISL_10981217	EPI_ISL_10981248	EPI_ISL_10981260
EPI_ISL_14351526	EPI_ISL_14093811	EPI_ISL_11234465	EPI_ISL_14071190	EPI_ISL_14071185
EPI_ISL_14071172	EPI_ISL_14070444	EPI_ISL_14070210	EPI_ISL_11414193	EPI_ISL_11414196
EPI_ISL_11414233	EPI_ISL_11414218	EPI_ISL_11414241	EPI_ISL_11414239	EPI_ISL_11414276
EPI_ISL_11414275	EPI_ISL_11414270	EPI_ISL_11439425	EPI_ISL_11439429	EPI_ISL_10494458
EPI_ISL_10505691	EPI_ISL_11933353	EPI_ISL_12736662	EPI_ISL_10590962	EPI_ISL_14426158
EPI_ISL_14426133	EPI_ISL_14426111	EPI_ISL_14665561	EPI_ISL_13985829	EPI_ISL_14392491
EPI_ISL_14389512	EPI_ISL_14389468	EPI_ISL_14355268	EPI_ISL_14355261	EPI_ISL_14353948
EPI_ISL_14353939	EPI_ISL_14353937	EPI_ISL_14353906	EPI_ISL_14353901	EPI_ISL_14351533
EPI_ISL_13970207	EPI_ISL_13970201	EPI_ISL_13970180	EPI_ISL_13970179	EPI_ISL_13970150
EPI_ISL_13949163	EPI_ISL_14751820	EPI_ISL_14751812	EPI_ISL_13907790	EPI_ISL_13046576
EPI_ISL_13891536	EPI_ISL_13891516	EPI_ISL_13891507	EPI_ISL_13891490	EPI_ISL_10497518
EPI_ISL_10497516	EPI_ISL_10497504	EPI_ISL_10497495	EPI_ISL_14660103	EPI_ISL_10505784
EPI_ISL_14660084	EPI_ISL_10505586	EPI_ISL_10564074	EPI_ISL_10255686	EPI_ISL_10254285
EPI_ISL_10271152	EPI_ISL_10255099	EPI_ISL_10256199	EPI_ISL_13540198	EPI_ISL_13540194
EPI_ISL_10494461	EPI_ISL_10494375	EPI_ISL_10494374	EPI_ISL_10256156	EPI_ISL_10343969
EPI_ISL_10505596	EPI_ISL_10256218	EPI_ISL_13444576	EPI_ISL_10406968	EPI_ISL_13351770
EPI_ISL_14549883	EPI_ISL_14549855	EPI_ISL_13243363	EPI_ISL_13243351	EPI_ISL_13180927
EPI_ISL_10255678	EPI_ISL_13157835	EPI_ISL_13157829	EPI_ISL_10202059	EPI_ISL_10438144
EPI_ISL_10142530	EPI_ISL_10176467	EPI_ISL_10406934	EPI_ISL_10505561	EPI_ISL_10449683
EPI_ISL_10449663	EPI_ISL_10449612	EPI_ISL_10449542	EPI_ISL_10449540	EPI_ISL_10438120
EPI_ISL_10438110	EPI_ISL_10438090	EPI_ISL_10438088	EPI_ISL_10438041	EPI_ISL_10438026
EPI_ISL_10437981	EPI_ISL_14715116	EPI_ISL_14715098	EPI_ISL_14976903	EPI_ISL_14976901
EPI_ISL_14976887	EPI_ISL_14976885	EPI_ISL_14976877	EPI_ISL_14917901	EPI_ISL_14917882
EPI_ISL_14917880	EPI_ISL_14917879	EPI_ISL_14917869	EPI_ISL_14917867	EPI_ISL_14917862
EPI_ISL_14917853	EPI_ISL_14915297	EPI_ISL_14915285	EPI_ISL_14885155	EPI_ISL_14885146
EPI_ISL_14885144	EPI_ISL_14885141	EPI_ISL_14885131	EPI_ISL_14885105	EPI_ISL_14822428
EPI_ISL_14822411	EPI_ISL_14822388	EPI_ISL_14822381	EPI_ISL_14794075	EPI_ISL_14774724
EPI_ISL_14733389	EPI_ISL_14733339	EPI_ISL_14733319	EPI_ISL_14733315	EPI_ISL_14702273
EPI_ISL_14702262	EPI_ISL_14702181	EPI_ISL_14665444	EPI_ISL_14625214	EPI_ISL_14625196
EPI_ISL_14625194	EPI_ISL_14625192	EPI_ISL_14625182	EPI_ISL_14625176	EPI_ISL_14625017
EPI_ISL_14625010	EPI_ISL_14625005	EPI_ISL_14624989	EPI_ISL_14624986	EPI_ISL_14585883
EPI_ISL_14585882	EPI_ISL_14585881	EPI_ISL_14585879	EPI_ISL_14573716	EPI_ISL_14573699
EPI_ISL_14573694	EPI_ISL_14573677	EPI_ISL_14573573	EPI_ISL_14573561	EPI_ISL_14573552
EPI_ISL_14549927	EPI_ISL_14549897	EPI_ISL_14549877	EPI_ISL_14549873	EPI_ISL_14549857
EPI_ISL_14549845	EPI_ISL_14547913	EPI_ISL_14547910	EPI_ISL_14547903	EPI_ISL_14547893
EPI_ISL_14478640	EPI_ISL_14478637	EPI_ISL_14478636	EPI_ISL_14478615	EPI_ISL_14478611
EPI_ISL_14478609	EPI_ISL_14478582	EPI_ISL_14435434	EPI_ISL_14435427	EPI_ISL_14433852
EPI_ISL_14431700	EPI_ISL_14431691	EPI_ISL_13001288	EPI_ISL_12897414	EPI_ISL_12897411
EPI_ISL_12812495	EPI_ISL_12812455	EPI_ISL_12812413	EPI_ISL_12812412	EPI_ISL_12812397
EPI_ISL_12812327	EPI_ISL_12750028	EPI_ISL_12658032	EPI_ISL_12648152	EPI_ISL_12631946

EPI_ISL_12615313	EPI_ISL_12615298	EPI_ISL_12615297	EPI_ISL_12615288	EPI_ISL_12615278
EPI_ISL_12615270	EPI_ISL_12615265	EPI_ISL_12615223	EPI_ISL_12615210	EPI_ISL_12615192
EPI_ISL_12615185	EPI_ISL_12615171	EPI_ISL_12615162	EPI_ISL_12615153	EPI_ISL_12615145
EPI_ISL_12615139	EPI_ISL_12615138	EPI_ISL_12615127	EPI_ISL_12615119	EPI_ISL_12615089
EPI_ISL_12615083	EPI_ISL_12615065	EPI_ISL_12615064	EPI_ISL_12615025	EPI_ISL_12614999
EPI_ISL_12614980	EPI_ISL_12614979	EPI_ISL_12614942	EPI_ISL_12614910	EPI_ISL_12614897
EPI_ISL_12614884	EPI_ISL_12614876	EPI_ISL_12614849	EPI_ISL_12614837	EPI_ISL_12614820
EPI_ISL_12614813	EPI_ISL_12614808	EPI_ISL_12614764	EPI_ISL_12614660	EPI_ISL_12614602
EPI_ISL_12614592	EPI_ISL_12614591	EPI_ISL_12614590	EPI_ISL_12614584	EPI_ISL_12614582
EPI_ISL_12614561	EPI_ISL_12614534	EPI_ISL_12614528	EPI_ISL_12614500	EPI_ISL_12614486
EPI_ISL_12614477	EPI_ISL_12614475	EPI_ISL_12614410	EPI_ISL_12614406	EPI_ISL_12614402
EPI_ISL_12614399	EPI_ISL_12614348	EPI_ISL_12614337	EPI_ISL_12614324	EPI_ISL_12614320
EPI_ISL_12614312	EPI_ISL_12614289	EPI_ISL_12614283	EPI_ISL_12588301	EPI_ISL_12575932
EPI_ISL_12559633	EPI_ISL_12559630	EPI_ISL_12559625	EPI_ISL_12559605	EPI_ISL_12559600
EPI_ISL_12538623	EPI_ISL_12292412	EPI_ISL_12253981	EPI_ISL_12253976	EPI_ISL_12179997
EPI_ISL_12178995	EPI_ISL_12177473	EPI_ISL_12155768	EPI_ISL_12124267	EPI_ISL_12124245
EPI_ISL_12124238	EPI_ISL_12124221	EPI_ISL_12124046	EPI_ISL_12124029	EPI_ISL_12124022
EPI_ISL_12123984	EPI_ISL_12055202	EPI_ISL_12055194	EPI_ISL_12055186	EPI_ISL_12055155
EPI_ISL_12055140	EPI_ISL_12055133	EPI_ISL_12055068	EPI_ISL_12054970	EPI_ISL_12054948
EPI_ISL_12054942	EPI_ISL_12054938	EPI_ISL_12011507	EPI_ISL_12011470	EPI_ISL_12008580
EPI_ISL_12008560	EPI_ISL_12008555	EPI_ISL_12008554	EPI_ISL_12008553	EPI_ISL_12008543
EPI_ISL_12008052	EPI_ISL_12007761	EPI_ISL_11991658	EPI_ISL_11905936	EPI_ISL_11905930
EPI_ISL_11881933	EPI_ISL_11881719	EPI_ISL_11881713	EPI_ISL_11881479	EPI_ISL_11841804
EPI_ISL_11841803	EPI_ISL_11841796	EPI_ISL_11841787	EPI_ISL_11841779	EPI_ISL_11841775
EPI_ISL_11841767	EPI_ISL_11841765	EPI_ISL_11832051	EPI_ISL_11832043	EPI_ISL_11832040
EPI_ISL_11832038	EPI_ISL_11832035	EPI_ISL_11832028	EPI_ISL_11832021	EPI_ISL_11831862
EPI_ISL_11831838	EPI_ISL_11814171	EPI_ISL_11814167	EPI_ISL_11814141	EPI_ISL_11814127
EPI_ISL_11814123	EPI_ISL_11814120	EPI_ISL_11814114	EPI_ISL_11814112	EPI_ISL_11814109
EPI_ISL_11814104	EPI_ISL_11809490	EPI_ISL_11780719	EPI_ISL_11780695	EPI_ISL_11780692
EPI_ISL_11780689	EPI_ISL_11780677	EPI_ISL_11780658	EPI_ISL_11780655	EPI_ISL_11780645
EPI_ISL_11750199	EPI_ISL_11750197	EPI_ISL_11750164	EPI_ISL_11749613	EPI_ISL_11749605
EPI_ISL_11737405	EPI_ISL_11737390	EPI_ISL_11737387	EPI_ISL_11737386	EPI_ISL_11737351
EPI_ISL_11737345	EPI_ISL_11737343	EPI_ISL_11737340	EPI_ISL_11737326	EPI_ISL_11737322
EPI_ISL_11670744	EPI_ISL_11664465	EPI_ISL_11664464	EPI_ISL_11664462	EPI_ISL_11664452
EPI_ISL_11629378	EPI_ISL_11610675	EPI_ISL_11610669	EPI_ISL_11586782	EPI_ISL_11586780
EPI_ISL_11586771	EPI_ISL_11586757	EPI_ISL_11586754	EPI_ISL_11586753	EPI_ISL_11580693
EPI_ISL_11580688	EPI_ISL_11580676	EPI_ISL_11580675	EPI_ISL_11580673	EPI_ISL_11580670
EPI_ISL_11580669	EPI_ISL_11580666	EPI_ISL_11580648	EPI_ISL_11580432	EPI_ISL_11580406
EPI_ISL_11580405	EPI_ISL_11580395	EPI_ISL_11580385	EPI_ISL_11580383	EPI_ISL_11580365
EPI_ISL_11580363	EPI_ISL_11522906	EPI_ISL_11522903	EPI_ISL_11522895	EPI_ISL_11522864
EPI_ISL_11522853	EPI_ISL_11522844	EPI_ISL_11522830	EPI_ISL_11521942	EPI_ISL_11521917
EPI_ISL_11521887	EPI_ISL_11521862	EPI_ISL_11449754	EPI_ISL_11449753	EPI_ISL_11449336
EPI_ISL_11449327	EPI_ISL_11449315	EPI_ISL_11449305	EPI_ISL_11449293	EPI_ISL_11449288
EPI_ISL_11449278	EPI_ISL_11449265	EPI_ISL_11449250	EPI_ISL_11441148	EPI_ISL_11381983
EPI_ISL_11371486	EPI_ISL_11371473	EPI_ISL_11370986	EPI_ISL_11370968	EPI_ISL_11370967
EPI_ISL_11370962	EPI_ISL_11370955	EPI_ISL_11370947	EPI_ISL_11370945	EPI_ISL_11370940
EPI_ISL_11355861	EPI_ISL_11349028	EPI_ISL_11349027	EPI_ISL_11348672	EPI_ISL_11323436
EPI_ISL_11325250	EPI_ISL_11325237	EPI_ISL_11325231	EPI_ISL_11325200	EPI_ISL_11325196
EPI_ISL_11325183	EPI_ISL_11325179	EPI_ISL_11325174	EPI_ISL_11324753	EPI_ISL_11323473
EPI_ISL_11323460	EPI_ISL_11323458	EPI_ISL_11323452	EPI_ISL_11323427	EPI_ISL_11322016
EPI_ISL_11321670	EPI_ISL_11321663	EPI_ISL_11321645	EPI_ISL_11321593	EPI_ISL_11321586
EPI_ISL_11321581	EPI_ISL_11315486	EPI_ISL_11315481	EPI_ISL_11312754	EPI_ISL_11312752
EPI_ISL_11312750	EPI_ISL_11312738	EPI_ISL_11312732	EPI_ISL_11312648	EPI_ISL_11290341
EPI_ISL_11290338	EPI_ISL_11290337	EPI_ISL_11290336	EPI_ISL_11290331	EPI_ISL_11290305
EPI_ISL_11290285	EPI_ISL_11290271	EPI_ISL_11290264	EPI_ISL_11275660	EPI_ISL_11268025

EPI_ISL_11268024	EPI_ISL_11268017	EPI_ISL_11222402	EPI_ISL_11222375	EPI_ISL_11166335
EPI_ISL_11166310	EPI_ISL_11166304	EPI_ISL_11166279	EPI_ISL_11166273	EPI_ISL_11162573
EPI_ISL_11162561	EPI_ISL_11162560	EPI_ISL_11160439	EPI_ISL_11160435	EPI_ISL_11160233
EPI_ISL_11148051	EPI_ISL_11147956	EPI_ISL_11147935	EPI_ISL_11116158	EPI_ISL_11116155
EPI_ISL_11110120	EPI_ISL_11110112	EPI_ISL_11109900	EPI_ISL_11109897	EPI_ISL_11109891
EPI_ISL_11109870	EPI_ISL_11109866	EPI_ISL_11109839	EPI_ISL_11109810	EPI_ISL_11109794
EPI_ISL_11109520	EPI_ISL_11109515	EPI_ISL_11109509	EPI_ISL_11109506	EPI_ISL_11073018
EPI_ISL_11073006	EPI_ISL_11073004	EPI_ISL_11072598	EPI_ISL_11070405	EPI_ISL_11070354
EPI_ISL_11070343	EPI_ISL_11070333	EPI_ISL_11070306	EPI_ISL_11070305	EPI_ISL_11070292
EPI_ISL_11070283	EPI_ISL_11070273	EPI_ISL_11070272	EPI_ISL_11070271	EPI_ISL_11068145
EPI_ISL_11068142	EPI_ISL_11068133	EPI_ISL_11068125	EPI_ISL_11068116	EPI_ISL_11068096
EPI_ISL_11068092	EPI_ISL_11068089	EPI_ISL_11068086	EPI_ISL_11068079	EPI_ISL_11067776
EPI_ISL_11067769	EPI_ISL_11067759	EPI_ISL_11067757	EPI_ISL_11058850	EPI_ISL_11055591
EPI_ISL_11055588	EPI_ISL_11055582	EPI_ISL_11055565	EPI_ISL_11055560	EPI_ISL_11055555
EPI_ISL_10942771	EPI_ISL_10940712	EPI_ISL_10942760	EPI_ISL_10942756	EPI_ISL_10940754
EPI_ISL_10940750	EPI_ISL_10940740	EPI_ISL_10940721	EPI_ISL_10908581	EPI_ISL_10898265
EPI_ISL_10898254	EPI_ISL_10898252	EPI_ISL_10898187	EPI_ISL_10898149	EPI_ISL_10898134
EPI_ISL_10880024	EPI_ISL_10879993	EPI_ISL_10879982	EPI_ISL_10879975	EPI_ISL_10879962
EPI_ISL_10879930	EPI_ISL_10879761	EPI_ISL_10879738	EPI_ISL_10879732	EPI_ISL_10879703
EPI_ISL_10866638	EPI_ISL_10866632	EPI_ISL_10866610	EPI_ISL_10866600	EPI_ISL_10866599
EPI_ISL_10866577	EPI_ISL_10864894	EPI_ISL_10864793	EPI_ISL_10864777	EPI_ISL_10864775
EPI_ISL_10864770	EPI_ISL_10864766	EPI_ISL_10863380	EPI_ISL_10863379	EPI_ISL_10863378
EPI_ISL_10850609	EPI_ISL_10850589	EPI_ISL_10847054	EPI_ISL_10847045	EPI_ISL_10847031
EPI_ISL_10847028	EPI_ISL_10847015	EPI_ISL_10846984	EPI_ISL_10846981	EPI_ISL_10817050
EPI_ISL_10817004	EPI_ISL_10816975	EPI_ISL_10816972	EPI_ISL_10816918	EPI_ISL_10809885
EPI_ISL_10809879	EPI_ISL_10809872	EPI_ISL_10739062	EPI_ISL_10739054	EPI_ISL_10739046
EPI_ISL_10738802	EPI_ISL_10738783	EPI_ISL_10738085	EPI_ISL_10738061	EPI_ISL_10738042
EPI_ISL_10737990	EPI_ISL_10737960	EPI_ISL_10737927	EPI_ISL_10719932	EPI_ISL_10717328
EPI_ISL_10717319	EPI_ISL_10707458	EPI_ISL_10707453	EPI_ISL_10707447	EPI_ISL_10707424
EPI_ISL_10707422	EPI_ISL_10707421	EPI_ISL_10703620	EPI_ISL_10694386	EPI_ISL_10694384
EPI_ISL_10694379	EPI_ISL_10694349	EPI_ISL_10694345	EPI_ISL_10694297	EPI_ISL_10694246
EPI_ISL_10694216	EPI_ISL_10693905	EPI_ISL_10693861	EPI_ISL_10693759	EPI_ISL_10693656
EPI_ISL_10687280	EPI_ISL_10687276	EPI_ISL_10687223	EPI_ISL_10687220	EPI_ISL_10687219
EPI_ISL_10687083	EPI_ISL_10687051	EPI_ISL_10687012	EPI_ISL_10687011	EPI_ISL_10681495
EPI_ISL_10681481	EPI_ISL_10681450	EPI_ISL_10681447	EPI_ISL_10681439	EPI_ISL_10681431
EPI_ISL_10680776	EPI_ISL_10680733	EPI_ISL_10680725	EPI_ISL_10680711	EPI_ISL_10680652
EPI_ISL_10680632	EPI_ISL_10680631	EPI_ISL_10663275	EPI_ISL_10663241	EPI_ISL_10656861
EPI_ISL_10656856	EPI_ISL_10656823	EPI_ISL_10645421	EPI_ISL_10639846	EPI_ISL_10639799
EPI_ISL_10639798	EPI_ISL_10623446	EPI_ISL_10623439	EPI_ISL_10591842	EPI_ISL_10590984
EPI_ISL_10590964	EPI_ISL_10590953	EPI_ISL_10590931	EPI_ISL_10590911	EPI_ISL_10588476
EPI_ISL_10588459	EPI_ISL_10565596	EPI_ISL_10565593	EPI_ISL_10565591	EPI_ISL_10565577
EPI_ISL_10565573	EPI_ISL_10564092	EPI_ISL_10564077	EPI_ISL_10564059	EPI_ISL_10564044
EPI_ISL_10564043	EPI_ISL_10564039	EPI_ISL_10564034	EPI_ISL_10564029	EPI_ISL_10564023
EPI_ISL_10564020	EPI_ISL_10498157	EPI_ISL_10498145	EPI_ISL_10498135	EPI_ISL_10498113
EPI_ISL_10406971	EPI_ISL_10406965	EPI_ISL_10406941	EPI_ISL_10403762	EPI_ISL_10403740
EPI_ISL_10403729	EPI_ISL_10403720	EPI_ISL_10403695	EPI_ISL_10403678	EPI_ISL_10403674
EPI_ISL_10403592	EPI_ISL_10357006	EPI_ISL_10357002	EPI_ISL_10356986	EPI_ISL_10355449
EPI_ISL_10355437	EPI_ISL_10341734	EPI_ISL_10341733	EPI_ISL_10341711	EPI_ISL_10341710
EPI_ISL_10271197	EPI_ISL_10271194	EPI_ISL_10271135	EPI_ISL_10271131	EPI_ISL_10255752
EPI_ISL_10255732	EPI_ISL_10255721	EPI_ISL_10255709	EPI_ISL_10255702	EPI_ISL_10255701
EPI_ISL_10255691	EPI_ISL_10255330	EPI_ISL_10242989	EPI_ISL_10242900	EPI_ISL_10231931
EPI_ISL_10231912	EPI_ISL_10231738	EPI_ISL_10230070	EPI_ISL_10230046	EPI_ISL_10230037
EPI_ISL_10230029	EPI_ISL_10219320	EPI_ISL_10219315	EPI_ISL_10219312	EPI_ISL_10219308
EPI_ISL_10219294	EPI_ISL_10219291	EPI_ISL_10219278	EPI_ISL_10219262	EPI_ISL_10202149
EPI_ISL_10202097	EPI_ISL_10202072	EPI_ISL_10201800	EPI_ISL_10201761	EPI_ISL_10201716

EPI_ISL_10201700	EPI_ISL_10197664	EPI_ISL_10197648	EPI_ISL_10191773	EPI_ISL_10191742
EPI_ISL_10191739	EPI_ISL_10191731	EPI_ISL_10191720	EPI_ISL_10191711	EPI_ISL_10176456
EPI_ISL_10176452	EPI_ISL_10176354	EPI_ISL_10142350	EPI_ISL_10140467	EPI_ISL_14976907
EPI_ISL_14976915	EPI_ISL_14976940	EPI_ISL_14976945	EPI_ISL_14997159	EPI_ISL_14997166
EPI_ISL_15038730	EPI_ISL_15038734	EPI_ISL_15038740	EPI_ISL_15038743	EPI_ISL_15039588
EPI_ISL_15039613	EPI_ISL_15174175	EPI_ISL_15174186	EPI_ISL_15174187	EPI_ISL_15174188
EPI_ISL_15174189	EPI_ISL_15174202	EPI_ISL_15174204	EPI_ISL_15174209	EPI_ISL_15174212
EPI_ISL_15174241	EPI_ISL_15174245	EPI_ISL_16815648	EPI_ISL_14845077	EPI_ISL_14845078
EPI_ISL_14845079	EPI_ISL_14845080	EPI_ISL_14845081	EPI_ISL_14845082	EPI_ISL_14845083
EPI_ISL_14845084	EPI_ISL_14845085	EPI_ISL_14845086	EPI_ISL_14845087	EPI_ISL_14845088
EPI_ISL_14845089	EPI_ISL_14845090	EPI_ISL_14845091	EPI_ISL_14845092	EPI_ISL_14845093
EPI_ISL_14845094	EPI_ISL_14845095	EPI_ISL_14845096	EPI_ISL_14845097	EPI_ISL_14845098
EPI_ISL_14845099	EPI_ISL_14845100	EPI_ISL_14845101	EPI_ISL_14845102	EPI_ISL_14845103
EPI_ISL_14845104	EPI_ISL_14845105	EPI_ISL_14845106	EPI_ISL_14845107	EPI_ISL_14845108
EPI_ISL_14845109	EPI_ISL_14845110	EPI_ISL_14845111	EPI_ISL_14845112	EPI_ISL_14845113
EPI_ISL_14845114	EPI_ISL_14845115	EPI_ISL_14845116	EPI_ISL_14845117	EPI_ISL_14845118
EPI_ISL_14845119	EPI_ISL_14845120	EPI_ISL_14845121	EPI_ISL_14845122	EPI_ISL_14845123
EPI_ISL_14845124	EPI_ISL_14845125	EPI_ISL_14845126	EPI_ISL_14845127	EPI_ISL_14845128
EPI_ISL_14845129	EPI_ISL_14845130	EPI_ISL_14845131	EPI_ISL_14845132	EPI_ISL_14845133
EPI_ISL_14845134	EPI_ISL_14845135	EPI_ISL_14845136	EPI_ISL_14845137	EPI_ISL_14845138
EPI_ISL_14845139	EPI_ISL_14845140	EPI_ISL_14845141	EPI_ISL_14845142	EPI_ISL_14845143
EPI_ISL_14845144	EPI_ISL_14845145	EPI_ISL_14845146	EPI_ISL_14845147	EPI_ISL_14845148
EPI_ISL_14845149	EPI_ISL_14845150	EPI_ISL_14845151	EPI_ISL_14845152	EPI_ISL_14845153
EPI_ISL_14845154	EPI_ISL_14845155	EPI_ISL_14845156	EPI_ISL_14845157	EPI_ISL_14845158
EPI_ISL_14845159	EPI_ISL_14845160	EPI_ISL_14845161	EPI_ISL_14845162	EPI_ISL_14845163
EPI_ISL_14845164	EPI_ISL_14845165	EPI_ISL_14845166	EPI_ISL_14845167	EPI_ISL_14845168
EPI_ISL_14845169	EPI_ISL_14845170	EPI_ISL_14845171	EPI_ISL_14845172	EPI_ISL_14845173
EPI_ISL_14845174	EPI_ISL_14845175	EPI_ISL_14845176	EPI_ISL_14845177	EPI_ISL_14845178
EPI_ISL_14845179	EPI_ISL_14845180	EPI_ISL_14845181	EPI_ISL_14845182	EPI_ISL_14845183
EPI_ISL_14845184	EPI_ISL_14845185	EPI_ISL_14845186	EPI_ISL_14845187	EPI_ISL_14845188
EPI_ISL_14845189	EPI_ISL_14845190	EPI_ISL_14845191	EPI_ISL_14845192	EPI_ISL_14845193
EPI_ISL_14845194	EPI_ISL_14845195	EPI_ISL_14845196	EPI_ISL_14845197	EPI_ISL_14845198
EPI_ISL_14845199	EPI_ISL_14845200	EPI_ISL_14845201	EPI_ISL_14845202	EPI_ISL_14845203
EPI_ISL_14845204	EPI_ISL_14845205	EPI_ISL_14845206	EPI_ISL_14845207	EPI_ISL_14845208
EPI_ISL_14845209	EPI_ISL_14845210	EPI_ISL_14845211	EPI_ISL_14845212	EPI_ISL_14845213
EPI_ISL_14845214	EPI_ISL_14845215	EPI_ISL_14845216	EPI_ISL_14845217	EPI_ISL_14845218
EPI_ISL_14845219	EPI_ISL_14845220	EPI_ISL_14845221	EPI_ISL_14845222	EPI_ISL_14845223
EPI_ISL_14845224	EPI_ISL_14845225	EPI_ISL_14845226	EPI_ISL_14845227	EPI_ISL_14845228
EPI_ISL_14845229	EPI_ISL_14845230	EPI_ISL_14845231	EPI_ISL_14845232	EPI_ISL_14845233
EPI_ISL_14845234	EPI_ISL_14845235	EPI_ISL_14845236	EPI_ISL_14845237	EPI_ISL_14845238
EPI_ISL_14845239	EPI_ISL_14845240	EPI_ISL_14845241	EPI_ISL_14845242	EPI_ISL_14845243
EPI_ISL_14845244	EPI_ISL_14845245	EPI_ISL_14845246	EPI_ISL_14845247	EPI_ISL_14845248
EPI_ISL_14845249	EPI_ISL_14845250	EPI_ISL_14845251	EPI_ISL_14845252	EPI_ISL_14845253
EPI_ISL_14845254	EPI_ISL_14845255	EPI_ISL_14845256	EPI_ISL_14845257	EPI_ISL_14845258
EPI_ISL_14845259	EPI_ISL_14845260	EPI_ISL_14845261	EPI_ISL_14845262	EPI_ISL_14845263
EPI_ISL_14845264	EPI_ISL_14845265	EPI_ISL_14845266	EPI_ISL_14845267	EPI_ISL_14845268
EPI_ISL_14845269	EPI_ISL_14845270	EPI_ISL_14845271	EPI_ISL_14845272	EPI_ISL_14845273
EPI_ISL_14845274	EPI_ISL_14845275	EPI_ISL_14845276	EPI_ISL_14845277	EPI_ISL_14845278
EPI_ISL_14845279	EPI_ISL_14845280	EPI_ISL_14845281	EPI_ISL_14845282	EPI_ISL_14845283
EPI_ISL_14845284	EPI_ISL_14845285	EPI_ISL_14845286	EPI_ISL_14845287	EPI_ISL_14845288
EPI_ISL_14845289	EPI_ISL_14845290	EPI_ISL_14845291	EPI_ISL_14845292	EPI_ISL_14845293
EPI_ISL_14845294	EPI_ISL_14845295	EPI_ISL_14845296	EPI_ISL_14845297	EPI_ISL_14845298
EPI_ISL_14845299	EPI_ISL_14845300	EPI_ISL_14845301	EPI_ISL_14845302	EPI_ISL_14845303
EPI_ISL_14845304	EPI_ISL_14845305	EPI_ISL_14845306	EPI_ISL_14845307	EPI_ISL_14845308
EPI_ISL_14845309	EPI_ISL_14845310	EPI_ISL_14845311	EPI_ISL_14845312	EPI_ISL_14845313

EPI_ISL_14845314	EPI_ISL_14845315	EPI_ISL_14845316	EPI_ISL_14845317	EPI_ISL_14845318
EPI_ISL_14845319	EPI_ISL_14845320	EPI_ISL_14845321	EPI_ISL_14845322	EPI_ISL_14845323
EPI_ISL_14845324	EPI_ISL_14845325	EPI_ISL_14845326	EPI_ISL_14845327	EPI_ISL_14845328
EPI_ISL_14845329	EPI_ISL_14845330	EPI_ISL_14845331	EPI_ISL_14845332	EPI_ISL_14845333
EPI_ISL_14845334	EPI_ISL_14845335	EPI_ISL_14845336	EPI_ISL_14845337	EPI_ISL_14845338
EPI_ISL_14845339	EPI_ISL_14845340	EPI_ISL_14845341	EPI_ISL_14845342	EPI_ISL_14845343
EPI_ISL_14845344	EPI_ISL_14845345	EPI_ISL_14845346	EPI_ISL_14845347	EPI_ISL_14845348
EPI_ISL_14845349	EPI_ISL_14845350	EPI_ISL_14845351	EPI_ISL_14845352	EPI_ISL_14845353
EPI_ISL_14845354	EPI_ISL_14845355	EPI_ISL_14845356	EPI_ISL_14845357	EPI_ISL_14845358
EPI_ISL_14845359	EPI_ISL_14845360	EPI_ISL_14845361	EPI_ISL_14845362	EPI_ISL_14845363
EPI_ISL_14845364	EPI_ISL_14845365	EPI_ISL_14845366	EPI_ISL_14845367	EPI_ISL_14845368
EPI_ISL_14845369	EPI_ISL_14845370	EPI_ISL_14845371	EPI_ISL_14845372	EPI_ISL_14845373
EPI_ISL_14845374	EPI_ISL_14845375	EPI_ISL_14845376	EPI_ISL_14845377	EPI_ISL_14845378
EPI_ISL_14845379	EPI_ISL_14845380	EPI_ISL_14845381	EPI_ISL_14845382	EPI_ISL_14845383
EPI_ISL_14845384	EPI_ISL_14845385	EPI_ISL_14845386	EPI_ISL_14845387	EPI_ISL_14845388
EPI_ISL_14845389	EPI_ISL_14845390	EPI_ISL_14845391	EPI_ISL_14845392	EPI_ISL_14845393
EPI_ISL_14845394	EPI_ISL_14845395	EPI_ISL_14845396	EPI_ISL_14845397	EPI_ISL_14845398
EPI_ISL_14845399	EPI_ISL_14845400	EPI_ISL_14845401	EPI_ISL_14845402	EPI_ISL_14845403
EPI_ISL_14845404	EPI_ISL_14845405	EPI_ISL_14845406	EPI_ISL_14845407	EPI_ISL_14845408
EPI_ISL_14845409	EPI_ISL_14845410	EPI_ISL_14845411	EPI_ISL_14845412	EPI_ISL_14845413
EPI_ISL_14845414	EPI_ISL_14845415	EPI_ISL_14845416	EPI_ISL_14845417	EPI_ISL_14845418
EPI_ISL_14845419	EPI_ISL_14845420	EPI_ISL_14845421	EPI_ISL_14845422	EPI_ISL_14845423
EPI_ISL_14845424	EPI_ISL_14845425	EPI_ISL_14845426	EPI_ISL_14845427	EPI_ISL_14845428
EPI_ISL_14845429	EPI_ISL_14845430	EPI_ISL_14845431	EPI_ISL_14845432	EPI_ISL_14845433
EPI_ISL_14845434	EPI_ISL_14845435	EPI_ISL_14845436	EPI_ISL_14845437	EPI_ISL_14845438
EPI_ISL_14845439	EPI_ISL_14845440	EPI_ISL_14845441	EPI_ISL_14845442	EPI_ISL_14845443
EPI_ISL_14845444	EPI_ISL_6693848	EPI_ISL_6693849	EPI_ISL_6693850	EPI_ISL_6693851
EPI_ISL_6693852	EPI_ISL_6693853	EPI_ISL_6693854	EPI_ISL_6693855	EPI_ISL_6693856
EPI_ISL_6693857	EPI_ISL_6693858	EPI_ISL_6693859	EPI_ISL_6693860	EPI_ISL_6693861
EPI_ISL_6693862	EPI_ISL_6693863	EPI_ISL_6693864	EPI_ISL_6693865	EPI_ISL_6693866
EPI_ISL_6693867	EPI_ISL_6693868	EPI_ISL_6693869	EPI_ISL_6693870	EPI_ISL_6693871
EPI_ISL_6693872	EPI_ISL_6693873	EPI_ISL_6693874	EPI_ISL_6693875	EPI_ISL_6693876
EPI_ISL_6693877	EPI_ISL_6693878	EPI_ISL_6693879	EPI_ISL_6693880	EPI_ISL_6693881
EPI_ISL_6693882	EPI_ISL_6693883	EPI_ISL_6693884	EPI_ISL_6693885	EPI_ISL_6693886
EPI_ISL_6693887	EPI_ISL_6693888	EPI_ISL_6693889	EPI_ISL_6693890	EPI_ISL_6693891
EPI_ISL_6693892	EPI_ISL_6693893	EPI_ISL_6693894	EPI_ISL_6693895	EPI_ISL_6693896
EPI_ISL_6693897	EPI_ISL_6693898	EPI_ISL_6693899	EPI_ISL_6693900	EPI_ISL_6693901
EPI_ISL_6693902	EPI_ISL_6693903	EPI_ISL_6693904	EPI_ISL_6693905	EPI_ISL_6693906
EPI_ISL_6693907	EPI_ISL_6693908	EPI_ISL_6693909	EPI_ISL_6693910	EPI_ISL_6693911
EPI_ISL_6693912	EPI_ISL_6693913	EPI_ISL_6693914	EPI_ISL_6693915	EPI_ISL_6693916
EPI_ISL_6693917	EPI_ISL_6693918	EPI_ISL_6693919	EPI_ISL_6693920	EPI_ISL_6693921
EPI_ISL_6693922	EPI_ISL_6693923	EPI_ISL_6693924	EPI_ISL_6693925	EPI_ISL_6693926
EPI_ISL_6693927	EPI_ISL_6693928	EPI_ISL_6693929	EPI_ISL_6693930	EPI_ISL_6693931
EPI_ISL_6693932	EPI_ISL_6693933	EPI_ISL_6693934	EPI_ISL_6693935	EPI_ISL_6693936
EPI_ISL_6693937	EPI_ISL_6693938	EPI_ISL_6693939	EPI_ISL_6693940	EPI_ISL_6693941
EPI_ISL_6693942	EPI_ISL_6693943	EPI_ISL_6693944	EPI_ISL_6693945	EPI_ISL_6693946
EPI_ISL_6693947	EPI_ISL_6693948	EPI_ISL_6693949	EPI_ISL_6693950	EPI_ISL_6693951
EPI_ISL_6693952	EPI_ISL_6693953	EPI_ISL_6693954	EPI_ISL_6693955	EPI_ISL_6693956
EPI_ISL_6693957	EPI_ISL_6693958	EPI_ISL_6693959	EPI_ISL_6693960	EPI_ISL_6693961
EPI_ISL_6693962	EPI_ISL_6693963	EPI_ISL_6693964	EPI_ISL_6693965	EPI_ISL_6693966
EPI_ISL_6693967	EPI_ISL_6693968	EPI_ISL_6693969	EPI_ISL_6693970	EPI_ISL_6693971
EPI_ISL_6693972	EPI_ISL_6693973	EPI_ISL_6693974	EPI_ISL_6693975	EPI_ISL_6693976
EPI_ISL_6693977	EPI_ISL_6693978	EPI_ISL_6693979	EPI_ISL_6693980	EPI_ISL_6693981
EPI_ISL_6693982	EPI_ISL_6693983	EPI_ISL_6693984	EPI_ISL_6693985	EPI_ISL_6693986
EPI_ISL_6693987	EPI_ISL_6693988	EPI_ISL_6693989	EPI_ISL_6693990	EPI_ISL_6693991

EPI_ISL_6693992	EPI_ISL_6693993	EPI_ISL_6693994	EPI_ISL_6693995	EPI_ISL_6693996
EPI_ISL_6693997	EPI_ISL_6693998	EPI_ISL_6693999	EPI_ISL_6694000	EPI_ISL_6694001
EPI_ISL_6694002	EPI_ISL_6694003	EPI_ISL_6694004	EPI_ISL_6694005	EPI_ISL_6694006
EPI_ISL_6694007	EPI_ISL_6694008	EPI_ISL_6694009	EPI_ISL_6694010	EPI_ISL_6694011
EPI_ISL_6694012	EPI_ISL_6694013	EPI_ISL_6694014	EPI_ISL_6694015	EPI_ISL_6694016
EPI_ISL_6694017	EPI_ISL_6694018	EPI_ISL_6694019	EPI_ISL_6694020	EPI_ISL_6694021
EPI_ISL_6694022	EPI_ISL_6694023	EPI_ISL_6694024	EPI_ISL_6694025	EPI_ISL_6694026
EPI_ISL_6694027	EPI_ISL_7773989	EPI_ISL_7970442	EPI_ISL_7970443	EPI_ISL_7970444
EPI_ISL_7970445	EPI_ISL_7970446	EPI_ISL_7970447	EPI_ISL_7970448	EPI_ISL_7970449
EPI_ISL_7970450	EPI_ISL_7970451	EPI_ISL_7970452	EPI_ISL_7970454	EPI_ISL_7970455
EPI_ISL_7970456	EPI_ISL_7970457	EPI_ISL_7970458	EPI_ISL_7970459	EPI_ISL_7970460
EPI_ISL_7970461	EPI_ISL_7970462	EPI_ISL_7970463	EPI_ISL_7970464	EPI_ISL_7970465
EPI_ISL_7970466	EPI_ISL_7970467	EPI_ISL_7970468	EPI_ISL_7970469	EPI_ISL_7970470
EPI_ISL_7970471	EPI_ISL_7970472	EPI_ISL_7970473	EPI_ISL_7970474	EPI_ISL_7970475
EPI_ISL_7970476	EPI_ISL_7970477	EPI_ISL_7970478	EPI_ISL_7970479	EPI_ISL_7970480
EPI_ISL_7970481	EPI_ISL_7970482	EPI_ISL_7970483	EPI_ISL_7970484	EPI_ISL_7970485
EPI_ISL_7970486	EPI_ISL_7970487	EPI_ISL_7970488	EPI_ISL_7970489	EPI_ISL_7970490
EPI_ISL_7970491	EPI_ISL_7970492	EPI_ISL_7970493	EPI_ISL_7970494	EPI_ISL_7970495
EPI_ISL_7970496	EPI_ISL_7970497	EPI_ISL_7970498	EPI_ISL_7970499	EPI_ISL_7970500
EPI_ISL_7970501	EPI_ISL_7970502	EPI_ISL_7970503	EPI_ISL_7970504	EPI_ISL_7970505
EPI_ISL_7970507	EPI_ISL_7970508	EPI_ISL_7970509	EPI_ISL_7970510	EPI_ISL_7970511
EPI_ISL_7970512	EPI_ISL_7970513	EPI_ISL_7970514	EPI_ISL_7970515	EPI_ISL_7970516
EPI_ISL_7970517	EPI_ISL_7970518	EPI_ISL_7970519	EPI_ISL_7970520	EPI_ISL_7970521
EPI_ISL_7970522	EPI_ISL_7970523	EPI_ISL_7970524	EPI_ISL_7970525	EPI_ISL_7970526
EPI_ISL_7970527	EPI_ISL_7970528	EPI_ISL_7970529	EPI_ISL_7970530	EPI_ISL_7970531
EPI_ISL_7970532	EPI_ISL_7970533	EPI_ISL_7970534	EPI_ISL_7970535	EPI_ISL_7970536
EPI_ISL_7970537	EPI_ISL_7970538	EPI_ISL_7970539	EPI_ISL_7970540	EPI_ISL_7970541
EPI_ISL_7970542	EPI_ISL_7970543	EPI_ISL_7970544	EPI_ISL_7970545	EPI_ISL_7970546
EPI_ISL_7970547	EPI_ISL_7970548	EPI_ISL_7970549	EPI_ISL_7970550	EPI_ISL_7970551
EPI_ISL_7970552	EPI_ISL_7970553	EPI_ISL_7970554	EPI_ISL_7970555	EPI_ISL_7970556
EPI_ISL_7970557	EPI_ISL_7970558	EPI_ISL_7970559	EPI_ISL_7970560	EPI_ISL_7970561
EPI_ISL_7970562	EPI_ISL_7970564	EPI_ISL_7970565	EPI_ISL_7970566	EPI_ISL_7970567
EPI_ISL_7970568	EPI_ISL_7970569	EPI_ISL_7970570	EPI_ISL_7970571	EPI_ISL_7970572
EPI_ISL_7970573	EPI_ISL_7970574	EPI_ISL_7970575	EPI_ISL_7970576	EPI_ISL_7970577
EPI_ISL_7970578	EPI_ISL_7970579	EPI_ISL_7970580	EPI_ISL_7970581	EPI_ISL_7970582
EPI_ISL_7970583	EPI_ISL_7970584	EPI_ISL_7970585	EPI_ISL_7970586	EPI_ISL_7970587
EPI_ISL_7970588	EPI_ISL_7970589	EPI_ISL_7970590	EPI_ISL_7970591	EPI_ISL_7970592
EPI_ISL_7970593	EPI_ISL_7970594	EPI_ISL_7970595	EPI_ISL_7970596	EPI_ISL_7970597
EPI_ISL_7970598	EPI_ISL_7970599	EPI_ISL_7970600	EPI_ISL_7970601	EPI_ISL_7970602
EPI_ISL_7970603	EPI_ISL_7970604	EPI_ISL_7970605	EPI_ISL_7970606	EPI_ISL_7970607
EPI_ISL_7970608	EPI_ISL_7970609	EPI_ISL_7970610	EPI_ISL_7970611	EPI_ISL_7970612
EPI_ISL_7970613	EPI_ISL_7970614	EPI_ISL_7970615	EPI_ISL_7970617	EPI_ISL_7970618
EPI_ISL_7970619	EPI_ISL_7970620	EPI_ISL_7970621	EPI_ISL_7970622	EPI_ISL_7970623
EPI_ISL_7970624	EPI_ISL_7970625	EPI_ISL_7970626	EPI_ISL_7970627	EPI_ISL_7970628
EPI_ISL_7970629	EPI_ISL_7970630	EPI_ISL_7970631	EPI_ISL_7970632	EPI_ISL_7970633
EPI_ISL_7970634	EPI_ISL_7970635	EPI_ISL_7970636	EPI_ISL_7970637	EPI_ISL_7970638
EPI_ISL_7970639	EPI_ISL_7970640	EPI_ISL_7970641	EPI_ISL_7970642	EPI_ISL_7970643
EPI_ISL_7970644	EPI_ISL_7970645	EPI_ISL_7970646	EPI_ISL_7970647	EPI_ISL_7970648
EPI_ISL_7970649	EPI_ISL_7970650	EPI_ISL_7970651	EPI_ISL_7970652	EPI_ISL_7970653
EPI_ISL_7970654	EPI_ISL_7970655	EPI_ISL_7970656	EPI_ISL_7970657	EPI_ISL_7970658
EPI_ISL_7970659	EPI_ISL_7970660	EPI_ISL_7970661	EPI_ISL_7970662	EPI_ISL_7970663
EPI_ISL_7970664	EPI_ISL_7970665	EPI_ISL_7970666	EPI_ISL_7970667	EPI_ISL_7970668
EPI_ISL_7970669	EPI_ISL_7970670	EPI_ISL_7970671	EPI_ISL_7970672	EPI_ISL_7970673
EPI_ISL_7970674	EPI_ISL_7970675	EPI_ISL_7970677	EPI_ISL_7970678	EPI_ISL_7970679
EPI_ISL_7970680	EPI_ISL_7970681	EPI_ISL_7970682	EPI_ISL_7970683	EPI_ISL_7970684

EPI_ISL_7970685	EPI_ISL_7970686	EPI_ISL_7970687	EPI_ISL_7970688	EPI_ISL_7970689
EPI_ISL_7970690	EPI_ISL_7970691	EPI_ISL_7970692	EPI_ISL_7970693	EPI_ISL_7970694
EPI_ISL_7970695	EPI_ISL_7970696	EPI_ISL_7970697	EPI_ISL_7970698	EPI_ISL_7970699
EPI_ISL_7970700	EPI_ISL_7970701	EPI_ISL_7970702	EPI_ISL_7970703	EPI_ISL_7970704
EPI_ISL_7970705	EPI_ISL_7970706	EPI_ISL_7970707	EPI_ISL_7970708	EPI_ISL_7970709
EPI_ISL_7970710	EPI_ISL_7970711	EPI_ISL_7970712	EPI_ISL_7970713	EPI_ISL_7970714
EPI_ISL_7970715	EPI_ISL_7970716	EPI_ISL_7970717	EPI_ISL_7970718	EPI_ISL_7970719
EPI_ISL_7970720	EPI_ISL_7970721	EPI_ISL_7970722	EPI_ISL_7970723	EPI_ISL_7970724
EPI_ISL_7970725	EPI_ISL_7970727	EPI_ISL_7970728	EPI_ISL_7970729	EPI_ISL_7970730
EPI_ISL_7970731	EPI_ISL_7970732	EPI_ISL_7970733	EPI_ISL_7970734	EPI_ISL_7970735
EPI_ISL_7970736	EPI_ISL_7970737	EPI_ISL_7970738	EPI_ISL_7970739	EPI_ISL_7970740
EPI_ISL_7970741	EPI_ISL_7970742	EPI_ISL_7970743	EPI_ISL_7970744	EPI_ISL_7970745
EPI_ISL_7970746	EPI_ISL_7970747	EPI_ISL_7970748	EPI_ISL_7970749	EPI_ISL_7970750
EPI_ISL_7970751	EPI_ISL_7970752	EPI_ISL_7970753	EPI_ISL_7970754	EPI_ISL_7970755
EPI_ISL_7970756	EPI_ISL_7970757	EPI_ISL_7970758	EPI_ISL_7970759	EPI_ISL_7970760
EPI_ISL_7970761	EPI_ISL_7970762	EPI_ISL_7970763	EPI_ISL_7970764	EPI_ISL_7970765
EPI_ISL_7970766	EPI_ISL_7970767	EPI_ISL_7970768	EPI_ISL_7970769	EPI_ISL_7970770
EPI_ISL_7970771	EPI_ISL_7970772	EPI_ISL_7970773	EPI_ISL_7970774	EPI_ISL_7970775
EPI_ISL_7970776	EPI_ISL_7970777	EPI_ISL_7970778	EPI_ISL_7970779	EPI_ISL_7970781
EPI_ISL_7970782	EPI_ISL_7970783	EPI_ISL_7970784	EPI_ISL_7970785	EPI_ISL_7970786
EPI_ISL_7970787	EPI_ISL_7970788	EPI_ISL_7970789	EPI_ISL_7970790	EPI_ISL_7970791
EPI_ISL_7970792	EPI_ISL_7970793	EPI_ISL_7970794	EPI_ISL_7970795	EPI_ISL_7970796
EPI_ISL_7970797	EPI_ISL_7970798	EPI_ISL_7970799	EPI_ISL_7970800	EPI_ISL_7970801
EPI_ISL_7970802	EPI_ISL_7970803	EPI_ISL_7970804	EPI_ISL_7970805	EPI_ISL_7970806
EPI_ISL_7970807	EPI_ISL_7970808	EPI_ISL_7970809	EPI_ISL_7970810	EPI_ISL_7970811
EPI_ISL_8120077	EPI_ISL_8120078	EPI_ISL_8120079	EPI_ISL_8120080	EPI_ISL_8120081
EPI_ISL_8120082	EPI_ISL_8120083	EPI_ISL_8120084	EPI_ISL_8120085	EPI_ISL_8120086
EPI_ISL_8120087	EPI_ISL_8120088	EPI_ISL_8120089	EPI_ISL_8120090	EPI_ISL_8120091
EPI_ISL_8120092	EPI_ISL_8120093	EPI_ISL_8120094	EPI_ISL_8120095	EPI_ISL_8120096
EPI_ISL_8120097	EPI_ISL_8120098	EPI_ISL_8120099	EPI_ISL_8120100	EPI_ISL_8120101
EPI_ISL_8120102	EPI_ISL_8120103	EPI_ISL_8120104	EPI_ISL_8120105	EPI_ISL_8120106
EPI_ISL_9609720	EPI_ISL_9609717	EPI_ISL_9609711	EPI_ISL_9609704	EPI_ISL_9609698
EPI_ISL_9609683	EPI_ISL_9609678	EPI_ISL_9609671	EPI_ISL_9609655	EPI_ISL_9609637
EPI_ISL_9609627	EPI_ISL_9609603	EPI_ISL_9609599	EPI_ISL_9609579	EPI_ISL_9609572
EPI_ISL_9609544	EPI_ISL_6279182	EPI_ISL_6278902	EPI_ISL_9594454	EPI_ISL_9594451
EPI_ISL_9594447	EPI_ISL_9594400	EPI_ISL_9594389	EPI_ISL_10068217	EPI_ISL_9829737
EPI_ISL_9829650	EPI_ISL_10437100	EPI_ISL_6183165	EPI_ISL_6183086	EPI_ISL_6182993
EPI_ISL_6182857	EPI_ISL_6182592	EPI_ISL_6182511	EPI_ISL_9307917	EPI_ISL_9307382
EPI_ISL_8414951	EPI_ISL_8414928	EPI_ISL_8414920	EPI_ISL_8414919	EPI_ISL_8391666
EPI_ISL_8391601	EPI_ISL_8391548	EPI_ISL_9206008	EPI_ISL_10437103	EPI_ISL_9152305
EPI_ISL_9130654	EPI_ISL_9130634	EPI_ISL_6131961	EPI_ISL_8316383	EPI_ISL_8316377
EPI_ISL_6915031	EPI_ISL_8922649	EPI_ISL_8922632	EPI_ISL_8922613	EPI_ISL_8922572
EPI_ISL_8922539	EPI_ISL_8267181	EPI_ISL_8267178	EPI_ISL_8267168	EPI_ISL_8267160
EPI_ISL_8267152	EPI_ISL_8267141	EPI_ISL_8264250	EPI_ISL_8264247	EPI_ISL_8264234
EPI_ISL_8264218	EPI_ISL_8264212	EPI_ISL_8264208	EPI_ISL_8264203	EPI_ISL_8264201
EPI_ISL_8264154	EPI_ISL_8264147	EPI_ISL_8264136	EPI_ISL_8252868	EPI_ISL_8252833
EPI_ISL_8252817	EPI_ISL_8252770	EPI_ISL_8252717	EPI_ISL_8252710	EPI_ISL_8764284
EPI_ISL_8764270	EPI_ISL_8764267	EPI_ISL_8764258	EPI_ISL_8764252	EPI_ISL_8764251
EPI_ISL_8764209	EPI_ISL_8764171	EPI_ISL_8764150	EPI_ISL_8764149	EPI_ISL_10435714
EPI_ISL_5942497	EPI_ISL_5937342	EPI_ISL_8542174	EPI_ISL_8542124	EPI_ISL_8542122
EPI_ISL_8542109	EPI_ISL_8542104	EPI_ISL_8542103	EPI_ISL_8542076	EPI_ISL_8542057
EPI_ISL_8542046	EPI_ISL_8542043	EPI_ISL_8542015	EPI_ISL_8542011	EPI_ISL_8057358
EPI_ISL_8057329	EPI_ISL_8057327	EPI_ISL_8057316	EPI_ISL_8057301	EPI_ISL_8057279
EPI_ISL_8057258	EPI_ISL_8057249	EPI_ISL_8057224	EPI_ISL_8057218	EPI_ISL_8057216
EPI_ISL_8057203	EPI_ISL_8057195	EPI_ISL_10068226	EPI_ISL_8038796	EPI_ISL_8038690

EPI_ISL_8038686	EPI_ISL_8038657	EPI_ISL_8035651	EPI_ISL_7964892	EPI_ISL_7964881
EPI_ISL_7964864	EPI_ISL_7964850	EPI_ISL_7964826	EPI_ISL_7964800	EPI_ISL_7964704
EPI_ISL_7964700	EPI_ISL_7964698	EPI_ISL_7964646	EPI_ISL_7964632	EPI_ISL_7964627
EPI_ISL_7964624	EPI_ISL_7964620	EPI_ISL_7964618	EPI_ISL_7939540	EPI_ISL_7939528
EPI_ISL_7939515	EPI_ISL_10437132	EPI_ISL_7849452	EPI_ISL_7849450	EPI_ISL_7849428
EPI_ISL_7849402	EPI_ISL_7849401	EPI_ISL_7849398	EPI_ISL_10068234	EPI_ISL_7668455
EPI_ISL_7657295	EPI_ISL_7657273	EPI_ISL_7657266	EPI_ISL_7657225	EPI_ISL_7657194
EPI_ISL_7657059	EPI_ISL_7656693	EPI_ISL_7656670	EPI_ISL_7656532	EPI_ISL_7656399
EPI_ISL_7656334	EPI_ISL_7656183	EPI_ISL_7568767	EPI_ISL_7591976	EPI_ISL_7591851
EPI_ISL_7591734	EPI_ISL_7591717	EPI_ISL_7591703	EPI_ISL_7591654	EPI_ISL_7591596
EPI_ISL_7588804	EPI_ISL_7588492	EPI_ISL_7588335	EPI_ISL_7588309	EPI_ISL_7588299
EPI_ISL_7573343	EPI_ISL_7568948	EPI_ISL_7568920	EPI_ISL_7568916	EPI_ISL_7568913
EPI_ISL_7568911	EPI_ISL_7568905	EPI_ISL_7568895	EPI_ISL_7568890	EPI_ISL_7568888
EPI_ISL_7568860	EPI_ISL_7568859	EPI_ISL_7568857	EPI_ISL_7568855	EPI_ISL_7568843
EPI_ISL_7568838	EPI_ISL_7568805	EPI_ISL_7568790	EPI_ISL_7568785	EPI_ISL_7568685
EPI_ISL_7568674	EPI_ISL_7568670	EPI_ISL_7568661	EPI_ISL_7568638	EPI_ISL_7507097
EPI_ISL_7507084	EPI_ISL_7507060	EPI_ISL_7404640	EPI_ISL_7404638	EPI_ISL_7404627
EPI_ISL_7404609	EPI_ISL_7404594	EPI_ISL_7404575	EPI_ISL_7404572	EPI_ISL_7380586
EPI_ISL_8391628	EPI_ISL_8391629	EPI_ISL_8391648	EPI_ISL_8391649	EPI_ISL_8391622
EPI_ISL_8391676	EPI_ISL_8391551	EPI_ISL_8391549	EPI_ISL_8391583	EPI_ISL_7212174
EPI_ISL_7212140	EPI_ISL_7212108	EPI_ISL_7212040	EPI_ISL_7212023	EPI_ISL_7211973
EPI_ISL_7211908	EPI_ISL_7167681	EPI_ISL_7167656	EPI_ISL_7167626	EPI_ISL_7167581
EPI_ISL_7167577	EPI_ISL_7167542	EPI_ISL_7167499	EPI_ISL_7167444	EPI_ISL_7167417
EPI_ISL_7167400	EPI_ISL_7167392	EPI_ISL_9829742	EPI_ISL_7122230	EPI_ISL_7121865
EPI_ISL_7121774	EPI_ISL_7062457	EPI_ISL_7062372	EPI_ISL_7061968	EPI_ISL_7060425
EPI_ISL_7060257	EPI_ISL_6971426	EPI_ISL_6971400	EPI_ISL_6971185	EPI_ISL_6971161
EPI_ISL_6971097	EPI_ISL_6915042	EPI_ISL_6915016	EPI_ISL_6915013	EPI_ISL_6826226
EPI_ISL_6826108	EPI_ISL_6826077	EPI_ISL_6826050	EPI_ISL_6826043	EPI_ISL_6826012
EPI_ISL_6826007	EPI_ISL_6825920	EPI_ISL_8414911	EPI_ISL_6781233	EPI_ISL_6781098
EPI_ISL_6781092	EPI_ISL_6781078	EPI_ISL_6780486	EPI_ISL_6780480	EPI_ISL_6780458
EPI_ISL_6780380	EPI_ISL_6780365	EPI_ISL_6780281	EPI_ISL_6780275	EPI_ISL_6780179
EPI_ISL_6630585	EPI_ISL_6630226	EPI_ISL_6630114	EPI_ISL_6604031	EPI_ISL_6603969
EPI_ISL_6590942	EPI_ISL_6590934	EPI_ISL_6590881	EPI_ISL_7051939	EPI_ISL_7060044
EPI_ISL_7051272	EPI_ISL_7060620	EPI_ISL_7051746	EPI_ISL_9306951	EPI_ISL_6437349
EPI_ISL_6437323	EPI_ISL_6437223	EPI_ISL_6437028	EPI_ISL_6428439	EPI_ISL_6428279
EPI_ISL_6427930	EPI_ISL_6427630	EPI_ISL_7060560	EPI_ISL_7060574	EPI_ISL_7060595
EPI_ISL_7062742	EPI_ISL_7061087	EPI_ISL_9829755	EPI_ISL_7060235	EPI_ISL_5570329
EPI_ISL_5570130	EPI_ISL_5569907	EPI_ISL_5512076	EPI_ISL_5512001	EPI_ISL_6247205
EPI_ISL_6247285	EPI_ISL_9306964	EPI_ISL_6273503	EPI_ISL_6278975	EPI_ISL_5260608
EPI_ISL_5248335	EPI_ISL_10068266	EPI_ISL_10068268	EPI_ISL_5114640	EPI_ISL_5114628
EPI_ISL_5114579	EPI_ISL_5114437	EPI_ISL_5114377	EPI_ISL_5114334	EPI_ISL_5114256
EPI_ISL_10069034	EPI_ISL_10069032	EPI_ISL_10069029	EPI_ISL_10069023	EPI_ISL_10069019
EPI_ISL_10069011	EPI_ISL_10069010	EPI_ISL_10069002	EPI_ISL_10068995	EPI_ISL_10068985
EPI_ISL_10068980	EPI_ISL_10068977	EPI_ISL_10068972	EPI_ISL_10068962	EPI_ISL_10068958
EPI_ISL_10068957	EPI_ISL_10068956	EPI_ISL_10068955	EPI_ISL_10068296	EPI_ISL_10068292
EPI_ISL_10068290	EPI_ISL_10068288	EPI_ISL_10068284	EPI_ISL_10068280	EPI_ISL_10068276
EPI_ISL_10068243	EPI_ISL_10068223	EPI_ISL_10068214	EPI_ISL_8922673	EPI_ISL_10068273
EPI_ISL_7122082	EPI_ISL_7121656	EPI_ISL_7121632	EPI_ISL_9829728	EPI_ISL_5416597
EPI_ISL_5416622	EPI_ISL_5416611	EPI_ISL_7122245	EPI_ISL_7122225	EPI_ISL_7121839
EPI_ISL_7121846	EPI_ISL_4741775	EPI_ISL_9829658	EPI_ISL_5433919	EPI_ISL_5433966
EPI_ISL_5433936	EPI_ISL_5433853	EPI_ISL_5470202	EPI_ISL_5470236	EPI_ISL_5470141
EPI_ISL_5512086	EPI_ISL_5512023	EPI_ISL_6428130	EPI_ISL_6437191	EPI_ISL_6437218
EPI_ISL_8391643	EPI_ISL_5611808	EPI_ISL_5611865	EPI_ISL_5611303	EPI_ISL_5612682
EPI_ISL_7167686	EPI_ISL_7167700	EPI_ISL_7167703	EPI_ISL_7167485	EPI_ISL_7167477
EPI_ISL_7167478	EPI_ISL_7167471	EPI_ISL_7167442	EPI_ISL_7167530	EPI_ISL_7167500

EPI_ISL_7167579	EPI_ISL_7167673	EPI_ISL_7167627	EPI_ISL_7167634	EPI_ISL_7166882
EPI_ISL_7121644	EPI_ISL_6689907	EPI_ISL_6689912	EPI_ISL_8252744	EPI_ISL_7964744
EPI_ISL_7212065	EPI_ISL_7211917	EPI_ISL_7211957	EPI_ISL_7211886	EPI_ISL_7211968
EPI_ISL_7211988	EPI_ISL_7212142	EPI_ISL_7212165	EPI_ISL_7212163	EPI_ISL_7212117
EPI_ISL_7166893	EPI_ISL_6590913	EPI_ISL_7167705	EPI_ISL_6590814	EPI_ISL_6603996
EPI_ISL_6604079	EPI_ISL_6604015	EPI_ISL_7404604	EPI_ISL_8391646	EPI_ISL_7404600
EPI_ISL_7404615	EPI_ISL_7404624	EPI_ISL_7404642	EPI_ISL_8764211	EPI_ISL_7568682
EPI_ISL_7568731	EPI_ISL_7568730	EPI_ISL_7568765	EPI_ISL_7568762	EPI_ISL_7568761
EPI_ISL_7568828	EPI_ISL_7568868	EPI_ISL_7568910	EPI_ISL_7573282	EPI_ISL_7573273
EPI_ISL_7591698	EPI_ISL_7591789	EPI_ISL_7588288	EPI_ISL_7591872	EPI_ISL_7588304
EPI_ISL_7588365	EPI_ISL_7648815	EPI_ISL_7656530	EPI_ISL_7656748	EPI_ISL_7657216
EPI_ISL_7657131	EPI_ISL_7657187	EPI_ISL_7657252	EPI_ISL_7657294	EPI_ISL_7657272
EPI_ISL_7656805	EPI_ISL_7656106	EPI_ISL_7656924	EPI_ISL_7656325	EPI_ISL_7656417
EPI_ISL_7656465	EPI_ISL_7655951	EPI_ISL_7655928	EPI_ISL_8836719	EPI_ISL_8836684
EPI_ISL_8057207	EPI_ISL_8264215	EPI_ISL_7964912	EPI_ISL_7507061	EPI_ISL_7507103
EPI_ISL_8252928	EPI_ISL_5942622	EPI_ISL_5942574	EPI_ISL_5612941	EPI_ISL_7849345
EPI_ISL_7939518	EPI_ISL_8038799	EPI_ISL_8038766	EPI_ISL_8038660	EPI_ISL_8038757
EPI_ISL_8038706	EPI_ISL_8391657	EPI_ISL_8267158	EPI_ISL_8252727	EPI_ISL_5937366
EPI_ISL_9794783	EPI_ISL_5942519	EPI_ISL_8252701	EPI_ISL_6131735	EPI_ISL_6247058
EPI_ISL_6247103	EPI_ISL_7964765	EPI_ISL_7964684	EPI_ISL_8252684	EPI_ISL_7568952
EPI_ISL_7591552	EPI_ISL_7656362	EPI_ISL_8764184	EPI_ISL_7656533	EPI_ISL_7657213
EPI_ISL_7657190	EPI_ISL_7657150	EPI_ISL_7656802	EPI_ISL_6437299	EPI_ISL_8267163
EPI_ISL_8764285	EPI_ISL_6427745	EPI_ISL_6427839	EPI_ISL_8542123	EPI_ISL_7211828
EPI_ISL_7568688	EPI_ISL_7657015	EPI_ISL_7657254	EPI_ISL_7656259	EPI_ISL_8764186
EPI_ISL_7964686	EPI_ISL_8542186	EPI_ISL_8252802	EPI_ISL_7591783	EPI_ISL_6034079
EPI_ISL_6034055	EPI_ISL_6034113	EPI_ISL_6034136	EPI_ISL_6034080	EPI_ISL_8764175
EPI_ISL_6603997	EPI_ISL_8391671	EPI_ISL_8391651	EPI_ISL_8391565	EPI_ISL_8391674
EPI_ISL_7849397	EPI_ISL_8264251	EPI_ISL_6034284	EPI_ISL_7404613	EPI_ISL_8391558
EPI_ISL_9682668	EPI_ISL_8922559	EPI_ISL_5937389	EPI_ISL_8542164	EPI_ISL_7657249
EPI_ISL_8252628	EPI_ISL_8038792	EPI_ISL_8267190	EPI_ISL_6132920	EPI_ISL_6131936
EPI_ISL_6132803	EPI_ISL_6132862	EPI_ISL_9794690	EPI_ISL_9794682	EPI_ISL_8391691
EPI_ISL_7964592	EPI_ISL_8264192	EPI_ISL_7964740	EPI_ISL_8542165	EPI_ISL_8252765
EPI_ISL_8252840	EPI_ISL_9682492	EPI_ISL_7849365	EPI_ISL_6182922	EPI_ISL_6278762
EPI_ISL_6278940	EPI_ISL_8922694	EPI_ISL_6971165	EPI_ISL_6971444	EPI_ISL_8264195
EPI_ISL_8252742	EPI_ISL_6826268	EPI_ISL_8267166	EPI_ISL_8252766	EPI_ISL_7964796
EPI_ISL_8035653	EPI_ISL_8267179	EPI_ISL_9682430	EPI_ISL_4741701	EPI_ISL_8764197
EPI_ISL_8764198	EPI_ISL_9829777	EPI_ISL_9609652	EPI_ISL_9308251	EPI_ISL_9307919
EPI_ISL_9793272	EPI_ISL_8264236	EPI_ISL_8542167	EPI_ISL_7964898	EPI_ISL_8038698
EPI_ISL_10068994	EPI_ISL_10113863	EPI_ISL_10435680	EPI_ISL_9307411	EPI_ISL_10068258
EPI_ISL_9829785	EPI_ISL_10436441	EPI_ISL_9793244	EPI_ISL_9306884	EPI_ISL_8267172
EPI_ISL_7964872	EPI_ISL_8252673	EPI_ISL_7380611	EPI_ISL_7380810	EPI_ISL_7404628
EPI_ISL_9594432	EPI_ISL_9609598	EPI_ISL_7964876	EPI_ISL_8542048	EPI_ISL_8038755
EPI_ISL_8057363	EPI_ISL_8252677	EPI_ISL_6630047	EPI_ISL_6781236	EPI_ISL_7656289
EPI_ISL_6780419	EPI_ISL_6780439	EPI_ISL_7656644	EPI_ISL_6780329	EPI_ISL_6780263
EPI_ISL_6781145	EPI_ISL_6780665	EPI_ISL_6826002	EPI_ISL_6826089	EPI_ISL_6826165
EPI_ISL_6826139	EPI_ISL_6826213	EPI_ISL_6826290	EPI_ISL_6826250	EPI_ISL_6826251
EPI_ISL_6825939	EPI_ISL_6825938	EPI_ISL_6835524	EPI_ISL_7964878	EPI_ISL_9682526
EPI_ISL_9682527	EPI_ISL_9682565	EPI_ISL_9682592	EPI_ISL_9682424	EPI_ISL_9682418
EPI_ISL_9682467	EPI_ISL_9682346	EPI_ISL_9682347	EPI_ISL_9682601	EPI_ISL_9682652
EPI_ISL_9682658	EPI_ISL_6866252	EPI_ISL_9790814	EPI_ISL_9790803	EPI_ISL_8252831
EPI_ISL_8252700	EPI_ISL_8252743	EPI_ISL_8252791	EPI_ISL_8252826	EPI_ISL_8252822
EPI_ISL_9794798	EPI_ISL_8252904	EPI_ISL_9794772	EPI_ISL_9794744	EPI_ISL_8252877
EPI_ISL_9793332	EPI_ISL_9793323	EPI_ISL_8252674	EPI_ISL_8252641	EPI_ISL_8252646
EPI_ISL_8057280	EPI_ISL_9794969	EPI_ISL_9794916	EPI_ISL_9794841	EPI_ISL_9794807
EPI_ISL_8391633	EPI_ISL_8038733	EPI_ISL_8391634	EPI_ISL_6034092	EPI_ISL_8038780

EPI_ISL_9682380	EPI_ISL_8542181	EPI_ISL_7849399	EPI_ISL_6246791	EPI_ISL_6182885
EPI_ISL_7656269	EPI_ISL_7656636	EPI_ISL_5937357	EPI_ISL_7657127	EPI_ISL_7657154
EPI_ISL_7657215	EPI_ISL_6825936	EPI_ISL_6780170	EPI_ISL_6780398	EPI_ISL_6780400
EPI_ISL_6780410	EPI_ISL_6780423	EPI_ISL_6780475	EPI_ISL_6780504	EPI_ISL_6780509
EPI_ISL_6825924	EPI_ISL_8267149	EPI_ISL_8542050	EPI_ISL_7167534	EPI_ISL_8542153
EPI_ISL_8252713	EPI_ISL_8038682	EPI_ISL_7565191	EPI_ISL_6915041	EPI_ISL_7380583
EPI_ISL_6131935	EPI_ISL_10116124	EPI_ISL_10437152	EPI_ISL_10437122	EPI_ISL_10437114
EPI_ISL_10437099	EPI_ISL_10437098	EPI_ISL_10437092	EPI_ISL_10437088	EPI_ISL_10437084
EPI_ISL_10437077	EPI_ISL_10437074	EPI_ISL_10437071	EPI_ISL_10436473	EPI_ISL_10436463
EPI_ISL_10436445	EPI_ISL_10436439	EPI_ISL_10436405	EPI_ISL_10436396	EPI_ISL_10436395
EPI_ISL_10436392	EPI_ISL_10435748	EPI_ISL_10435744	EPI_ISL_10435734	EPI_ISL_10435723
EPI_ISL_10435708	EPI_ISL_10435704	EPI_ISL_10435697	EPI_ISL_7964669	EPI_ISL_8038772
EPI_ISL_8542172	EPI_ISL_4986097	EPI_ISL_5569883	EPI_ISL_10771048	EPI_ISL_10771045
EPI_ISL_10771039	EPI_ISL_10771021	EPI_ISL_10771015	EPI_ISL_10771010	EPI_ISL_10771007
EPI_ISL_10770999	EPI_ISL_10770991	EPI_ISL_10770979	EPI_ISL_10770975	EPI_ISL_10770968
EPI_ISL_10770965	EPI_ISL_10770961	EPI_ISL_10770944	EPI_ISL_10770936	EPI_ISL_10770917
EPI_ISL_10770908	EPI_ISL_10770902	EPI_ISL_10770893	EPI_ISL_10770884	EPI_ISL_10770879
EPI_ISL_10770869	EPI_ISL_10770867	EPI_ISL_10770863	EPI_ISL_10770860	EPI_ISL_10770855
EPI_ISL_10770817	EPI_ISL_10770815	EPI_ISL_10770804	EPI_ISL_10770796	EPI_ISL_10770783
EPI_ISL_10770777	EPI_ISL_10770775	EPI_ISL_10770768	EPI_ISL_10770765	EPI_ISL_10770761
EPI_ISL_10770755	EPI_ISL_10770749	EPI_ISL_10770522	EPI_ISL_10770477	EPI_ISL_10770474
EPI_ISL_10770457	EPI_ISL_10770450	EPI_ISL_10770447	EPI_ISL_10770439	EPI_ISL_10770434
EPI_ISL_10770426	EPI_ISL_10770414	EPI_ISL_10770391	EPI_ISL_10770378	EPI_ISL_10770370
EPI_ISL_10770367	EPI_ISL_10770340	EPI_ISL_10770339	EPI_ISL_10770331	EPI_ISL_10770326
EPI_ISL_10770321	EPI_ISL_10770315	EPI_ISL_10770309	EPI_ISL_10770305	EPI_ISL_10770295
EPI_ISL_10770294	EPI_ISL_10770275	EPI_ISL_10765908	EPI_ISL_10765897	EPI_ISL_10765880
EPI_ISL_10765866	EPI_ISL_10765850	EPI_ISL_10765842	EPI_ISL_10765833	EPI_ISL_10765809
EPI_ISL_10765800	EPI_ISL_10765787	EPI_ISL_10765762	EPI_ISL_10765724	EPI_ISL_10765722
EPI_ISL_10765689	EPI_ISL_10765669	EPI_ISL_10765615	EPI_ISL_10765614	EPI_ISL_10765604
EPI_ISL_10765583	EPI_ISL_10765569	EPI_ISL_10765565	EPI_ISL_10765558	EPI_ISL_10765557
EPI_ISL_10765547	EPI_ISL_10765536	EPI_ISL_10764910	EPI_ISL_10764906	EPI_ISL_10764905
EPI_ISL_10764889	EPI_ISL_10764886	EPI_ISL_10764884	EPI_ISL_10764879	EPI_ISL_10764870
EPI_ISL_10764867	EPI_ISL_10764864	EPI_ISL_10764840	EPI_ISL_10764834	EPI_ISL_10764826
EPI_ISL_10764820	EPI_ISL_10764814	EPI_ISL_10764811	EPI_ISL_10764805	EPI_ISL_10764787
EPI_ISL_10764783	EPI_ISL_10764779	EPI_ISL_10764776	EPI_ISL_10764767	EPI_ISL_10764757
EPI_ISL_10764741	EPI_ISL_10764737	EPI_ISL_10764731	EPI_ISL_10764137	EPI_ISL_10764129
EPI_ISL_10764122	EPI_ISL_10764112	EPI_ISL_10764107	EPI_ISL_10764105	EPI_ISL_10764097
EPI_ISL_10764082	EPI_ISL_10764072	EPI_ISL_10764042	EPI_ISL_10764038	EPI_ISL_10764031
EPI_ISL_10764029	EPI_ISL_10764020	EPI_ISL_10764016	EPI_ISL_10764009	EPI_ISL_10764006
EPI_ISL_10763975	EPI_ISL_10763907	EPI_ISL_10763897	EPI_ISL_10763889	EPI_ISL_10763887
EPI_ISL_10763884	EPI_ISL_10763883	EPI_ISL_10763882	EPI_ISL_10763871	EPI_ISL_10763858
EPI_ISL_10763856	EPI_ISL_10763855	EPI_ISL_10763851	EPI_ISL_10763837	EPI_ISL_10702454
EPI_ISL_10702444	EPI_ISL_10702435	EPI_ISL_10702413	EPI_ISL_10702411	EPI_ISL_10702405
EPI_ISL_10702384	EPI_ISL_10702368	EPI_ISL_10702313	EPI_ISL_10702312	EPI_ISL_10702302
EPI_ISL_10702299	EPI_ISL_10702294	EPI_ISL_10702255	EPI_ISL_10702226	EPI_ISL_10702206
EPI_ISL_10702194	EPI_ISL_10701975	EPI_ISL_10701970	EPI_ISL_10701952	EPI_ISL_10701945
EPI_ISL_10701738	EPI_ISL_10701718	EPI_ISL_10701717	EPI_ISL_10701705	EPI_ISL_10701703
EPI_ISL_10701702	EPI_ISL_10701700	EPI_ISL_10701689	EPI_ISL_10701674	EPI_ISL_10701668
EPI_ISL_10701663	EPI_ISL_10701637	EPI_ISL_10701634	EPI_ISL_10701633	EPI_ISL_10701623
EPI_ISL_10701619	EPI_ISL_10701598	EPI_ISL_10701562	EPI_ISL_10701551	EPI_ISL_10701527
EPI_ISL_10701526	EPI_ISL_10701505	EPI_ISL_10701490	EPI_ISL_10701472	EPI_ISL_10701456
EPI_ISL_10701429	EPI_ISL_10700352	EPI_ISL_10700351	EPI_ISL_10700344	EPI_ISL_10700308
EPI_ISL_10700306	EPI_ISL_10700295	EPI_ISL_10700293	EPI_ISL_10700277	EPI_ISL_10700274
EPI_ISL_10700267	EPI_ISL_10700262	EPI_ISL_10700251	EPI_ISL_10700248	EPI_ISL_10700242
EPI_ISL_10700233	EPI_ISL_10700230	EPI_ISL_10700204	EPI_ISL_10700137	EPI_ISL_10700136

EPI_ISL_10700134	EPI_ISL_10700126	EPI_ISL_10699748	EPI_ISL_10699703	EPI_ISL_10699700
EPI_ISL_10699699	EPI_ISL_10699698	EPI_ISL_10699677	EPI_ISL_10699665	EPI_ISL_10699662
EPI_ISL_10699659	EPI_ISL_10699589	EPI_ISL_10699543	EPI_ISL_10699542	EPI_ISL_10699459
EPI_ISL_10699456	EPI_ISL_10699451	EPI_ISL_10699446	EPI_ISL_10699418	EPI_ISL_10699414
EPI_ISL_10689241	EPI_ISL_10689238	EPI_ISL_10689236	EPI_ISL_10689227	EPI_ISL_10689187
EPI_ISL_10689183	EPI_ISL_10689181	EPI_ISL_10689169	EPI_ISL_10689167	EPI_ISL_10689107
EPI_ISL_10689095	EPI_ISL_10689065	EPI_ISL_10689061	EPI_ISL_10689059	EPI_ISL_10689031
EPI_ISL_10689028	EPI_ISL_10689020	EPI_ISL_10689010	EPI_ISL_10688998	EPI_ISL_10688994
EPI_ISL_10688993	EPI_ISL_10688982	EPI_ISL_10123316	EPI_ISL_10113861	EPI_ISL_10113850
EPI_ISL_10113845	EPI_ISL_10113843	EPI_ISL_10113839	EPI_ISL_10113838	EPI_ISL_10103800
EPI_ISL_10103799	EPI_ISL_10103797	EPI_ISL_10103796	EPI_ISL_9829794	EPI_ISL_9829789
EPI_ISL_9829784	EPI_ISL_9829780	EPI_ISL_9829775	EPI_ISL_9829766	EPI_ISL_9829758
EPI_ISL_9829738	EPI_ISL_9829696	EPI_ISL_9829670	EPI_ISL_9829654	EPI_ISL_9794967
EPI_ISL_9794957	EPI_ISL_9794919	EPI_ISL_9794838	EPI_ISL_9793329	EPI_ISL_9793158
EPI_ISL_9790804	EPI_ISL_9682659	EPI_ISL_9682615	EPI_ISL_9682589	EPI_ISL_9682559
EPI_ISL_9682497	EPI_ISL_9682473	EPI_ISL_9682404	EPI_ISL_9682397	EPI_ISL_10771049
EPI_ISL_10771052	EPI_ISL_10771481	EPI_ISL_10771500	EPI_ISL_10771525	EPI_ISL_10771542
EPI_ISL_10771557	EPI_ISL_10771558	EPI_ISL_10771567	EPI_ISL_10773372	EPI_ISL_10773378
EPI_ISL_10773379	EPI_ISL_10773389	EPI_ISL_10773399	EPI_ISL_10773427	EPI_ISL_10773431
EPI_ISL_10773433	EPI_ISL_10773435	EPI_ISL_10773439	EPI_ISL_10773444	EPI_ISL_10773483
EPI_ISL_10773490	EPI_ISL_10773494	EPI_ISL_10773501	EPI_ISL_10773509	EPI_ISL_10774478
EPI_ISL_10774521	EPI_ISL_10774561	EPI_ISL_10774582	EPI_ISL_10774625	EPI_ISL_10774645
EPI_ISL_10774647	EPI_ISL_10774650	EPI_ISL_10774662	EPI_ISL_10774666	EPI_ISL_10774671
EPI_ISL_10774675	EPI_ISL_10774680	EPI_ISL_10774683	EPI_ISL_10774703	EPI_ISL_10774705
EPI_ISL_10774710	EPI_ISL_10774713	EPI_ISL_10774715	EPI_ISL_10774741	EPI_ISL_10774745
EPI_ISL_10774761	EPI_ISL_10774774	EPI_ISL_10774781	EPI_ISL_10774786	EPI_ISL_10774788
EPI_ISL_10774791	EPI_ISL_10774799	EPI_ISL_10774800	EPI_ISL_10774802	EPI_ISL_10774814
EPI_ISL_10774815	EPI_ISL_10774817	EPI_ISL_10774881	EPI_ISL_10774882	EPI_ISL_10774886
EPI_ISL_10774900	EPI_ISL_10774905	EPI_ISL_10774916	EPI_ISL_10774925	EPI_ISL_10774939
EPI_ISL_10774941	EPI_ISL_10774971	EPI_ISL_10774973	EPI_ISL_10774996	EPI_ISL_10775000
EPI_ISL_10775003	EPI_ISL_10775006	EPI_ISL_10775011	EPI_ISL_10775012	EPI_ISL_10775013
EPI_ISL_10775022	EPI_ISL_10775024	EPI_ISL_10775025	EPI_ISL_10775027	EPI_ISL_10775033
EPI_ISL_10775037	EPI_ISL_10775042	EPI_ISL_10775046	EPI_ISL_10775056	EPI_ISL_10775064
EPI_ISL_10775070	EPI_ISL_10775075	EPI_ISL_10775079	EPI_ISL_10775082	EPI_ISL_10775090
EPI_ISL_10775095	EPI_ISL_10775100	EPI_ISL_10775104	EPI_ISL_10775110	EPI_ISL_10775116
EPI_ISL_10775123	EPI_ISL_10775127	EPI_ISL_10775130	EPI_ISL_10775139	EPI_ISL_10775162
EPI_ISL_10775165	EPI_ISL_10775181	EPI_ISL_10775182	EPI_ISL_10775184	EPI_ISL_10775186
EPI_ISL_10775192	EPI_ISL_10775194	EPI_ISL_10775430	EPI_ISL_10775442	EPI_ISL_10775443
EPI_ISL_10775445	EPI_ISL_10775447	EPI_ISL_10775449	EPI_ISL_10775453	EPI_ISL_10775470
EPI_ISL_10775482	EPI_ISL_10775497	EPI_ISL_10775504	EPI_ISL_10775512	EPI_ISL_10775516
EPI_ISL_10775519	EPI_ISL_10775531	EPI_ISL_10775532	EPI_ISL_10775540	EPI_ISL_10775547
EPI_ISL_10775548	EPI_ISL_10775549	EPI_ISL_10775553	EPI_ISL_10775560	EPI_ISL_10775565
EPI_ISL_10775586	EPI_ISL_10775588	EPI_ISL_10775603	EPI_ISL_10775606	EPI_ISL_10775610
EPI_ISL_10775611	EPI_ISL_10775619	EPI_ISL_10775623	EPI_ISL_10775634	EPI_ISL_10775639
EPI_ISL_10775645	EPI_ISL_10775668	EPI_ISL_10775685	EPI_ISL_10775691	EPI_ISL_10775698
EPI_ISL_10775704	EPI_ISL_10775722	EPI_ISL_10775731	EPI_ISL_10775737	EPI_ISL_10775748
EPI_ISL_10775749	EPI_ISL_10775752	EPI_ISL_10775759	EPI_ISL_10775763	EPI_ISL_10775776
EPI_ISL_10775782	EPI_ISL_10943527	EPI_ISL_10943601	EPI_ISL_10944502	EPI_ISL_10969168
EPI_ISL_10969170	EPI_ISL_10969172	EPI_ISL_10969186	EPI_ISL_10969194	EPI_ISL_10969195
EPI_ISL_10969202	EPI_ISL_10969203	EPI_ISL_10969209	EPI_ISL_10969214	EPI_ISL_10969216
EPI_ISL_10969222	EPI_ISL_10969270	EPI_ISL_10969282	EPI_ISL_10969299	EPI_ISL_10969302
EPI_ISL_10969323	EPI_ISL_10969335	EPI_ISL_10969372	EPI_ISL_10969375	EPI_ISL_10969377
EPI_ISL_10969411	EPI_ISL_10984208	EPI_ISL_10984212	EPI_ISL_10984223	EPI_ISL_10984247
EPI_ISL_10984266	EPI_ISL_10984274	EPI_ISL_10984277	EPI_ISL_10984279	EPI_ISL_10984283
EPI_ISL_10984298	EPI_ISL_10984300	EPI_ISL_10984302	EPI_ISL_10984309	EPI_ISL_10984320

EPI_ISL_10984327	EPI_ISL_10984337	EPI_ISL_10984346	EPI_ISL_10984364	EPI_ISL_10984386
EPI_ISL_10984401	EPI_ISL_10984408	EPI_ISL_10984421	EPI_ISL_10984427	EPI_ISL_10984447
EPI_ISL_10984481	EPI_ISL_10984484	EPI_ISL_10984490	EPI_ISL_10984503	EPI_ISL_10984527
EPI_ISL_10984558	EPI_ISL_10984564	EPI_ISL_10984573	EPI_ISL_10984574	EPI_ISL_10984576
EPI_ISL_10984579	EPI_ISL_10984592	EPI_ISL_10984598	EPI_ISL_10984621	EPI_ISL_10984628
EPI_ISL_10984634	EPI_ISL_10984642	EPI_ISL_10984653	EPI_ISL_10984700	EPI_ISL_10984717
EPI_ISL_10984725	EPI_ISL_11111211	EPI_ISL_11111226	EPI_ISL_11111232	EPI_ISL_11111252
EPI_ISL_11111276	EPI_ISL_11111286	EPI_ISL_11111289	EPI_ISL_11114239	EPI_ISL_11114265
EPI_ISL_11114273	EPI_ISL_11114274	EPI_ISL_11114297	EPI_ISL_11260284	EPI_ISL_11260288
EPI_ISL_11260290	EPI_ISL_11260315	EPI_ISL_11260349	EPI_ISL_11260363	EPI_ISL_11260373
EPI_ISL_11260382	EPI_ISL_11260390	EPI_ISL_11260400	EPI_ISL_11260406	EPI_ISL_11260410
EPI_ISL_11260419	EPI_ISL_11260438	EPI_ISL_11260441	EPI_ISL_11260449	EPI_ISL_11260451
EPI_ISL_11260457	EPI_ISL_11262133	EPI_ISL_11262135	EPI_ISL_11262148	EPI_ISL_11262151
EPI_ISL_11262161	EPI_ISL_11262172	EPI_ISL_11262175	EPI_ISL_11262197	EPI_ISL_11262212
EPI_ISL_11262216	EPI_ISL_11262228	EPI_ISL_11262234	EPI_ISL_11262238	EPI_ISL_11262240
EPI_ISL_11262248	EPI_ISL_11262251	EPI_ISL_11262263	EPI_ISL_11262267	EPI_ISL_11262274
EPI_ISL_11262307	EPI_ISL_11262312	EPI_ISL_11262322	EPI_ISL_11262327	EPI_ISL_11262347
EPI_ISL_11262352	EPI_ISL_11262365	EPI_ISL_11262384	EPI_ISL_11262399	EPI_ISL_11262413
EPI_ISL_11262430	EPI_ISL_11262432	EPI_ISL_11262457	EPI_ISL_11262470	EPI_ISL_11262471
EPI_ISL_11262502	EPI_ISL_11262508	EPI_ISL_11262522	EPI_ISL_11262524	EPI_ISL_11262526
EPI_ISL_11262543	EPI_ISL_11262544	EPI_ISL_11262556	EPI_ISL_11262557	EPI_ISL_11262567
EPI_ISL_11309986	EPI_ISL_11310020	EPI_ISL_11310038	EPI_ISL_11310040	EPI_ISL_11310056
EPI_ISL_11310086	EPI_ISL_11310088	EPI_ISL_11310110	EPI_ISL_11310144	EPI_ISL_11310159
EPI_ISL_11310162	EPI_ISL_11310173	EPI_ISL_11310177	EPI_ISL_11310182	EPI_ISL_11310187
EPI_ISL_11310220	EPI_ISL_11310221	EPI_ISL_11310235	EPI_ISL_11310241	EPI_ISL_11310248
EPI_ISL_11310258	EPI_ISL_11310271	EPI_ISL_11310298	EPI_ISL_11310309	EPI_ISL_11310311
EPI_ISL_11310315	EPI_ISL_11310322	EPI_ISL_11310338	EPI_ISL_11310341	EPI_ISL_11310349
EPI_ISL_11310377	EPI_ISL_11310381	EPI_ISL_11310383	EPI_ISL_11310386	EPI_ISL_11310388
EPI_ISL_11310391	EPI_ISL_11310403	EPI_ISL_11310476	EPI_ISL_11310480	EPI_ISL_11310498
EPI_ISL_11310502	EPI_ISL_11310509	EPI_ISL_11310518	EPI_ISL_11310523	EPI_ISL_11310530
EPI_ISL_11310537	EPI_ISL_11311257	EPI_ISL_11311266	EPI_ISL_11311284	EPI_ISL_11311297
EPI_ISL_11311316	EPI_ISL_11311328	EPI_ISL_11311351	EPI_ISL_11311361	EPI_ISL_11311362
EPI_ISL_11311366	EPI_ISL_11311370	EPI_ISL_11311380	EPI_ISL_11311387	EPI_ISL_11311391
EPI_ISL_11311392	EPI_ISL_11311396	EPI_ISL_11311399	EPI_ISL_11311404	EPI_ISL_11311409
EPI_ISL_11311429	EPI_ISL_11311676	EPI_ISL_11311690	EPI_ISL_11311727	EPI_ISL_11311761
EPI_ISL_11311775	EPI_ISL_11311782	EPI_ISL_11311783	EPI_ISL_11311789	EPI_ISL_11311792
EPI_ISL_11311794	EPI_ISL_11311806	EPI_ISL_11311830	EPI_ISL_11311831	EPI_ISL_11311849
EPI_ISL_11311857	EPI_ISL_11311859	EPI_ISL_11311862	EPI_ISL_11311904	EPI_ISL_11311906
EPI_ISL_11311909	EPI_ISL_11311914	EPI_ISL_11312535	EPI_ISL_11312569	EPI_ISL_11312594
EPI_ISL_11312595	EPI_ISL_11312601	EPI_ISL_11312614	EPI_ISL_11312617	EPI_ISL_11326825
EPI_ISL_11326829	EPI_ISL_11326850	EPI_ISL_11326868	EPI_ISL_11326883	EPI_ISL_11326889
EPI_ISL_11326897	EPI_ISL_11340526	EPI_ISL_11344186	EPI_ISL_11348711	EPI_ISL_11370636
EPI_ISL_11372632	EPI_ISL_11372670	EPI_ISL_11372675	EPI_ISL_11372688	EPI_ISL_11457340
EPI_ISL_11457353	EPI_ISL_11457361	EPI_ISL_11457364	EPI_ISL_11457367	EPI_ISL_11457378
EPI_ISL_11457380	EPI_ISL_11457382	EPI_ISL_11457392	EPI_ISL_11457400	EPI_ISL_11457436
EPI_ISL_11495203	EPI_ISL_11495231	EPI_ISL_11495237	EPI_ISL_11495281	EPI_ISL_11495286
EPI_ISL_11495312	EPI_ISL_11495324	EPI_ISL_11495328	EPI_ISL_11496111	EPI_ISL_11496144
EPI_ISL_11496161	EPI_ISL_11496166	EPI_ISL_11496168	EPI_ISL_11496172	EPI_ISL_11496174
EPI_ISL_11496176	EPI_ISL_11496186	EPI_ISL_11496190	EPI_ISL_11496214	EPI_ISL_11496223
EPI_ISL_11514323	EPI_ISL_11514326	EPI_ISL_11514344	EPI_ISL_11514363	EPI_ISL_11514366
EPI_ISL_11514372	EPI_ISL_11514376	EPI_ISL_11560431	EPI_ISL_11560432	EPI_ISL_11560444
EPI_ISL_11560445	EPI_ISL_11560454	EPI_ISL_11560497	EPI_ISL_11560508	EPI_ISL_11560527
EPI_ISL_11560551	EPI_ISL_11560561	EPI_ISL_11560574	EPI_ISL_11560579	EPI_ISL_11560580
EPI_ISL_11560582	EPI_ISL_11560619	EPI_ISL_11560626	EPI_ISL_11560627	EPI_ISL_11560636
EPI_ISL_11560641	EPI_ISL_11697678	EPI_ISL_11697691	EPI_ISL_11697704	EPI_ISL_11697720

EPI_ISL_11697792	EPI_ISL_11697793	EPI_ISL_11697834	EPI_ISL_11697842	EPI_ISL_11697844
EPI_ISL_11697860	EPI_ISL_11697900	EPI_ISL_11697906	EPI_ISL_11756960	EPI_ISL_11756961
EPI_ISL_11756962	EPI_ISL_11756965	EPI_ISL_11756978	EPI_ISL_11756996	EPI_ISL_11756998
EPI_ISL_11757023	EPI_ISL_11757029	EPI_ISL_11757032	EPI_ISL_11757035	EPI_ISL_11757114
EPI_ISL_11757120	EPI_ISL_11757152	EPI_ISL_11757156	EPI_ISL_11757167	EPI_ISL_11757172
EPI_ISL_11757174	EPI_ISL_11757178	EPI_ISL_11757180	EPI_ISL_11757184	EPI_ISL_11757202
EPI_ISL_11757238	EPI_ISL_11757272	EPI_ISL_11757445	EPI_ISL_11757472	EPI_ISL_11757494
EPI_ISL_11757518	EPI_ISL_11757526	EPI_ISL_11757543	EPI_ISL_11757552	EPI_ISL_11757576
EPI_ISL_11757579	EPI_ISL_11757585	EPI_ISL_11757598	EPI_ISL_11757599	EPI_ISL_11757603
EPI_ISL_11757659	EPI_ISL_11757692	EPI_ISL_11793410	EPI_ISL_11798744	EPI_ISL_11798768
EPI_ISL_11798774	EPI_ISL_11798796	EPI_ISL_11798808	EPI_ISL_11798822	EPI_ISL_11798828
EPI_ISL_11798831	EPI_ISL_11798832	EPI_ISL_11798866	EPI_ISL_11798879	EPI_ISL_11798880
EPI_ISL_11798940	EPI_ISL_11798948	EPI_ISL_11798954	EPI_ISL_11798983	EPI_ISL_11809301
EPI_ISL_11880851	EPI_ISL_11880873	EPI_ISL_11880884	EPI_ISL_11880907	EPI_ISL_11880936
EPI_ISL_11880940	EPI_ISL_11880957	EPI_ISL_11880960	EPI_ISL_11880989	EPI_ISL_11881007
EPI_ISL_11881013	EPI_ISL_11881026	EPI_ISL_11881034	EPI_ISL_11881043	EPI_ISL_11881057
EPI_ISL_11881060	EPI_ISL_11881067	EPI_ISL_11881107	EPI_ISL_11883642	EPI_ISL_11883657
EPI_ISL_11883678	EPI_ISL_11883708	EPI_ISL_11884367	EPI_ISL_11884370	EPI_ISL_11884394
EPI_ISL_11884395	EPI_ISL_11884520	EPI_ISL_11884527	EPI_ISL_11884547	EPI_ISL_11884555
EPI_ISL_11884559	EPI_ISL_11884585	EPI_ISL_11884586	EPI_ISL_11884595	EPI_ISL_11884596
EPI_ISL_11884605	EPI_ISL_11884611	EPI_ISL_11884621	EPI_ISL_11884627	EPI_ISL_11884632
EPI_ISL_11884638	EPI_ISL_11884684	EPI_ISL_11888738	EPI_ISL_11888739	EPI_ISL_11888742
EPI_ISL_11888752	EPI_ISL_11888806	EPI_ISL_11888808	EPI_ISL_11888814	EPI_ISL_11888817
EPI_ISL_11890353	EPI_ISL_11890362	EPI_ISL_11890365	EPI_ISL_11890413	EPI_ISL_11890424
EPI_ISL_11890426	EPI_ISL_11905989	EPI_ISL_11906009	EPI_ISL_11906024	EPI_ISL_11906033
EPI_ISL_11906038	EPI_ISL_11906050	EPI_ISL_11906082	EPI_ISL_11906102	EPI_ISL_11906122
EPI_ISL_11906132	EPI_ISL_11906155	EPI_ISL_11906225	EPI_ISL_11906255	EPI_ISL_11906282
EPI_ISL_11906308	EPI_ISL_11906332	EPI_ISL_11906339	EPI_ISL_11906364	EPI_ISL_11941024
EPI_ISL_12082786	EPI_ISL_12082820	EPI_ISL_12082826	EPI_ISL_12082895	EPI_ISL_12082915
EPI_ISL_12082921	EPI_ISL_12082922	EPI_ISL_12082937	EPI_ISL_12082941	EPI_ISL_12082943
EPI_ISL_12082947	EPI_ISL_12082964	EPI_ISL_12083005	EPI_ISL_12083008	EPI_ISL_12083014
EPI_ISL_12089600	EPI_ISL_12089622	EPI_ISL_12089625	EPI_ISL_12089641	EPI_ISL_12089667
EPI_ISL_12090333	EPI_ISL_12090395	EPI_ISL_12090406	EPI_ISL_12090449	EPI_ISL_12133289
EPI_ISL_12133304	EPI_ISL_12133379	EPI_ISL_12133426	EPI_ISL_12133429	EPI_ISL_12133456
EPI_ISL_12133483	EPI_ISL_12133528	EPI_ISL_12133551	EPI_ISL_12231718	EPI_ISL_12231727
EPI_ISL_12231792	EPI_ISL_12231836	EPI_ISL_12231844	EPI_ISL_12231848	EPI_ISL_12231854
EPI_ISL_12231907	EPI_ISL_12231938	EPI_ISL_12231941	EPI_ISL_12232745	EPI_ISL_12232746
EPI_ISL_12232760	EPI_ISL_12232797	EPI_ISL_12232851	EPI_ISL_12232860	EPI_ISL_12232947
EPI_ISL_12232999	EPI_ISL_12263409	EPI_ISL_12263428	EPI_ISL_12263432	EPI_ISL_12263471
EPI_ISL_12263473	EPI_ISL_12263492	EPI_ISL_12263510	EPI_ISL_12263531	EPI_ISL_12263535
EPI_ISL_12263574	EPI_ISL_12263602	EPI_ISL_12263611	EPI_ISL_12263626	EPI_ISL_12263640
EPI_ISL_12264356	EPI_ISL_12264386	EPI_ISL_12264406	EPI_ISL_12264416	EPI_ISL_12352666
EPI_ISL_12352740	EPI_ISL_12355745	EPI_ISL_12358861	EPI_ISL_12364710	EPI_ISL_12407363
EPI_ISL_12407367	EPI_ISL_12492927	EPI_ISL_12492932	EPI_ISL_12492945	EPI_ISL_12492952
EPI_ISL_12492962	EPI_ISL_12492982	EPI_ISL_12492992	EPI_ISL_12493019	EPI_ISL_12493041
EPI_ISL_12493051	EPI_ISL_12525604	EPI_ISL_12525703	EPI_ISL_12525713	EPI_ISL_12525762
EPI_ISL_12525787	EPI_ISL_12528304	EPI_ISL_12528329	EPI_ISL_12528349	EPI_ISL_12528351
EPI_ISL_12528352	EPI_ISL_12528374	EPI_ISL_12528399	EPI_ISL_12528417	EPI_ISL_12528441
EPI_ISL_12528447	EPI_ISL_12528464	EPI_ISL_12528485	EPI_ISL_12528487	EPI_ISL_12528493
EPI_ISL_12528520	EPI_ISL_12528541	EPI_ISL_12528574	EPI_ISL_12532565	EPI_ISL_12532718
EPI_ISL_12562357	EPI_ISL_12620239	EPI_ISL_12620285	EPI_ISL_12620332	EPI_ISL_12620362
EPI_ISL_12620401	EPI_ISL_12620449	EPI_ISL_12650519	EPI_ISL_12684701	EPI_ISL_12684737
EPI_ISL_12684793	EPI_ISL_12685050	EPI_ISL_12685055	EPI_ISL_12685062	EPI_ISL_12685081
EPI_ISL_12685120	EPI_ISL_12685122	EPI_ISL_12704709	EPI_ISL_12704728	EPI_ISL_12704742
EPI_ISL_12704785	EPI_ISL_12851830	EPI_ISL_12851859	EPI_ISL_12851898	EPI_ISL_12851937

EPI_ISL_12852055	EPI_ISL_12852074	EPI_ISL_12872712	EPI_ISL_13013418	EPI_ISL_13013556
EPI_ISL_13013607	EPI_ISL_13177670	EPI_ISL_13177714	EPI_ISL_13177718	EPI_ISL_13177722
EPI_ISL_13432480	EPI_ISL_13445421	EPI_ISL_13445445	EPI_ISL_13445489	EPI_ISL_13695387
EPI_ISL_13695401	EPI_ISL_13695407	EPI_ISL_13865279	EPI_ISL_13869147	EPI_ISL_13905107
EPI_ISL_13905248	EPI_ISL_13980992	EPI_ISL_13981033	EPI_ISL_14026800	EPI_ISL_14156361
EPI_ISL_14156391	EPI_ISL_14156403	EPI_ISL_14156434	EPI_ISL_14156444	EPI_ISL_14191366
EPI_ISL_14199109	EPI_ISL_14199188	EPI_ISL_14199196	EPI_ISL_14199400	EPI_ISL_14471135
EPI_ISL_14550042	EPI_ISL_14550136	EPI_ISL_14550277	EPI_ISL_14550429	EPI_ISL_14550433
EPI_ISL_14551523	EPI_ISL_14551575	EPI_ISL_14551604	EPI_ISL_14551706	EPI_ISL_14551738
EPI_ISL_14585159	EPI_ISL_14585222	EPI_ISL_14585327	EPI_ISL_14585400	EPI_ISL_14585415
EPI_ISL_14585436	EPI_ISL_14585476	EPI_ISL_14585485	EPI_ISL_14681311	EPI_ISL_14681313
EPI_ISL_14681337	EPI_ISL_14681359	EPI_ISL_14681394	EPI_ISL_14756230	EPI_ISL_14756279
EPI_ISL_16428847				
